# Stable Pincer Gold(III)‐TADF Emitters with Extended Donor–Acceptor Separation for Efficient Vacuum‐Deposited OLEDs with Operational Lifetime (LT_95_) up to 3831 h at 1000 cd m^−2^

**DOI:** 10.1002/advs.202502529

**Published:** 2025-04-26

**Authors:** Hui‐Xing Shu, Shuo Xu, Wai‐Pong To, Gang Cheng, Chi‐Ming Che

**Affiliations:** ^1^ Department of Chemistry State Key Laboratory of Synthetic Chemistry CAS‐HKU Joint Laboratory on New Materials The University of Hong Kong Pokfulam Road Hong Kong SAR P. R. China; ^2^ Hong Kong Quantum AI Lab Limited Units 909–915, Building 17 W, 17 Science Park West Avenue Hong Kong Science Park, Pak Shek Kok Hong Kong SAR P. R. China; ^3^ HKU Shenzhen Institute of Research and Innovation Shenzhen Guangdong 518057 P. R. China

**Keywords:** gold, OLEDs, operational lifetime, singlet–triplet energy gap, thermally activated delayed fluorescence

## Abstract

Although gold‐TADF (thermally activated delayed fluorescence) emitters have attractive prospects as next‐generation practical OLED emitters, the performance of OLEDs utilizing gold(I)‐ and gold(III)‐TADF emitters lags behind the requirements of practical applications, and device lifetime has become a bottleneck. Here, novel pincer gold(III)‐TADF emitters that are easily fabricated with tunable donor and acceptor ligands are presented. These pincer gold(III)‐TADF emitters exhibit an extended molecular π‐distance along the transition dipole moment, resulting in a significant reduction in the electron exchange energy between the S_1_ and T_1_ excited states, thus narrowing the singlet–triplet energy gap (Δ*E*_ST_). The combination of small Δ*E*_ST_ and heavy‐atom (Au, S) effect greatly enhances spin‐flip dynamics and produces efficient TADF (photoluminescence quantum yields up to 90%) with high radiative decay rate constants (*k*_r_ up to 10^6^ s^−1^), and short lifetimes (*τ* less than 1.2 µs) in thin films at room temperature. Vacuum‐deposited OLEDs based on these gold(III)‐TADF emitters demonstrate impressive stability, achieving i) a high maximum external quantum efficiency (EQE_max_) of up to 22.2%, and ii) a record‐ long operational lifetime (LT_95_) of 3831 h at an initial luminance of 1000 cd m^−2^. This excellent durability makes the pincer gold(III)‐TADF emitter a promising and competitive alternative to iridium and platinum emitters for practical OLED applications.

## Graphical abstract

Novel pincer gold(III)‐TADF (thermally activated delayed fluorescence) emitters with extended π‐distances exhibit small singlet‐triplet energy gaps (Δ*E*_ST_), efficient reverse intersystem crossing (RISC), enhanced radiative decay rates and improved excitons utilization, leading to efficient electroluminescence. The record‐long operational lifetime (LT_95@1000_ = 3831 h) demonstrates their excellent durability in OLEDs, supporting the feasibility of developing innovative pincer‐gold‐TADF emitters for practical applications.

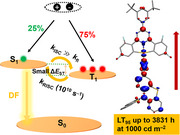

## 1 Introduction

**Scheme 1:**
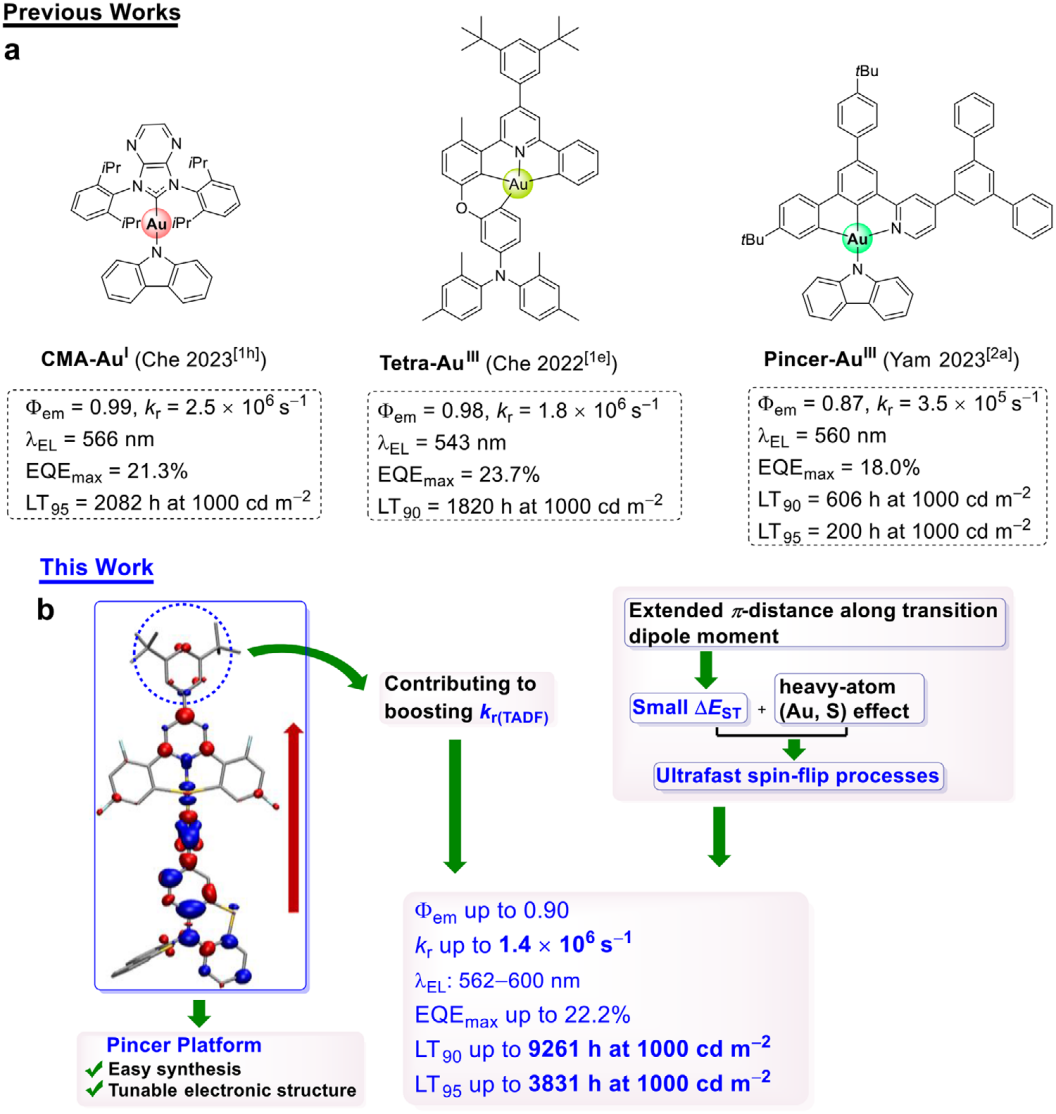
a) Selected examples of Au^I^‐TADF, tetradentate, and pincer Au^III^‐TADF emitters. b) Molecular design concepts and schematic diagram of pincer gold(III)‐TADF emitters in this work (The structure of **3** indicates its transition density upon excitation, and the red arrow illustrates the direction of the transition dipole moment).

Since gold atoms have large spin‐orbit coupling constants and are known to form strong gold‐ligand bonds, gold‐TADF emitters can in principle become thermally stable and have fast reverse intersystem crossing rate constants, making such emitters an attractive new alternative to platinum and iridium emitters/sensitizers in OLED applications. Despite decades of research, the reported performance of gold‐OLEDs^[^ ^1^, ^2^, ^3^ ^]^ still lags far behind the requirements for practical applications, with device lifetime becoming a bottleneck issue. Although relatively stable gold‐OLEDs using tetradentate gold(III)‐TADF emitters have been recently reported,^[^ ^1^, ^2^, ^4^ ^]^ the synthesis and post‐synthetic modification of these emitters are difficult and complex. This is in contrast to the facile synthesis of stable tetradentate Pt^II^ emitters^[^ ^5^ ^]^ via a direct “one metal ion + one ligand” metalation strategy. The multi‐step synthetic routes required for tetradentate gold(III) emitters pose significant challenges and hinder the development of customized gold emitters with tunable photophysical properties. Among the reported gold‐emitters for OLEDs, luminescent pincer gold(III) complexes have been intensively studied.^[^ ^1^, ^2^, ^6^ ^]^ Pincer complexes, including pincer gold(III) complexes, are recognized for their ease of synthesis (modular synthesis) and tunable electronic properties and have applications in various fields including catalysis, medicinal chemistry, and material science.^[^ ^7^ ^]^ However, the best OLED to date using pincer gold(III) complexes as emitters has a LT_95_ value of only 200 h at an initial luminance (L_0_) of 1000 cd m^−2^ (along with a maximun external quantum efficiency (EQE_max_) of 18.0%, **Scheme** **1**a).^[^ ^2a^ ^]^ In this context, we envision developing stable and efficient gold(III)‐TADF emitters by integrating the remarkable photophysical properties of organic donor–acceptor (D‐A) TADF materials into luminescent pincer gold(III) complexes. Our previous studies revealed that modification of the *para*‐position of the pyridine ring in gold(III)‐TADF emitters containing cyclometalated [C^N^C] ligands can significantly increase the radiative decay rate constant.^[^ ^1c–e^ ^]^ Furthermore, extending the π‐distance along the transition dipole moment in the D‐A TADF emitter is anticipated to effectively diminish the singlet–triplet energy gap (Δ*E*_ST_).^[^ ^1^, ^8^ ^]^ Building on these design concepts, the gold(III) emitter in this study utilizes i) a pincer [C^N^C] ligand (acceptor) with a 3,5‐di‐*tert*‐butylphenyl group in the *para*‐position of the pyridine ring and ii) aromatic alkynyl ligand (donor), thereby creating an extended molecular π‐distance along the transition dipole moment of the D‐A type emitter (Scheme 1b). As expected, this series of pincer gold(III) emitters exhibit efficient TADF emission with PLQY values as high as 0.90, decay lifetimes of ˂1.2 µs, and *k*_r_ values reaching 10^6^ s^−1^ in thin films at room temperature. Vacuum‐deposited OLEDs using these gold(III)‐TADF complexes as emitters achieved EQE_max_ up to 22.2% and efficiency roll‐off down to 2.3% at 1000 cd m^−2^. Notably, under our laboratory conditions, a device based on complex **5** achieved an excellent LT_95_ of 3831 h at an L_0_ of 1000 cd m^−2^. This record‐breaking device lifetime represents a major breakthrough for gold OLEDs and is one of the longest operational lifetimes reported to date for non‐iridium OLEDs.^[^ ^1^, ^5^, ^9^ ^]^

## 2 Results and Discussion

### 2.1 Synthesis and Characterization

**Scheme 2:**
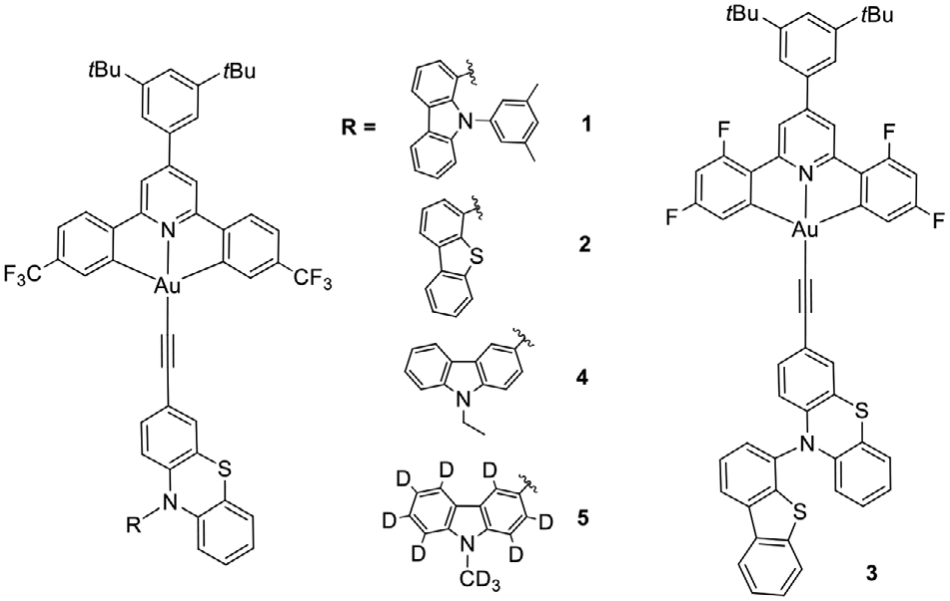
Molecular structures of pincer gold(III)‐TADF complexes **1**–**5**.

**Scheme** **2** shows the molecular structures of gold(III) alkynyl complexes **1**–**5**. Detailed synthesis procedures are provided in the Supporting Information (Schemes S1 and S2). The complexes were characterized by ^1^H NMR, ^13^C NMR spectroscopy, and high‐resolution mass spectrometry (HRMS). C*_α_* ≡C*_β_*−Au^III^ ligation was confirmed, with the C*_β_* nucleus coordinated to Au^III^ appearing at 89 ± 1 ppm and the C*_α_* nucleus at 100 ± 1 ppm. Notably, these complexes exhibit excellent thermal stability with decomposition temperature (*T*_d_, defined as the temperature at which the complex shows a 5% weight loss) as high as 404 °C under nitrogen atmosphere (Figure S1, Supporting Information).

A single crystal of complex **5** was obtained and subjected to X‐ray diffraction analysis (Figure S2, Supporting Information), revealing a distorted square‐planar geometry around the central Au atom and near‐coplanar orientation between the mean phenothiazinyl and [C^N^C] ligand planes. The intermolecular head‐to‐tail stacking at a distance of 3.44 Å suggests weak π–π interactions within the solid state.

The electrochemical properties of **1**, **2**, and **4** were examined by cyclic voltammetry (CV) and differential pulse voltammetry (DPV) in *N*,*N*‐dimethylformamide. As shown in Figure S3 and Table S4 (Supporting Information), complexes **1**, **2**, and **4** exhibit reversible to quasi‐reversible redox couples in both reduction and oxidation, indicating that the electrochemically generated radical cations or anions of these gold(III) complexes are less susceptible to further chemical reactions. The gold(III) complexes demonstrate alkynyl ligand‐centered oxidation, with oxidation potentials significantly influenced by the *N*‐substituent of the alkynyl ligand, following the order **1** (E_ox_ = 0.70 V) < **4** (E_ox_ = 0.73 V) < **2** (E_ox_ = 0.83 V). In contrast, they show nearly identical reduction potentials at ≈−1.12 V (vs SCE), consistent with the [C^N^C] ligand‐centered reduction process. The satisfactory electrochemical stability of this class of gold(III) complexes is beneficial to increasing the operational stability of OLEDs prepared using them as emitters/sensitizers.^[^ ^10^ ^]^

### 2.2 Photophysical Properties

**Figure 1:**
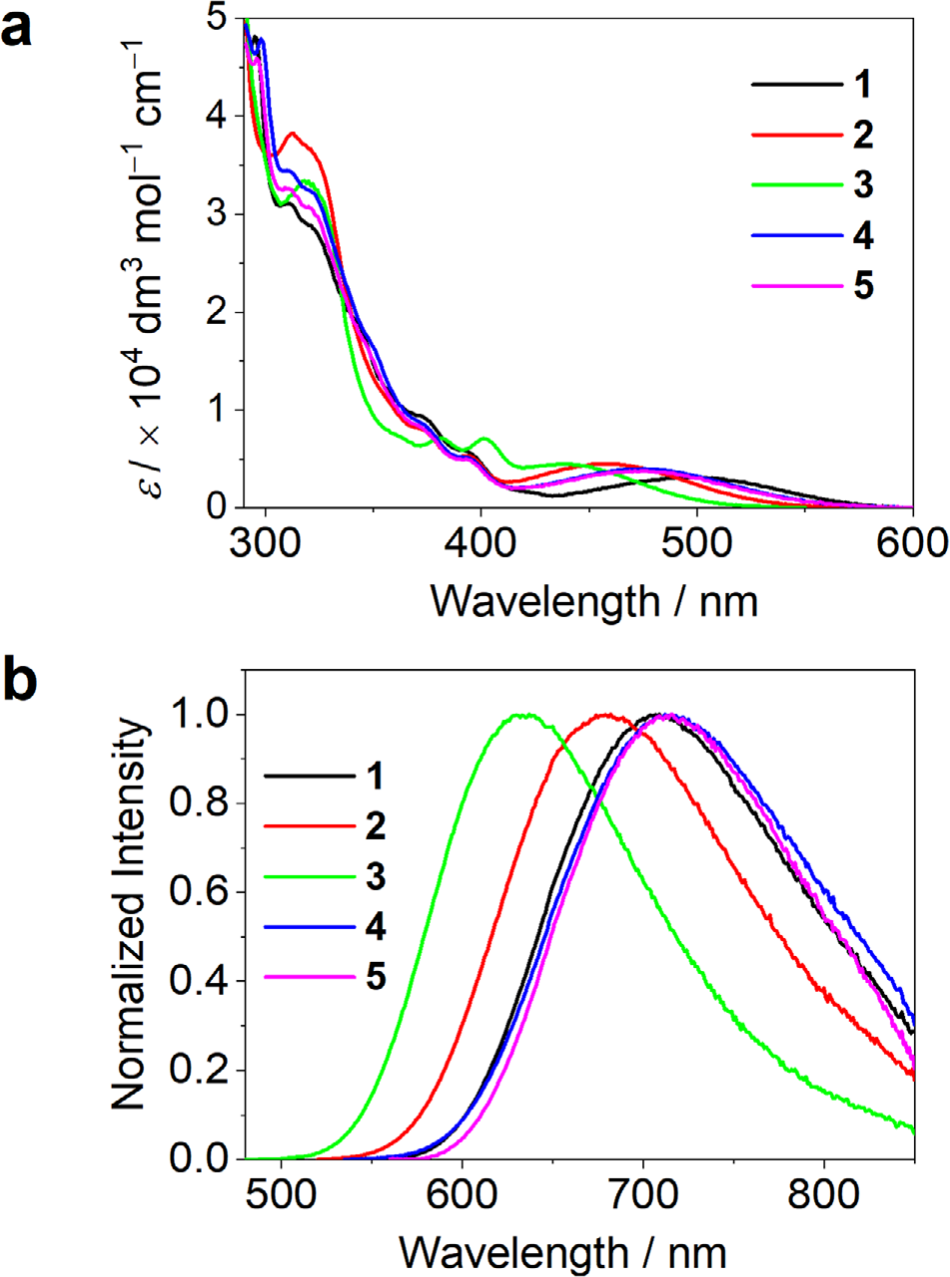
a) UV–vis absorption and b) emission spectra of **1**–**5** in toluene at room temperature.

The UV‐visible absorption and emission spectra of **1**–**5** in toluene at room temperature are shown in **Figure** **1** and the corresponding data are summarized in **Table** **1**. These complexes exhibit intense absorption (*ε*: 2.5–5.0 × 10^4^ mol^−1^ dm^3^ cm^−1^) at 300–325 nm (Band III) and moderately intense absorption (*ε*: 5.0–9.1 × 10^3^ mol^−1^ dm^3^ cm^−1^) at 370–405 nm (Band II). Band II is attributed to the characteristic absorption of the [C^N^C] backbone, exhibiting a vibrational spacing of 1300–1600 cm^−1^.^[^ ^11^ ^]^ Band III and band II can be assigned to localized electronic transitions within the alkynyl and tridentate [C^N^C] ligands, respectively. Furthermore, the broad band with moderately intense absorption (*ε*: 3.1–4.5 × 10^3^ mol^−1^ dm^3^ cm^−1^) at 439–500 nm (Band I) can be assigned to the ligand‐to‐ligand‐charge‐transfer (^1^LLCT) transition from π (alkynyl ligand) to π* ([C^N^C] ligand). Structural modifications to the alkynyl ligand's *N*‐substituent or [C^N^C] backbone modulate donor‐acceptor strength, inducing pronounced shifts in band I. This trend corroborates the assignment of ^1^LLCT transition in band I.

**Table 1:** Photophysical data of **1**–**5** at room temperature.

Emitter	Absorption	Emission	
	*λ*_abs_ [nm]^a)^ (*ɛ* [× 10^3^ mol^−1^ dm^3^ cm^−1^])	In toluene *λ*_em_ [nm]^a)^ (*τ* [µs]; *Φ* [%]^b)^; *k*_r_ [10^5^ s^−1^], *k*_nr_ [10^5^ s^−1^])	4 wt% in mCP thin films *λ*_em_ [nm] (*τ* [µs]; *Φ* [%]^b)^; *k*_r_ [10^5^ s^−1^], *k*_nr_ [10^5^ s^−1^])
**1**	295 (48.3), 310 (31.2), 321 (sh, 28.8), 375 (sh, 9.1), 395 (sh, 5.7), 422 (sh, 1.6), 500 (br, 3.1)	707 (0.029, 2.9, 10.0, 334.8)	605 (0.86, 90, 10.5, 1.2)
**2**	312 (38.2), 371 (sh, 8.1), 393 (5.4), 461 (br, 4.5)	678 (0.052, 7.0, 13.5, 178.8)	585 (1.01, 86, 8.5, 1.4)
**3**	317 (33.5), 382 (7.1), 401 (7.1), 439 (br, 4.5)	634 (0.37, 41.9, 11.3, 15.7)	572 (1.13, 88, 7.8, 1.1)
**4**	298 (47.9), 311 (34.5), 322 (32.3), 372 (sh, 8.6), 393 (5.2), 475 (br, 4.0)	714 (0.02, 2.6, 13.0, 487.0)	605 (0.99, 81, 8.2, 1.9)
**5**	296 (45.9), 311 (32.6), 320 (sh, 30.8), 372 (sh, 8.2), 393 (5.0), 474 (br, 3.8)	713 (0.024, 2.6, 10.8, 405.8)	604 (0.96, 81, 8.4, 2.0)

^a)^ In deaerated toluene (2 × 10^−5^ м), “sh” stands for shoulder, “br” stands for broad;

^b)^ absolute emission quantum yields.

Upon photo‐excitation, all complexes display broad, structureless emission bands in toluene (*λ*_max_: 634–714 nm) at room temperature. Complex **3**, bearing the 4F‐substituted [C^N^C] ligand, demonstrates the highest emission energy (*λ*_max_: 634 nm) and photoluminescence quantum yield (PLQY: 42%), and the longest decay lifetime (*τ*: 0.37 µs). A progressive red‐shift in emission maxima is observed from **3** (634 nm) to **2** (678 nm), **1** (707 nm), **5** (713 nm), and **4** (714 nm), accompanied by a marked decline in both PLQY (7.0% for **2**, 2.9% for **1**, 2.6% for **4** and **5**) and *τ* (52 ns for **2**, 29 ns for **1**, 24 ns for **5**, 20 ns for **4**). The radiative (*k*_r_) and non‐radiative (*k*_nr_) decay rate constants were estimated using the formulae *k*_r_ = *Φ*/*τ* and *k*_nr_ = (1‐*Φ*)/*τ* (Table 1). All gold(III) complexes exhibit comparable radiative decay efficiency, with small variations in the *k*_r_ values (1.0–1.4 × 10^6^ s^−1^). On the other hand, *k*_nr_ is significantly related to the emission energy, with the values increasing from 1.6 × 10^6^ s^−1^ (**3**) to 1.8 × 10^7^ s^−1^ (**2**), 3.3 × 10^7^ s^−1^ (**1**), 4.1 × 10^7^ s^−1^ (**5**), and 4.9 × 10^7^ s^−1^ (**4**). This can be rationalized through the energy gap law, which shows that as the emission gap narrows, non‐radiative decay dominates. These gold(III) complexes exhibit pronounced solvatochromism, with emission energies sensitive to solvent polarity. As demonstrated by **2** and **4** (Figures S6 and S7, Supporting Information), the Lippert–Mataga analyses show pronounced positive slopes of 16 805 and 5923 cm^−1^, respectively, indicating dominant charge‐transfer (CT) excited‐state characteristics. Upon doping into mCP (1,3‐bis(N‐carbazolyl)benzene) thin films, the non‐radiative decay pathways in these complexes are largely suppressed, lowering *k*_nr_ to 10^5^ s^−1^. As a result, the PLQY is significantly improved, reaching 81–90%. Similar to the reported D‐A type TADF emitters with multiple conformers,^[^ ^12^ ^]^ the emission of complexes **1**–**5** in mCP thin films exhibits bi‐exponential decay with emission lifetimes averaging 0.86–1.13 µs. The observed PL performance (*k*_r_ values up to 10^6^ s^−1^, PLQYs up to 90%, short emission lifetimes less than 1.2 µs, and significant excited‐state CT character) aligns closely with previously reported gold(III) counterparts exhibiting TADF.^[^ ^1^, ^2^ ^]^ The electronic structure of the pincer D‐A gold(III)‐TADF complex is highly tunable. Due to spatial and electronic synergistic effects, even minor modifications to the molecular framework can significantly change its electronic properties. As previous studies have shown,^[^ ^1^, ^2^, ^6^ ^]^ substituent engineering, such as the introduction of electron‐donating groups (e.g., −NMe_2_, −OEt) on the *para*‐pyridine ring or the alternation of the donor ligands (e.g., *p*‐NPh_2_−), can precisely control the HOMO‐LUMO gap, allowing the emission wavelengths to be tuned over a wide range (480–720 nm). These results highlight the versatility of the pincer D‐A gold(III)‐TADF complex, where ligand and substituent design are powerful tools to tune optoelectronic properties.

**Figure 2:**
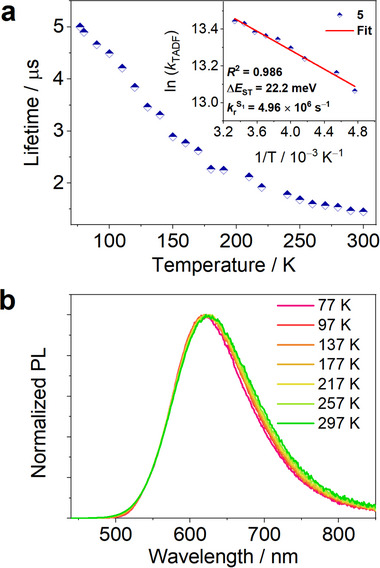
a) Temperature‐dependent plot of emission lifetime of **5** in 2 wt% PMMA film; Inset: Arrhenius fit of the *k*_TADF_ value versus temperature. b) Normalized temperature‐dependent PL spectra of **5** in 2 wt% PMMA film.

To gain more insight into the TADF properties of these gold(III) complexes, variable‐temperature (VT) (77 to 300 K) transient PL decay measurements of **1**–**5** in PMMA (poly(methyl methacrylate)) thin films were performed, as shown in **Figures** **2**a and S8 (Supporting Information). Taking **5** as an example, assuming a three‐state kinetic model involving S_0_, ^1^LLCT, and ^3^LLCT excited states, the Arrhenius plot shows that ln*k*_TADF_ is linearly dependent on 1/*T* above 200 K, with a slope corresponding to Δ*E*_ST_/*k*_B_. The Δ*E*_ST_ value of **5** was determined to be 22.2 meV, and those of **1**–**4** were estimated to be 36.7, 30.2, 36.8, and 33.6 meV, respectively (Figure S8, Supporting Information). These small Δ*E*_ST_ values (≤36.8 meV) may promote delayed emission even at 77 K, as demonstrated by complex **5**, which retains a broad, featureless emission band and a relatively short decay lifetime of 5.0 µs at 77 K (Figure 2b).

### 2.3 Transient Absorption and Time‐Resolved Fluorescence Spectroscopy

**Figure 3:**
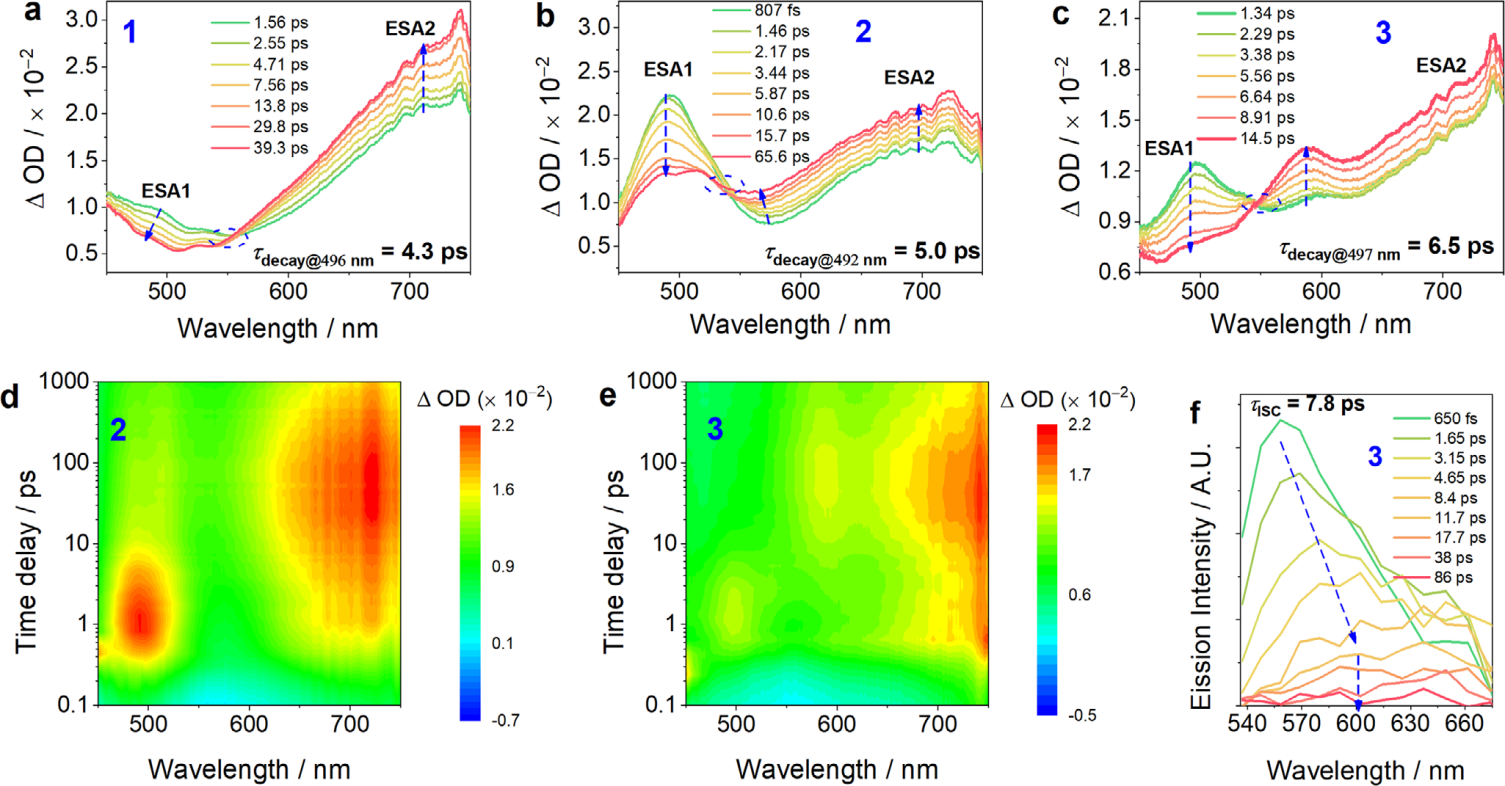
fs‐TA difference spectra of a) **1**, b) **2**, and c) **3** in toluene solution (3.0 × 10^−4^ mol dm^−3^) under 370 nm pulse excitation. fs‐TA contour map of d) **2** and e) **3** in toluene solution under 370 nm pulse‐excitation. f) fs‐TRF spectra of **3** in toluene solution (2.0 × 10^−3^ mol dm^−3^) under 400 nm pulse excitation.

To investigate the early excited‐state dynamics of the gold(III)‐TADF complexes, femtosecond transient absorption (fs‐TA) and time‐resolved fluorescence (fs‐TRF) spectra of **1**–**4** in toluene were acquired at room temperature. As shown in **Figures** **3** and S10 (Supporting Information), complexes **1**–**4** showed similar spectral transformations under 370 nm pulse excitation. The initially formed fs‐TA spectrum (0.6–1.56 ps) showed a positive excited state absorption (ESA1) spanning 450–750 nm. Subsequently, this transient feature evolved to the developed‐state of ESA2 within 25–65.6 ps, which is characterized by a gradual decay of the band below 540 nm and a simultaneous growth of the band above 555 nm. Clear isosbestic spectral changes in the range of 540–555 nm can be observed during the temporal evolution, which is characteristic of dynamic conversion from S_1_ to the triplet manifold, i.e., intersystem crossing (ISC). Kinetic analyses of TA time traces revealed decay time constants in the range of 4.3–6.5 ps. The similarity in spectral shape and peaks between late‐time ESA2 (Figure S10, Supporting Information) and early‐time nanosecond transient absorption (ns‐TA) (Figure S9, Supporting Information) confirms that this spectral conversion arises from ISC. Further analysis of the fs‐TA contour map (Figure 3d,f; Figure S10, Supporting Information) reveals that structural tuning of the [C^N^C] backbone (**2**, **3**) modulates the ESA evolution in the 550–650 nm region. On the other hand, donor moiety *N*‐substituent variation (**1**, **2**, and **4**) governs the ESA evolution below 540 nm. Figure 3f and Figure S11 (Supporting Information) depict the temporal evolution of fs‐TRF of **1** and **3**. Pulse‐excitation of **1** and **3** in toluene at 400 nm generated broadband prompt fluorescence (PF), with early‐time dynamic

Stokes shifts arising from structural reorganization and/or vibrational cooling following excitation. Since the S_1_ → S_0_ transition (*τ* ^S^ = 1/(*k*_r_^S^ + *k*_nr_^S^)) typically proceeds on the nanosecond timescale, the picosecond decay observed in the latter TRF (**1**: 9.2 ps, **3**: 7.8 ps) indicates that ISC is responsible for the depopulation of the S_1_ state. Here, *τ*_PF_ = 1/(*k*_ISC_ + *k*_r_^S^ + *k*_nr_^S^), enabling approximation of the ISC rate constant as *k*_ISC_ ≈ 1/*τ*_PF_. Using this relationship, *k*_ISC_ was calculated as 1.1 × 10^11^ s^−1^ for **1**, and 1.3 × 10^11^ s^−1^ for **3**. Notably, the *τ*_PF_ values of **1** and **3** are consistent with the corresponding TA decay values obtained from the fs‐TA experiments. Therefore, the *k*_ISC_ values for **2** and **4** can be estimated to be 2.0 × 10^11^ s^−1^ and 2.1 × 10^11^ s^−1^, respectively. Using the experimentally determined Δ*E*_ST_ value (vide supra) and ISC rate constant, we can further estimate the T_1_ → S_1_ reverse intersystem crossing (RISC) rate constant (*k*_RISC_) for this family of complexes through the steady‐state approximation.^[^ ^1^, ^13^ ^]^

(1) $$\frac{k_{\mathrm{RISC}}}{k_{\mathrm{ISC}}}\approx \frac{1}{3}\exp\left(-\frac{\Delta E_{\mathrm{ST}}}{k_{B}T}\right)$$

The lack of energetically close high‐lying triplet excited states above the lowest ^3^LLCT excited state, as evidenced by the absence of pronounced triplet ^3^IL emission at 77 K (vide supra), supports the reliability of the three‐state model. Therefore, the *k*_RISC_ for **1**–**4** were then estimated as 8.8 × 10^9^ s^−1^ (**1**), 2.1 × 10^10^ s^−1^ (**2**), 1.0 × 10^10^ s^−1^ (**3**), and 2.9 × 10^10^ s^−1^ (**4**). These *k*_RISC_ values surpass those of tetradentate Au^III^‐TADF (*k*_RISC_ = 0.5–2.0 × 10^9^ s^−1^),^[^ ^1e^ ^]^ carbene–Au^I^–amide (*k*_RISC_ = 0.8–3.5 × 10^9^ s^−1^),^[^ ^1h^ ^]^ Pd^II^‐TADF (*k*_RISC_ = 2.8–4.7 × 10^9^ s^−1^),^[^ ^13^ ^]^ and Pt^II^‐TADF (*k*_RISC_ = 6.4 × 10^7^ s^−1^)^[^ ^14^ ^]^ complexes, and are orders of magnitude higher than those of organic TADF molecules (*k*_RISC_ = 10^4^–10^6^ s^−1^).^[^ ^15^ ^]^ The small Δ*E*_ST_ (22.2–36.8 meV, close to thermal energy of 25.9 meV at 300 K), together with the enhanced spin‐orbit coupling (SOC) induced by the heavy‐atom effect of Au and S, promotes the endergonic RISC process. Therefore, it is reasonable to infer that these sufficiently large RISC rate constants will facilitate the rapid utilization of triplets via the delayed emission channel, thereby enabling efficient TADF in these pincer gold(III) complexes and providing an opportunity for RISC to occur even at 77 K.

### 2.4 DFT/TDDFT Calculations

**Figure 4:**
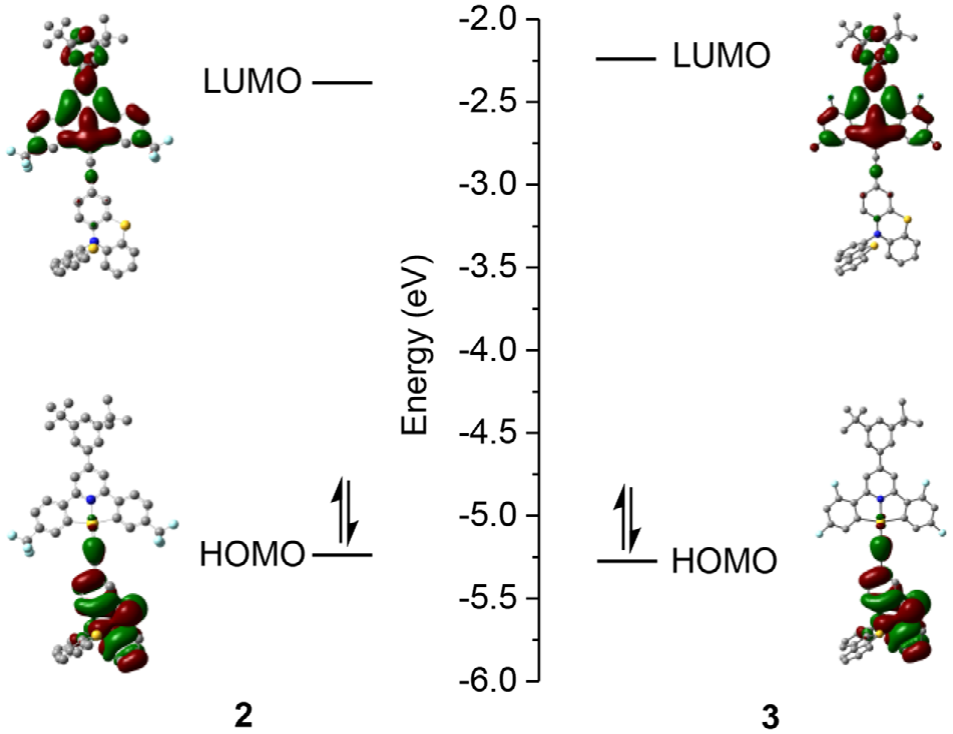
HOMO and LUMO of pincer gold(III) complexes **2** and **3** calculated using semi‐coplanar geometries in the ground states.

Density functional theory (DFT) and time‐dependent density functional theory (TDDFT) calculations were used to study the TADF properties of these gold(III) complexes. The geometry optimization of complexes **2** and **3** in the ground state (S_0_) shows the presence of semi‐coplanar (torsional angle 𝜃_C1‐Au‐C2‐C3_ ∼ 0°), orthogonal (𝜃_C1‐Au‐C2‐C3_ ∼ 90°), and twisted (𝜃_C1‐Au‐C2‐C3_ ∼ 50°) orientation between alkynyl ligand and [C^N^C] ligand (Figure S12, Supporting Information). **Figures** **4** and S13 (Supporting Information) show the frontier molecular orbital diagrams of **2** and **3** calculated using the ground state (S_0_) geometry. The HOMO and LUMO are localized on the alkynyl ligand and [C^N^C] ligand, respectively. HOMO → LUMO excitation gives rise to S_0_ → S_1_^1^LLCT transition. Geometry optimizations of these two complexes in the S_1_ and T_1_ excited states show semi‐coplanar and orthogonal geometries (Figure S12, Supporting Information). The results show that possible torsional dynamics may exist in both the ground state and excited states of these two complexes (considered as a D‐A TADF emitter).

**Figure 5:**
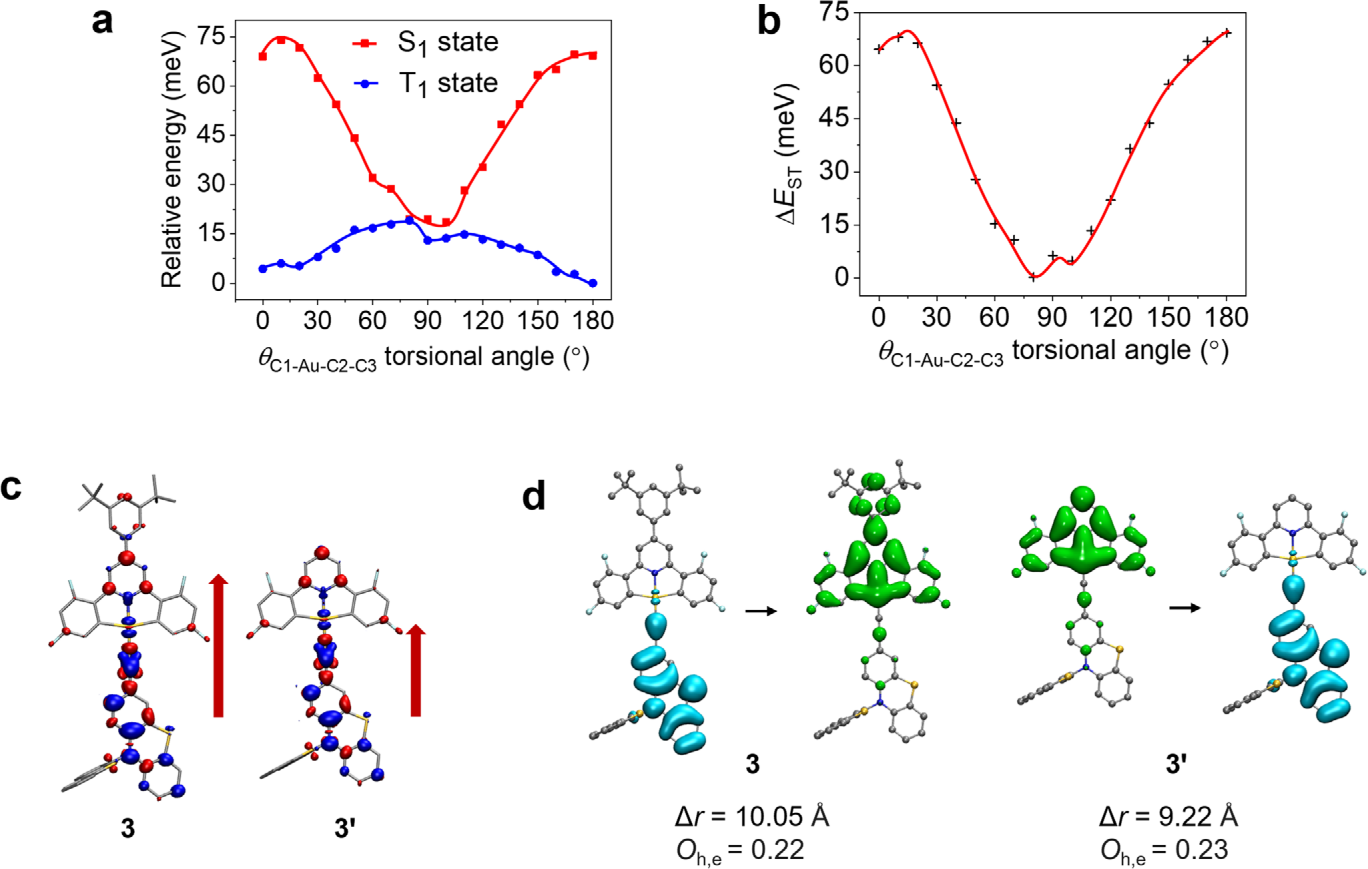
a) Calculated excited state adiabatic potential energy surfaces (relative energy versus torsional angle) of **2**. The relative potential energy was calibrated by adjusting the energy of the coplanar T_1_ structure to 0 eV for comparison. (b) Calculated Δ*E*_ST_ value at different torsional angles (θ_C1‐Au‐C2‐C3_) of **2**. (c) Calculated transition density *t*(r) upon excitation and the direction of transition dipole moment (red arrow) of semi‐coplanar structures of **3** and **3**′. Density increase and decrease are represented by the red and blue isosurfaces, respectively. (d) Calculated hole and electron distribution, the distance between centroids of hole and electron (Δ*r*), and the overlap between hole and electron wavefunctions (*O*_h,e_) of semi‐coplanar structures of **3** and **3**′. Hole and electron distributions are represented by the blue and green isosurfaces, respectively.

To study the effect of excited state torsional dynamics on the TADF properties of the complexes studied here, we performed a restricted geometry optimization of **2** with fixed torsional angles 𝜃_C1‐Au‐C2‐C3_ (0°–180°, step = 10°) and plotted the relationship between its S_1_/T_1_ adiabatic potential energy surface (PES) and the torsional angle (**Figure** **5**a). In the coplanar geometry (𝜃_C1‐Au‐C2‐C3_ = 0°/180°), the Δ*E*_ST_ values are 0.065/0.069 eV. After deformation from coplanar geometry to orthogonal geometry (𝜃_C1‐Au‐C2‐C3_ = 80°), the S_1_ state energy decreases by 0.050/0.050 eV and the T_1_ state energy increases by 0.015/0.019 eV, resulting in a decrease in Δ*E*_ST_ value to 0.0003 eV (Figure 5b). The experimentally estimated Δ*E*_ST_ value of **2** (0.030 eV) falls within the theoretically predicted range (0.0003–0.069 eV). The distortion from coplanar geometry to orthogonal geometry can promote TADF emission by decreasing the Δ*E*_ST_ value and facilitating the RISC. However, the smaller HOMO‐LUMO overlap and reduced transition oscillator strength (*f*_S1_) in the orthogonal geometry suppress the TADF emission.^[^ ^16^ ^]^ The calculated *f*_S1_ values of **2** are 0.137 and 0.004 for coplanar and orthogonal geometries, respectively. By considering the *k*_r_ values of all thermally accessible rotamers on PES, the TADF radiative rate constant (*k*_TADF,avg_) was estimated to be 5.9 × 10^5^ s^−1^, which is comparable to the experimental *k*_r_ value of 1.4 × 10^6^ s^−1^ (for details, please see Table S6, Supporting Information).

To further analyze the effect of π‐extended D‐A geometry on the TADF properties, we performed hole (h^+^) and electron (e^−^) distribution analysis^[^ ^17^, ^18^ ^]^ on **3** and its analogue **3**′. **3**′ is a hypothetical structure in which the 3,5‐di‐*tert*‐butylphenyl group on the *para*‐position of the pyridine ring is omitted (Figure 5c,d). The presence of π‐extended substituent results in a smaller overlap and a longer distance between the hole and electron in **3** compared to **3**′. This results in a smaller Δ*E*_ST_ value for **3** (0.104 eV) than **3**′ (0.127 eV) in semi‐coplanar geometry. The calculated transition density plot is shown in Figure 5c. Compared to **3**′, the π‐extended substituent on **3** provides a more extended transition density and therefore a larger transition oscillator strength (*f*_S1_ = 0.148 for **3** and 0.133 for **3**′ in semi‐coplanar geometry). Therefore, the π‐extended D‐A geometry is beneficial to achieving more efficient TADF.

### 2.5 Electroluminescent Properties

**Figure 6:**
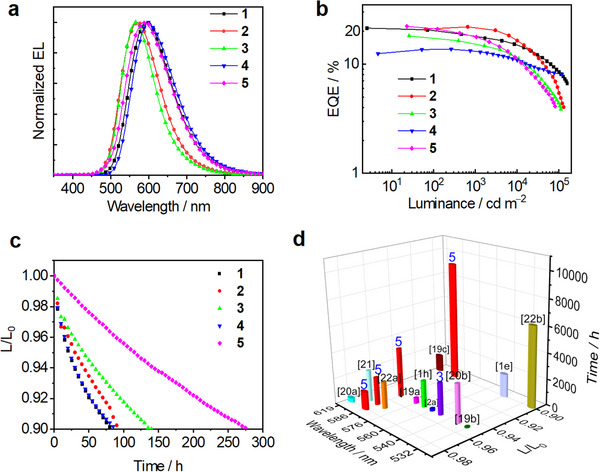
a) Normalized EL spectra and b) EQE‐luminance characteristics of **1** (2 wt%), **2** (2 wt%), **3** (6 wt%), **4** (12 wt%), **5** (2 wt%). c) Relative luminance‐operational lifetime of OLEDs, with L_0_/cd m^−2^ = 10 000 (**1**, 2 wt%), 12 000 (**2**, 2 wt%), 11 000 (**3**, 4 wt%), 6500 (**4**, 12 wt%), 7500 (**5**, 4 wt%). d) Three‐dimensional diagram summarizing the relative luminance‐operational lifetime of OLEDs using TADF emitters as single dopants, Ir^III^‐sensitized hyperphosphorescent emitters, and Pt^II^/Ir^III^‐doped emitters, with *λ*_EL_ in the range of 515–620 nm at L_0_ of 1000 cd m^−2^ (The blue Arabic numerals **3** and **5** denote the emitters investigated in this study, the estimated operational lifetime (LT_90_) at 1000 cd m^−2^ for the reference^[22b]^ is calculated with an acceleration coefficient of 1.7).

To investigate the electroluminescent (EL) performances of these pincer Au^III^‐complexes, which exhibit efficient TADF emission (high PLQYs of up to 90% and short emission lifetimes of <1.2 µs in thin films), vacuum‐deposited OLEDs with device configuration [ITO/FSFA: NDP‐9 (3 wt%) (10 nm)/FSFA (120 nm)/NBP‐BC (5 nm)/Au^III^ emitter: NBP‐BC: PCPF‐Trz (40 nm)/ANT‐Biz: Liq (1:1, 30 nm)/Yb (1 nm)/Ag (100 nm)] were fabricated. We used a 10‐nm‐thick FSFA (N‐([1,1′‐biphenyl]‐2‐yl)‐N‐(9,9‐dimethyl‐9H‐fluoren‐2‐yl)‐9,9′‐spirobi[fluoren]‐2‐amine) doped with 3 wt% of NDP‐9 (2‐(7‐ dicyanomethylene‐1,3,4,5,6,8,9,10‐octafluoro‐7H‐pyrene‐2‐ylidene)‐malononitrile) as the hole‐injecting layer (HIL), 120‐nm‐thick FSFA as the hole‐transporting layer (HTL), 30‐nm‐thick mixture of ANT‐Biz (1‐(4‐(10‐([1,1′‐biphenyl]‐4‐yl)anthracen‐9‐yl)phenyl)‐2‐ethyl‐1H‐benzo[d]imidazole) and Liq with a 1:1 weight ratio as an electron‐transporting layer (ETL) layer, a bilayer of Yb (1nm)/Ag (100 nm) as the cathode. NBP‐BC (9,9′‐bis([1,1′‐biphenyl]‐4‐yl)‐3,3′‐bi‐9H‐carbazole) and PCPF‐Trz (2,4‐diphenyl‐6‐(3′‐(triphenylen‐2‐yl)‐[1,1′‐biphenyl]‐3‐yl)‐1,3,5‐triazine) were used in a 6:4 weight ratio as the co‐host in the emission layer (EML). Furthermore, a 5‐nm‐thick NPB‐BC layer adjacent to the EML was used as an electron‐blocking layer (EBL). **Figure** **6**a,b and Figures S14–S18 (Supporting Information) show the normalized EL spectra and EQE‐luminance characteristics of **1**–**5**. Selected device data are summarized in **Table** **2**. Similar to the PL emission, the EL emission band is broad and devoid of the high‐energy emission typically associated with organic materials, suggesting efficient energy transfer from the organic hosts to the gold(III)‐TADF emitter. The devices based on **1**–**5** showed yellow to orange‐red EL emission (*λ*_EL_: 562–600 nm) with maximum EQE of 21.4% (**1**), 21.9% (**2**), 18.1% (**3**), 13.7% (**4**), and 22.2% (**5**), respectively. A mild efficiency roll‐off (at 1000 cd m^−2^) of 15.4% (**1**), 2.3% (**2**), 13.8% (**3**), 5.1% (**4**), and 19.8% (**5**) was recorded. The fast radiative decay rate constants of these pincer Au^III^‐TADF emitters significantly enhance the radiative decay of excitons, which may contribute to their efficient EL performance.

**Table 2:** Key performances of OLEDs based on **1**–**5** studied in this work.

Emitter [Conc.]	*L*_max_ [cd m^−2^]	CE^a)^ [cd A^−1^]		PE^b)^ [lm W^−1^]		EQE [%]			Efficiency roll‐offs [%]	CIE coordinates [(x, y)]	λ_max_ ^c)^ [nm]
		Max	at 1000 cd m^−2^	Max	at 1000 cd m^−2^	Max	at 1000 cd m^−2^	at 10 000 cd m^−2^	At 1000 cd m^−2^		
**1** (2 wt%)	156 000	39.6	36.6	62.0	34.9	21.4	18.1	15.1	15.4	0.53, 0.47	596
**2** (2 wt%)	130 000	60.0	58.5	71.4	58.8	21.9	21.4	16.5	2.3	0.46, 0.51	570
**3** (6 wt%)	112 000	51.1	45.4	64.2	44.7	18.1	15.6	11.3	13.8	0.46, 0.52	566
**4** (12 wt%)	118 000	23.9	23.5	26.1	17.6	13.7	13.0	10.9	5.1	0.55, 0.45	599
**5** (2 wt%)	81 000	43.8	37.9	45.8	26.5	22.2	17.8	11.2	19.8	0.50, 0.48	584

^a)^ Current efficiency;

^b)^ power efficiency;

^c)^ emission maximum.

The operational stability of OLEDs based on **1**–**5** was then evaluated under our laboratory conditions, with detailed information on the device architectures and materials provided in the Supporting Information. As shown in Figure 6c and Table S 7(Supporting Information), the device exhibited an impressive LT_95_ (luminance drops to 95% of an initial luminance (L_0_)) of 21.1 h (**1**) at L_0_ of 10 000 cd m^−2^, 30.2 h (**2**) at 12 000 cd m^−2^, 41.9 h (**3**) at 11 000 cd m^−2^, 22.3 h (**4**) at 6500 cd m^−2^, and 115 h (**5**) at 7500 cd m^−2^, respectively. Accordingly, using the acceleration coefficients (*n* = 1.7 for emitters **1**–**4**, *n* = 1.74 for emitter **5**), the LT_95_ values at L_0_ of 1000 cd m^−2^ were estimated to be 1056 h (**1**), 2064 h (**2**), 2472 h (**3**), 537 h (**4**), and 3831 h (**5**). (Figure S20, Supporting Information). It is noteworthy that the estimated operational lifetime of the device based on complex **5** (LT_98_ 1399 h, LT_97_ 2169 h, LT_95_ 3831 h, and LT_90_ 9261 h at L_0_ of 1000 cd m^−2^) represents a significant advancement for gold OLEDs, achieving one of the record‐high values among the non‐iridium OLEDs.^[^ ^1^, ^2^, ^9^, ^19^ ^]^ Moreover, this durability is comparable to some high‐performance Ir^III^‐sensitized hyper‐OLEDs,^[^ ^20^ ^]^ and some Pt^II^/Ir^III^‐doped OLEDs^[^ ^20^, ^21^, ^22^ ^]^ without utilizing device structure optimization techniques (Figure 6d). The excellent operational stability can be attributed to several factors: i) The small Δ*E*_ST_ and ultrafast RISC of these pincer Au^III^ emitters are believed to effectively deplete the triplet excited states, thereby mitigating triplet‐related annihilation processes at high luminance;^[^ ^23^ ^]^ ii) the introduction of a phenyl group at the *para*‐position of the pyridine ring in the complex is conceived to strengthen the metal‐N bond and block the *para*‐reactive site of the pyridine ring, which has a positive impact on the device operational stability;^[^ ^2^, ^5^, ^24^ ^]^ iii) the satisfactory electrochemical stability of these pincer gold(III) complexes is beneficial to mitigating device degradations. In addition, the deuterium kinetic isotope effects (KIEs) may also play a role in enhancing the device performance and durability of complex **5**.^[^ ^25^ ^]^

## 3 Conclusion

In summary, we developed a new set of stable pincer gold(III)‐TADF emitters characterized by an extended π‐distance along the transition dipole moment. Such emitters exhibit small Δ*E*_ST_ (22.2–36.8 meV) and efficient room‐temperature spin‐flipping RISC (*k*_RISC_: 0.88–2.9 × 10^10^ s^−1^). As a result, we achieved efficient TADF with PLQYs as high as 90%, excited‐state lifetimes of <1.2 µs, and *k*_r_ values reaching 10^6^ s^−1^. Vacuum‐deposited OLEDs utilizing these pincer gold(III)‐TADF emitters exhibited significantly extended operational lifetimes of up to 3831 h at a practical luminance of 1000 cd m^−2^, achieving high EQE_max_ values of up to 22.2%, and relieved efficiency roll‐off of down to 2.3% at 1000 cd m^−2^. The OLED devices based on the pincer gold(III)‐TADF emitter in this study exhibited outstanding device performance and exceptionally extended operational lifetime, providing new inspiration for the development of practical OLEDs utilizing innovative pincer metal emitters.

## Conflict of Interest

The authors declare no conflict of interest.

## Supporting information



Supporting Information

## Data Availability

The data that support the findings of this study are available on request from the corresponding author. The data are not publicly available due to privacy or ethical restrictions.
