# Supplementary material for: Stable Pincer Gold(III)‐TADF Emitters with Extended Donor–Acceptor Separation for Efficient Vacuum‐Deposited OLEDs with Operational Lifetime (LT95) up to 3831 h at 1000 cd m−2
Source: Adv Sci (Weinh). 2025 Apr 26;12(27):2502529. doi: 10.1002/advs.202502529 (PMC12279227; doi:10.1002/advs.202502529)
Supplement: Supplementary file 1 — Supporting Information [file ADVS-12-2502529-s001.docx]

Supporting Information

**Stable Pincer Gold(III)-TADF Emitters with Extended Donor–Acceptor Separation for Efficient Vacuum-Deposited OLEDs with Operational Lifetime (LT_95_) up to 3831 h at 1000 cd m^–2^**

*Hui-Xing Shu, Shuo Xu, Wai-Pong To, Gang Cheng,* and Chi-Ming Che**

**Table of Contents**

[General Experimental Section 3](#_Toc185625007)

[Synthesis and Characterization 4](#_Toc185625008)

[Thermogravimetric analysis (TGA) 8](#_Toc185625009)

[X-ray crystallography 9](#_Toc185625010)

[Cyclic voltammetry (CV) 11](#_Toc185625011)

[Variable-temperature emission lifetime measurement 15](#_Toc185625012)

[Time-resolved Transient Absorption Spectroscopy 16](#_Toc185625013)

[Computational details 19](#_Toc185625014)

[Electroluminescence 22](#_Toc185625015)

[References 32](#_Toc185625016)

[NMR spectra 34](#_Toc185625017)

[Cartesian coordinates for DFT optimized structures 43](#_Toc185625018)

General Experimental Section

All chemicals, unless otherwise noted, were purchased from commercial sources and were used without further purification. All solvents for reactions and measurement were purified by standard method. NMR spectra were recorded at 600, 500 or 400 MHz for ^1^H NMR and 151, 126 or 101 MHz for ^13^C NMR. All chemical shifts (δ) were reported in ppm; the chemical shifts were calibrated with the corresponding solvent residual peaks. High-resolution electrospray ionization (HR-ESI) mass spectra were recorded on Waters Micromass Q-Tof Premier or Bruker maXis II high resolution Q-Tof mass spectrometer.

Thermogravimetric analyses (TGAs) were conducted on a TA Instrument Q50(TGA) thermogravimeter by measuring the weight loss while heating the sample at a rate of 10 °C/min on Al_2_O_3_ pan purged by N_2_ with flow rate of 50 mL/min.

Cyclic voltammetric and differential pulse voltammetric measurements were performed on CH Instruments Electrochemical Analyzer CHI620E using a three-compartment electrochemical cell, with samples in 0.1 M [*n*Bu_4_N]PF_6_ as supporting electrolyte in DMF. The solutions were degassed with argon prior to measurements. Saturated calomel electrode (SCE), glassy carbon, and platinum wire were used as the reference electrode, working electrode, and counter electrode, respectively. All measurements were conducted at room temperature. The ferrocenium/ferrocene (Cp_2_Fe^+/0^) couple was used as the internal standard.

Single crystal structure was determined using the low temperature (100 K) single-crystals X-ray diffraction, performed on a Bruker D8 VENTURE diffractometer, and solved with Olex2 software. Supplementary crystallographic data can be accessed free of charge from the Cambridge Crystallographic Data Centre at www.ccdc.cam.ac.uk/data_request/cif (CCDC 2407652).

All absorption spectra were recorded on a Hewlett-Packard 8453 diode array spectrophotometer. Steady-state emission spectra were recorded on a Horiba Fluorolog-3 spectrophotometer. Solutions for photophysical studies were degassed by using a high vacuum line in a two-compartment cell with five freeze-pump-thaw cycles. Low temperature (77 K) emission spectra for glassy state and solid state samples were recorded in quartz tubes (4 mm internal diameter) placed in a liquid nitrogen Dewar flask with quartz windows. Emission quantum yields were measured with Hamamatsu C11347 Quantaurus-QY Absolute PL quantum yields measurement system. Nanosecond time-resolved emission measurements were performed on a LP920-KS Laser Flash Photolysis Spectrometer (Edinburgh Instruments Ltd., Livingston, UK). The excitation source was the 355 nm, 266 nm output (third harmonic) of a Nd:YAG laser (Spectra-Physics Quanta-Ray Lab-130 Pulsed Nd:YAG Laser). The signals were processed by a PC plug-in controller with L900 software. The preparation of samples for the measurements was the same as those for steady-state emission measurements. Femtosecond time-resolved transient absorption (fs-TA) measurements were performed based on a commercial Ti:Sapphire regenerative amplifier laser system (800 nm, 120 fs, 1 kHz, and 3.5 mJ/pulse). In the fs-TA, the samples were probed by a white light continuum pulse created from sapphire pumped by the 800 nm laser. The temporal delay of probe to pump pulse was varied by a computer controlled optical delay line. The fs-TA signals were collected by a monochromator and detected with an air-cooled CCD detector. The instrument response function (IRF) for the fs-TA is ~200–400 fs varying slightly with the spectral wavelength. Femtosecond time-resolved fluorescence (fs-TRF) measurements were performed on the same setup as fs-TA. The output 800 nm laser pulse (200 mW) was used as gate pulse while the 400 nm laser pulse (10 mW) (second harmonic) was used as the pump laser. After excitation by the pump laser, the sample fluorescence was focused into the nonlinear crystal (BBO) mixing with the gate pulse to generate the sum frequency signal. Broadband fluorescence spectra were obtained by changing the crystal angles and the spectra were detected by the air-cooled CCD.

Synthesis and Characterization

**Ligand synthesis**

**
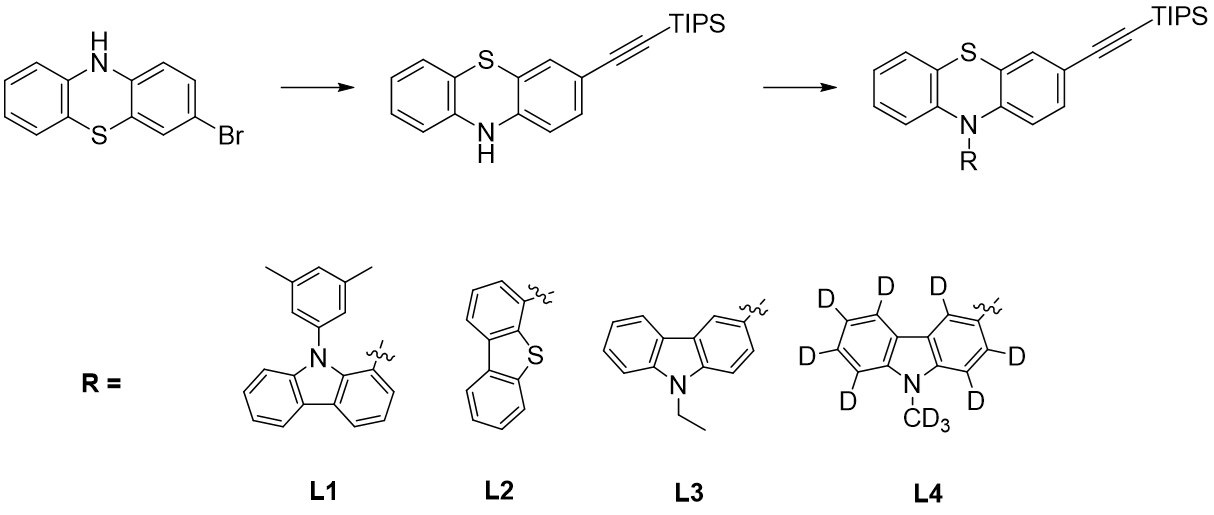
**

**Scheme S1**. Synthesis of the triisopropylsilyl-protected aryl-ethynyl ligands **L1**–**L4**.

**Synthesis of L1:** To a solution of (triisopropylsilyl)acetylene aryl compound (500‒600 mg, 1 equiv.) and 1-bromo-9-(3,5-dimethylphenyl)-9*H*-carbazole (1.1 equiv.) in Toluene (10.0 mL) was added Pd(dba)_2_ (0.02 equiv.), *t*Bu_3_PHBF_4_ (0.04 equiv.) and NaO*t*Bu (2.0 equiv.). The resulting mixture was bubbled with argon for 10 min, and then stirred at 100 ̊C overnight. After that, the reaction solution was diluted with Et_2_O (50.0 mL), washed with water (50.0 mL), brine (50.0 mL) and dried over Na_2_SO_4_. The solvent was removed under reduced pressure and the residue was purified by column chromatography over silica gel to give **L1**.

**L1**: Yield: 197 mg (78%, off-white solid). ^1^H NMR (500 MHz, CDCl_3_) δ 8.27 (dd, *J* = 7.8, 1.1 Hz, 1H), 8.16 (dt, *J* = 7.8, 1.0 Hz, 1H), 7.43 (t, *J* = 7.7 Hz, 1H), 7.36 (ddd, *J* = 8.2, 7.1, 1.3 Hz, 1H), 7.33 – 7.24 (m, 2H), 7.07 (s, 1H), 7.03 – 6.93 (m, 1H), 6.67 (dd, *J* = 8.5, 2.0 Hz, 2H), 6.62 – 6.55 (m, 3H), 6.53 – 6.48 (m, 2H), 5.87 – 5.76 (m, 1H), 5.67 (d, *J* = 8.5 Hz, 1H), 2.08 (s, 3H), 2.01 (s, 3H), 1.08 (s, 21H); ^13^C NMR (126 MHz, CDCl_3_) δ 143.02, 141.65, 141.19, 138.71, 137.76, 137.03, 130.64, 130.40, 129.55, 128.67, 127.23, 126.67, 126.57, 126.37, 126.34, 125.66, 122.75, 122.09, 121.12, 121.11, 120.16, 119.99, 117.08, 116.60, 115.65, 114.95, 110.67, 106.72, 89.65, 21.27, 21.13, 18.79, 11.46. HRMS (+ESI): m/z Calcd. for C_43_H_45_N_2_SSi [M+H^+^]: 649.3067, found: 649.3043.

**Synthesis of L2:** The procedure is similar to that for synthesizing **L1**, starting from 4-iododibenzo[*b*,*d*]thiophene instead of 1-bromo-9-(3,5-dimethylphenyl)-9*H*-carbazole. Yield: 594 mg (85%, off-white solid). ^1^H NMR (400 MHz, CDCl_3_) δ 8.29 (d, *J* = 7.8 Hz, 1H), 8.21 (dd, *J* = 7.5, 1.7 Hz, 1H), 7.82 – 7.74 (m, 1H), 7.70 (t, *J* = 7.7 Hz, 1H), 7.57 (d, *J* = 7.5 Hz, 1H), 7.48 (tt, *J* = 8.8, 6.5 Hz, 2H), 7.16 (d, *J* = 1.9 Hz, 1H), 7.03 (dt, *J* = 7.4, 1.5 Hz, 1H), 6.88 (dd, *J* = 8.5, 1.9 Hz, 1H), 6.86 – 6.73 (m, 2H), 6.19 (dd, *J* = 8.0, 1.5 Hz, 1H), 6.08 (d, *J* = 8.5 Hz, 1H), 1.10 (s, 21H); ^13^C NMR (101 MHz, CDCl_3_) δ 142.45, 141.93, 140.66, 139.65, 138.79, 135.88, 134.88, 131.08, 130.13, 129.58, 127.59, 127.22, 126.94, 126.49, 124.83, 123.32, 123.21, 122.00, 121.92, 120.19, 119.81, 118.01, 115.60, 114.97, 106.42, 90.37, 18.79, 11.44. HRMS (+ESI): m/z Calcd. for C_35_H_36_NS_2_Si [M+H^+^]: 562.2053, found: 562.2057.

**Synthesis of L3**: The procedure is similar to that for synthesizing **L1**, starting from 3-bromo-9-methyl-9*H*-carbazole instead of 1-bromo-9-(3,5-dimethylphenyl)-9*H*-carbazole. Yield: 590 mg (81%, off-white solid). ^1^H NMR (500 MHz, CD_2_Cl_2_) δ 8.10 (d, *J* = 2.0 Hz, 1H), 8.07 (d, *J* = 7.8 Hz, 1H), 7.66 (d, *J* = 8.5 Hz, 1H), 7.57 – 7.46 (m, 2H), 7.42 (dd, *J* = 8.5, 2.1 Hz, 1H), 7.26 (ddd, *J* = 8.0, 6.4, 1.7 Hz, 1H), 7.11 (d, *J* = 1.9 Hz, 1H), 7.00 – 6.93 (m, 1H), 6.88 (dd, *J* = 8.5, 2.0 Hz, 1H), 6.82 – 6.71 (m, 2H), 6.25 – 6.14 (m, 1H), 6.13 (s, 1H), 4.46 (q, *J* = 7.2 Hz, 2H), 1.51 (t, *J* = 7.2 Hz, 3H), 1.11 (s, 21H); ^13^C NMR (126 MHz, CD_2_Cl_2_) δ 145.69, 145.00, 141.11, 139.96, 131.84, 131.18, 130.13, 128.54, 127.49, 126.97, 126.95, 125.24, 123.31, 123.08, 121.17, 119.88, 119.85, 119.45, 117.46, 116.74, 116.00, 111.21, 109.55, 107.02, 90.48, 38.44, 19.00, 14.23, 11.92. HRMS (+ESI): m/z Calcd. for C_37_H_41_N_2_SSi [M+H^+^]: 573.2754, found: 573.2744.

**Synthesis of L4**: The procedure is similar to that for synthesizing **L1**, starting from 3-bromo-9-(methyl-*d*_3_)-9*H*-carbazole-1,2,4,5,6,7,8-*d*_7_ instead of 1-bromo-9-(3,5-dimethylphenyl)-9*H*-carbazole. Yield: 590 mg (83%, off-white solid). ^1^H NMR (500 MHz, CD_2_Cl_2_) δ 7.10 (d, J = 1.9 Hz, 1H), 7.01 – 6.95 (m, 1H), 6.87 (dd, *J* = 8.6, 2.0 Hz, 1H), 6.82 – 6.74 (m, 2H), 6.22 – 6.16 (m, 1H), 6.11 (d, *J* = 8.6 Hz, 1H), 1.12 (s, 21H); ^13^C NMR (126 MHz, CD_2_Cl_2_) δ 145.69, 145.00, 142.16, 140.99, 131.74, 131.17, 130.13, 127.48, 126.96, 124.97, 123.07, 122.82, 119.86, 119.46, 117.45, 116.70, 115.96, 106.99, 90.47, 18.99, 11.91. HRMS (+ESI): m/z Calcd. for C_36_H_29_D_10_N_2_SSi [M+H^+^]: 569.3225, found: 569.3208.

**Synthesis of complexes**

**Chart S1.** Chemical structures of complexes **1**–**5**.

**Scheme S2.** Synthesis of complexes **1**–**5**.

**General procedures for the synthesis of complexes 1–5**

A mixture of [Au(C^N^C)Cl] (60‒150 mg, 1 equiv.) and triisopropylsilyl-protected aryl-ethynyl ligands (1.01 equiv.) was reacted in CH_2_Cl_2_ solution with a catalytic amount of CuI in the presence of triethylamine (1.5 equiv.) and TBAF (tetra-*n*-butylammonium fluoride) (1.5 equiv.), at room temperature for 12 h under argon.

For **1–4**: The solvent was removed under reduced pressure and the residue was purified by column chromatography over silica gel.

For **5**: After concentrating the solvent to ~ 2.0 mL, the crude product was precipitated out upon adding 10.0 mL ethanol, the mixture was filtrated and the filter cake was washed with ethanol (2 × 5.0 mL) and diethyl ether (2 × 5.0 mL). Further purification was conducted by recrystallization with Et_2_O/CH_2_Cl_2_.

**1**: Yield: 100 mg (78%, orange solid). ^1^H NMR (600 MHz, CD_2_Cl_2_) δ 8.33 (dd, *J* = 7.8, 1.1 Hz, 1H), 8.20 (dt, *J* = 7.9, 1.0 Hz, 1H), 8.12 – 8.04 (m, 2H), 7.76 (s, 2H), 7.69 (d, *J* = 8.0 Hz, 2H), 7.58 (t, *J* = 1.7 Hz, 1H), 7.54 (d, *J* = 1.8 Hz, 2H), 7.49 (t, *J* = 7.7 Hz, 1H), 7.44 (dd, *J* = 8.2, 1.9 Hz, 2H), 7.37 (ddd, *J* = 8.2, 7.1, 1.2 Hz, 1H), 7.34 – 7.25 (m, 2H), 7.17 – 7.07 (m, 1H), 6.95 (dt, *J* = 8.3, 0.9 Hz, 1H), 6.65 – 6.57 (m, 4H), 6.56 – 6.49 (m, 2H), 6.40 (dd, *J* = 8.4, 2.0 Hz, 1H), 5.80 (dt, *J* = 7.8, 1.1 Hz, 1H), 5.64 (d, *J* = 8.5 Hz, 1H), 2.12 (s, 3H), 2.03 (s, 3H), 1.36 (s, 18H); ^13^C NMR (151 MHz, CD_2_Cl_2_) δ 166.56, 163.54, 157.99, 152.99, 152.92, 143.49, 141.79, 141.12, 139.25, 139.13, 138.39, 137.50, 137.27, 132.97, 132.74, 132.53, 131.04, 130.23, 130.13, 128.72, 127.68, 126.99, 126.87, 126.76, 126.67, 126.00, 125.77, 125.67, 124.31, 124.29, 123.97, 123.37, 123.27, 122.28, 122.27, 121.53, 121.42, 120.48, 120.36, 119.56, 118.10, 117.60, 117.46, 115.93, 115.55, 110.93, 100.62, 89.55, 35.56, 31.67, 21.43, 21.29. HRMS (+ESI): m/z Calcd. for C_67_H_53_AuF_6_N_3_S [M+H^+^]: 1242.3525, found: 1242.3493. Elemental Anal. (%) Calcd for C_67_H_52_AuF_6_N_3_S•2.5THF: C 65.02, H 5.1, N 2.95; found: C 65.18, H 5.15, N 2.88.

**2**: Yield: 105 mg (75%, yellow solid). ^1^H NMR (500 MHz, CDCl_3_) δ 8.32 (dd, *J* = 7.9, 1.1 Hz, 1H), 8.27 – 8.22 (m, 1H), 8.10 (s, 2H), 7.84 – 7.78 (m, 1H), 7.74 (t, *J* = 7.7 Hz, 1H), 7.66 (s, 2H), 7.60 (dd, *J* = 7.6, 1.0 Hz, 1H), 7.57 (d, *J* = 8.1 Hz, 2H), 7.54 (q, *J* = 3.6, 2.6 Hz, 1H), 7.52 – 7.46 (m, 4H), 7.36 (d, *J* = 8.1 Hz, 2H), 7.08 – 7.03 (m, 2H), 6.87 – 6.75 (m, 2H), 6.56 (d, *J* = 8.4 Hz, 1H), 6.20 (dd, *J* = 8.1, 1.4 Hz, 1H), 6.01 (d, *J* = 8.5 Hz, 1H), 1.34 (s, 18H); ^13^C NMR (151 MHz, CDCl_3_) δ 166.13, 163.09, 157.55, 152.44, 152.17, 142.24, 141.37, 140.89, 139.78, 138.84, 137.05, 135.99, 135.18, 132.64, 132.19, 130.57, 129.71, 127.57, 127.06, 126.91, 126.43, 125.21, 125.12, 125.03, 124.80, 123.78, 123.40, 123.24, 123.07, 121.99, 121.85, 121.81, 120.54, 120.25, 119.88, 117.60, 115.47, 114.99, 100.06, 89.74, 35.20, 31.49. HRMS (+ESI): m/z Calcd. for C_59_H_44_AuF_6_N_2_S_2_ [M+H^+^]: 1155.2510, found: 1155.2492. Elemental Anal. (%) Calcd for C_59_H_43_AuF_6_N_2_S_2_: C 61.35, H 3.75, N 2.43; found: C 61.58, H 3.87, N 2.36.

**3**: Yield: 106 mg (75%, yellow solid). ^1^H NMR (600 MHz, CD_2_Cl_2_) δ 8.35 (dd, *J* = 7.9, 1.0 Hz, 1H), 8.27 (dd, *J* = 7.3, 1.6 Hz, 1H), 7.95 (s, 2H), 7.88 – 7.80 (m, 1H), 7.76 (t, *J* = 7.7 Hz, 1H), 7.64 (d, *J* = 7.5 Hz, 1H), 7.57 (d, *J* = 1.8 Hz, 1H), 7.55 – 7.46 (m, 4H), 7.42 (dd, *J* = 6.5, 2.5 Hz, 2H), 7.13 (s, 1H), 7.07 (dd, *J* = 7.4, 1.7 Hz, 1H), 6.80 (dt, *J* = 23.4, 13.1 Hz, 3H), 6.65 (ddd, *J* = 11.5, 8.6, 2.5 Hz, 2H), 6.23 (d, *J* = 8.2 Hz, 1H), 6.11 (d, *J* = 8.5 Hz, 1H), 1.35 (s, 18H); ^13^C NMR (151 MHz, CD_2_Cl_2_) δ 167.59, 165.60, 165.53, 163.87, 163.81, 162.54, 162.47, 160.93, 160.79, 160.72, 157.54, 152.23, 142.22, 141.55, 139.52, 138.85, 137.01, 135.97, 132.42, 130.63, 129.84, 129.58, 127.58, 127.15, 126.88, 126.56, 125.00, 124.87, 123.17, 122.02, 121.93, 121.85, 120.04, 119.45, 119.33, 119.00, 118.88, 115.57, 115.11, 103.29, 103.11, 102.94, 99.67, 90.50, 53.94, 35.04, 31.21. HRMS (+ESI): m/z Calcd. for C_57_H_41_AuF_4_N_2_S_2_ [M]^+^: 1090. 2307, found: 1090. 2342. Elemental Anal. (%) Calcd for C_57_H_41_AuF_4_N_2_S_2_: C 62.75, H 3.79, N 2.57; found: C 62.56, H 3.68, N 2.66.

**4**: Yield: 108 mg (75%, yellow solid). ^1^H NMR (500 MHz, CDCl_3_) δ 8.16 (d, *J* = 1.9 Hz, 2H), 8.13 (d, *J* = 2.0 Hz, 1H), 8.10 (dt, *J* = 7.8, 1.0 Hz, 1H), 7.68 (s, 2H), 7.65 (d, *J* = 8.5 Hz, 1H), 7.62 (d, *J* = 8.1 Hz, 2H), 7.56 – 7.52 (m, 2H), 7.51 (s, 1H), 7.49 (d, *J* = 1.8 Hz, 2H), 7.45 (dd, *J* = 8.5, 2.0 Hz, 1H), 7.42 – 7.38 (m, 2H), 7.31 – 7.27 (m, 1H), 7.06 (d, *J* = 1.9 Hz, 1H), 7.00 (dd, *J* = 5.8, 3.3 Hz, 1H), 6.82 – 6.75 (m, 2H), 6.68 (dd, *J* = 8.5, 1.9 Hz, 1H), 6.24 – 6.18 (m, 1H), 6.04 (d, *J* = 8.5 Hz, 1H), 4.48 (q, *J* = 7.2 Hz, 2H), 1.56 (t, *J* = 6.6 Hz, 3H), 1.37 (s, 18H); ^13^C NMR (151 MHz, CDCl_3_) δ 166.29, 163.30, 157.65, 152.47, 152.21, 144.84, 144.17, 140.65, 139.43, 137.03, 132.97, 132.71, 132.50, 131.73, 130.62, 129.53, 128.43, 126.86, 126.64, 126.52, 125.21, 125.12, 124.87, 123.88, 123.40, 123.20, 122.77, 122.35, 121.80, 120.94, 119.65, 119.53, 119.41, 119.09, 117.46, 116.02, 115.59, 110.56, 108.97, 100.54, 89.23, 38.01, 35.26, 31.54, 14.14. HRMS (+ESI): m/z Calcd. for C_61_H_49_AuF_6_N_3_S [M+H^+^]: 1166.3212, found: 1166.3184. Elemental Anal. (%) Calcd for C_61_H_48_AuF_6_N_3_S•1.5THF•0.5CHCl_3_: C 60.78, H 4.57, N 3.16; found: C 59.56, H 4.79, N 3.17.

**5**: Yield: 95 mg (73%, red solid). ^1^H NMR (500 MHz, CDCl_3_) δ 8.29 (s, 2H), 7.83 – 7.66 (m, 4H), 7.62 (d, *J* = 1.7 Hz, 1H), 7.55 – 7.48 (m, 4H), 7.20 (d, *J* = 2.0 Hz, 1H), 7.04 – 6.99 (m, 1H), 6.93 (d, *J* = 9.3 Hz, 1H), 6.86 – 6.73 (m, 2H), 6.23 – 6.16 (m, 1H), 6.11 (d, *J* = 8.5 Hz, 1H), 1.41 (s, 18H). The ^13^C NMR was not recorded due to low solubility. HRMS (+ESI): m/z Calcd. for C_60_H_36_D_10_AuF_6_N_3_S [M]^+^: 1161.3605, found: 1161.3582.

Thermogravimetric analysis (TGA)

**Figure S1.** TGA thermograms of **1**–**5** studied in this work.

**Table S1.** Thermal decomposition temperature of **1**–**5**.

| Complex | T_d_ (°C) (5 wt.% loss) |
| --- | --- |
| **1** | 378 |
| **2** | 394 |
| **3** | 386 |
| **4** | 404 |
| **5** | 386 |

X-ray crystallography

| **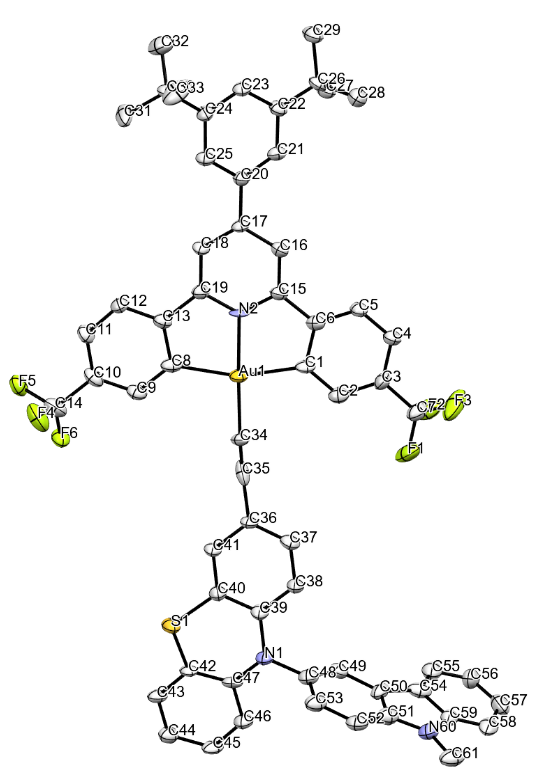** | 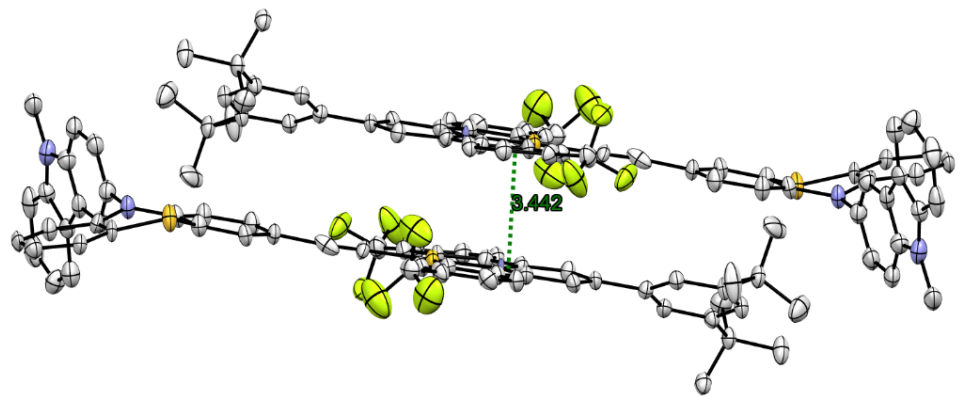 |
| --- | --- |

**Figure S2.** ORTEP drawing for **5** with the omission of hydrogen atoms (ellipsoid probability level: 30%).

**Table S2**. Crystallographic data for **5**

| Compound | **5** |
| --- | --- |
| Formula | C_60_H_36_AuD_10_F_6_N_3_S |
| Formula weight | 1161.36 |
| Crystal system | Triclinic |
| Space group | *P* |
| *a* [Å] | 9.5127 (5) |
| *b* [Å] | 13.7009 (6) |
| *c* [Å] | 20.8704 (10) |
| *α* [°] | 72.226 (3) |
| *β* [°] | 86.507 (3) |
| *γ* [°] | 78.900 (3) |
| *V* [Å^3^] | 2541.8 (2) |
| *ρ* [g cm^–3^] | 1.516 |
| *Z* | 2 |
| Radiation | Ga *K*α (λ = 1.34138 Å) |
| *μ* (mm^–1^) | 4.33 |
| *T*_min_, *T*_max_ | 0.615, 0.751 |
| *F*(000) | 1152 |
| *T* [K] | 100 |
| (sin *θ*/*λ*)_max_ (Å^–1^) | 0.596 |
| *R*_int_ [%] | 11.9 |
| *R*[*F*^2^ > 2*σ*(*F*^2^)] [%] | 6.9 |
| *wR(F*^2^*)* [%] | 19.4 |
| Goodness of fit | 1.01 |
| CCDC number | 2407652 |

**Table S3.** Selected bond lengths and angles of **5**.

| Selected bond lengths (Å) | | | |
| --- | --- | --- | --- |
| Au1—N2 | 1.986 (6) | Au1—C8 | 2.077 (11) |
| Au1—C34 | 2.006 (9) |  |  |
| Au1—C1 | 2.068 (10) |  |  |
| Selected bond angles (º) | | | |
| N2—Au1—C34 | 177.9 (4) | C15—N2—Au1 | 118.1 (6) |
| N2—Au1—C1 | 81.2 (3) | C19—N2—Au1 | 118.8 (6) |
| N2—Au1—C8 | 81.2 (3) | C35—C34—Au1 | 177.0 (10) |
| C34—Au1—C1 | 96.8 (4) | C6—C1—Au1 | 111.1 (7) |
| C34—Au1—C8 | 100.7 (4) | C2—C1—Au1 | 130.2 (7) |
| C1—Au1—C8 | 162.4 (3) |  |  |

Cyclic voltammetry (CV)


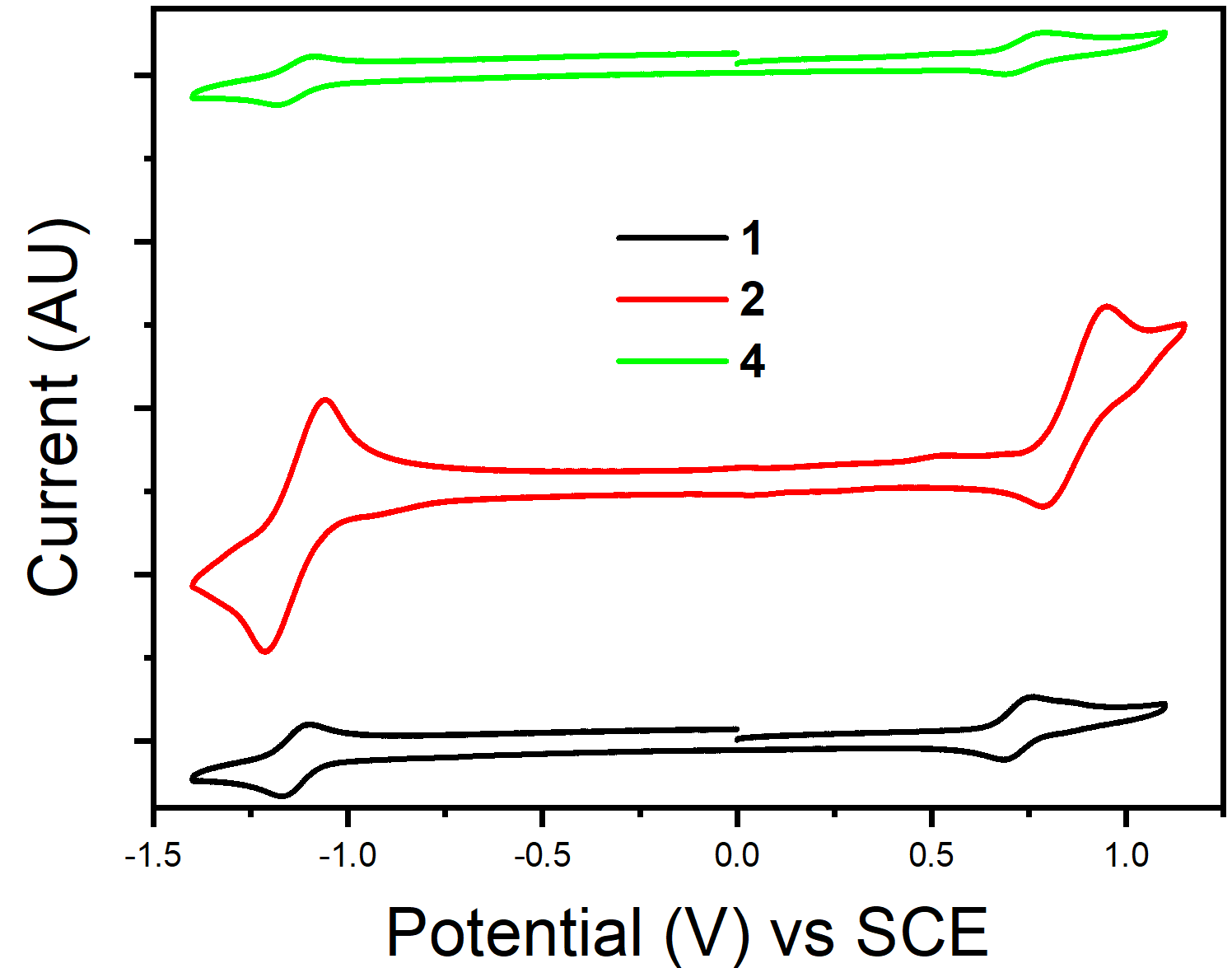

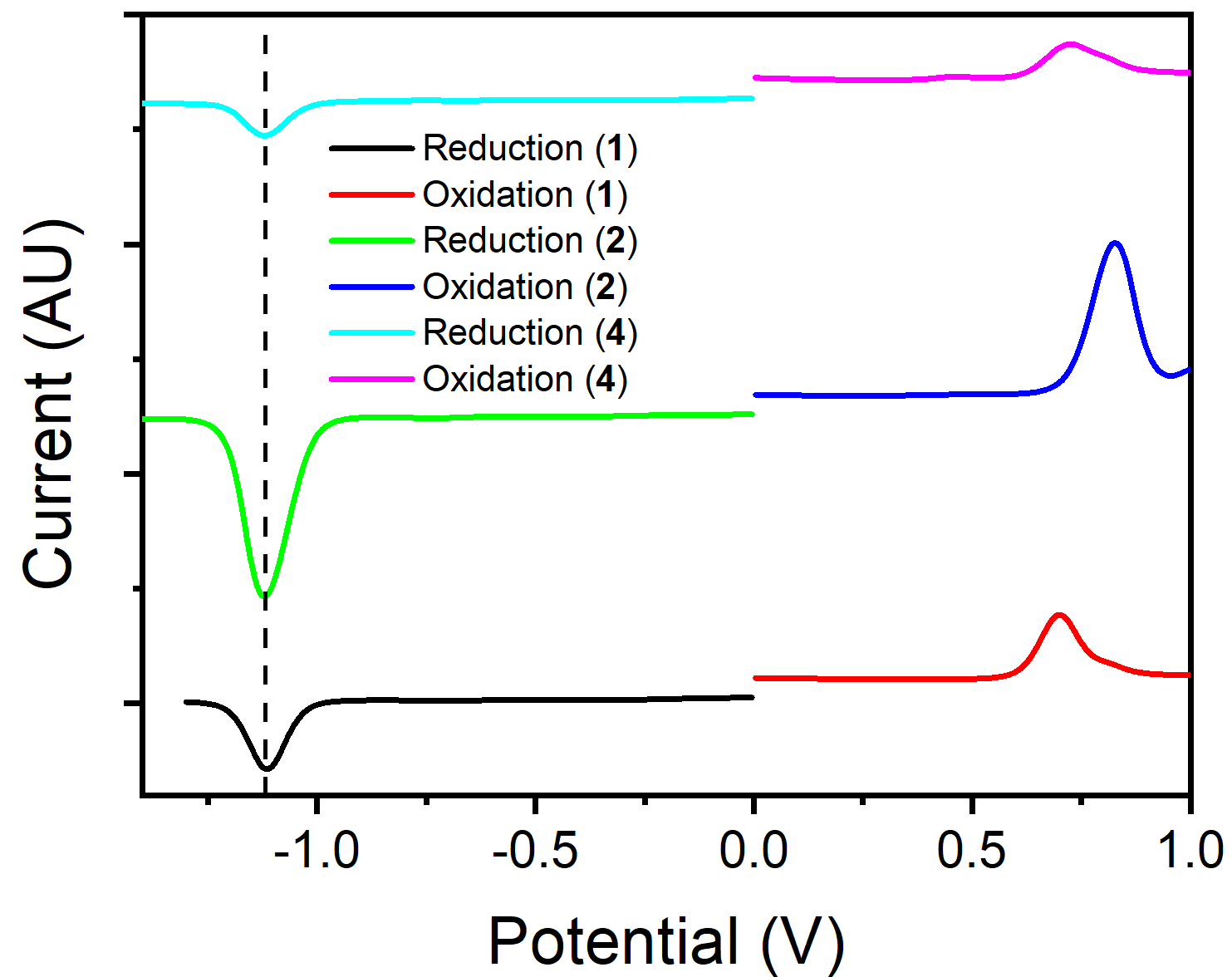


**Figure S3.** Cyclic voltammograms (upper) and DPV (bottom) curves of **1**, **2**, and **4** recorded in DMF with [*n*Bu_4_N]PF_6_ (0.1 M) as supporting electrolyte at a scan rate of 100 mV/s (potential values vs. SCE); Fc^+^/Fc was used as internal reference, with E_1/2_(Fc^+^/Fc) values ranging from 0.49 to 0.51 V vs. SCE.

**Table S4.** Summary of electrochemical properties of **1**, **2**, and **4**.

| Emitter | E_ox_ / V  [V] | E_red_  [V] | HOMO  [eV] | LUMO  [eV] | Δ*E*  [eV] |
| --- | --- | --- | --- | --- | --- |
|  |  |  |  |  |  |
| **1** | 0.70 | –1.12 | –5.60 | –3.51 | 2.09 |
| **2** | 0.83 | –1.12 | –5.74 | –3.51 | 2.23 |
| **4** | 0.73 | –1.12 | –5.63 | –3.51 | 2.12 |

E_ox_ and E_red_ were estimated with DPV or onset value; HOMO = –(1.15 × E_ox_ + 4.79), LUMO = 1.18 × (–E_red_) − 4.83;^1^ Δ*E* is the HOMO-LUMO gap.

**Photophysical studies**

**Table S5.** Photophysical data of **1**–**5**.

| Emitter | Medium | *λ*_abs_/nm (*ε*×10^3^ [dm^3^ mol^–1^ cm^–1^]) | *λ*_em_/nm (*τ*/µs) | *φ* (%) | *k*_r_×10^5^/s^–1^ |
| --- | --- | --- | --- | --- | --- |
| **1** | Toluene | 295 (48.3), 310 (31.2), 321 (sh, 28.8), 375 (sh, 9.1), 395 (sh, 5.7), 422 (sh, 1.6), 500 (br, 3.1) | 707 (0.029) | 2.9 | 10.0 |
|  | 4% in mCP |  | 605 (0.2 (19%), 1.02 (81%)) | 90 | 10.5 |
|  | 2-MeTHF, 77 K |  | 603 (1.37 (42%), 3.93 (58%)) |  |  |
| **2** | Toluene | 312 (38.2), 371 (sh, 8.1), 393 (5.4), 461 (br, 4.5) | 678 (0.052) | 7 | 13.5 |
|  | 4% in mCP |  | 585 (0.33 (27%), 1.26 (73%)) | 86 | 8.5 |
|  | 2-MeTHF, 77 K |  | 579 (2.07 (38%), 5.39 (62%)) |  |  |
| **3** | Toluene | 317 (33.5), 382 (7.1), 401 (7.1), 439 (br, 4.5) | 634 (0.37) | 41.9 | 11.3 |
|  | 4% in mCP |  | 572 (0.13 (16%), 1.32 (84%)) | 88 | 7.8 |
|  | 2-MeTHF, 77 K |  | 580 (2.49 (38%), 8.35 (62%)) |  |  |
| **4** | Toluene | 298 (47.9), 311 (34.5), 322 (32.3), 372 (sh, 8.6), 393 (5.2), 475 (br, 4.0) | 714 (0.02) | 2.6 | 13.0 |
|  | 4% in mCP |  | 605 (0.22 (24%), 1.24 (76%)) | 81 | 8.2 |
|  | 2-MeTHF, 77 K |  | 602 (1.01 (18%), 3.04 (82%)) |  |  |
| **5** | Toluene | 296 (45.9), 311 (32.6), 320 (sh, 30.8), 372 (sh, 8.2), 393 (5.0), 474 (br, 3.8) | 713 (0.024) | 2.6 | 10.8 |
|  | 4% in mCP |  | 604 (0.23 (22%), 1.17 (78%)) | 81 | 8.4 |
|  | 2-MeTHF, 77 K |  | 609 (1.05 (22%), 2.98 (78%)) |  |  |


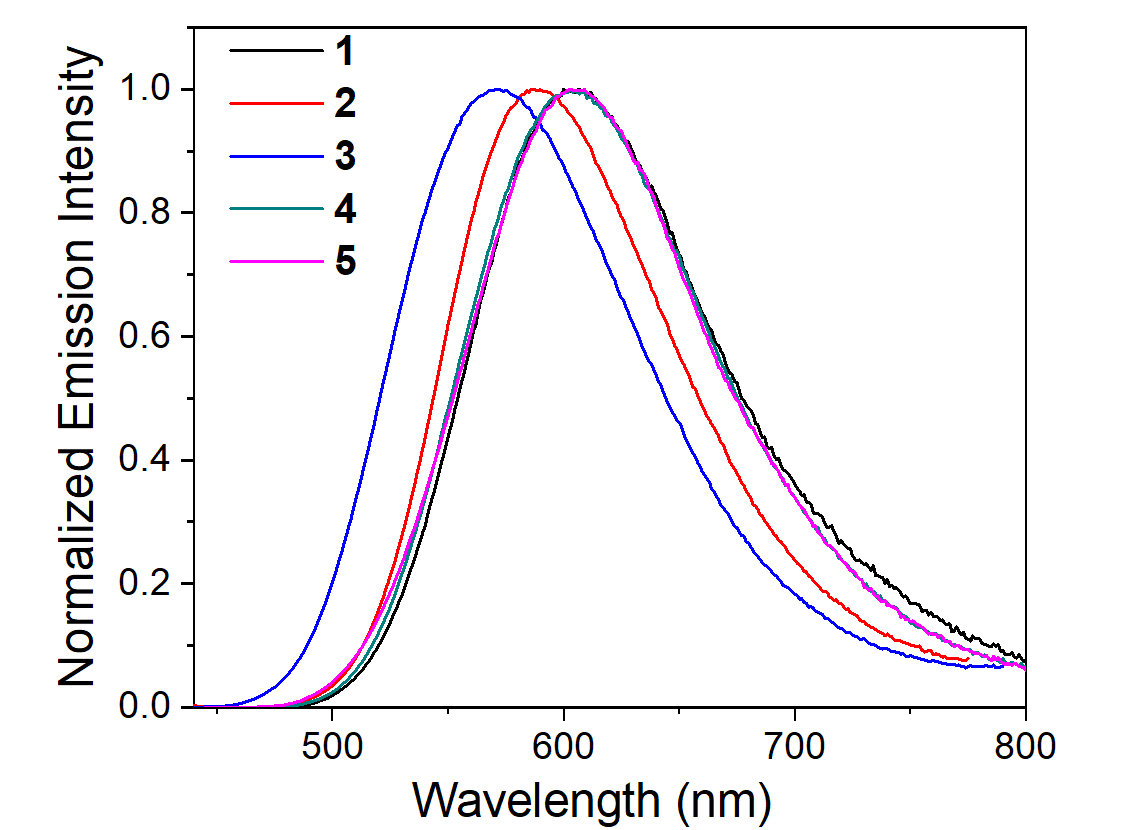


**Figure S4.** Emission spectra of **1**–**5** in mCP thin films (4 wt.%).


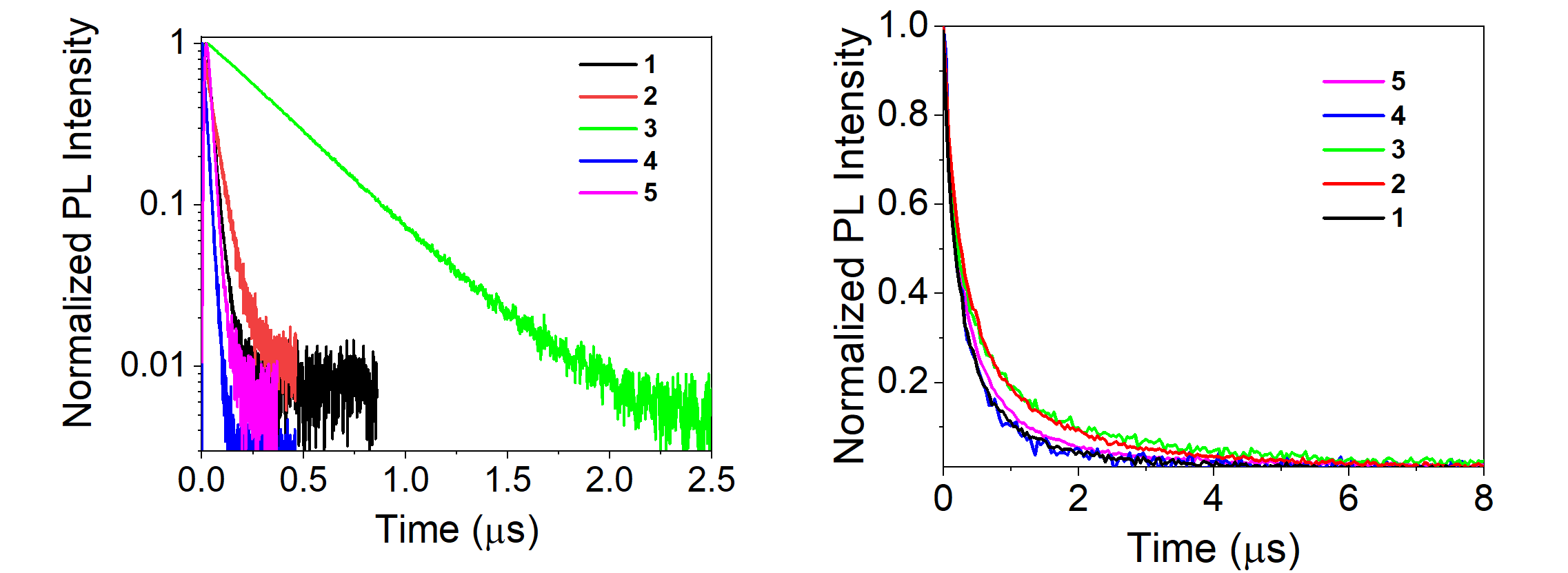


**Figure S5.**Transient emission intensity decays of **1**–**5** in toluene solution (left) and 4 wt.% mCP films (right) at 298 K.


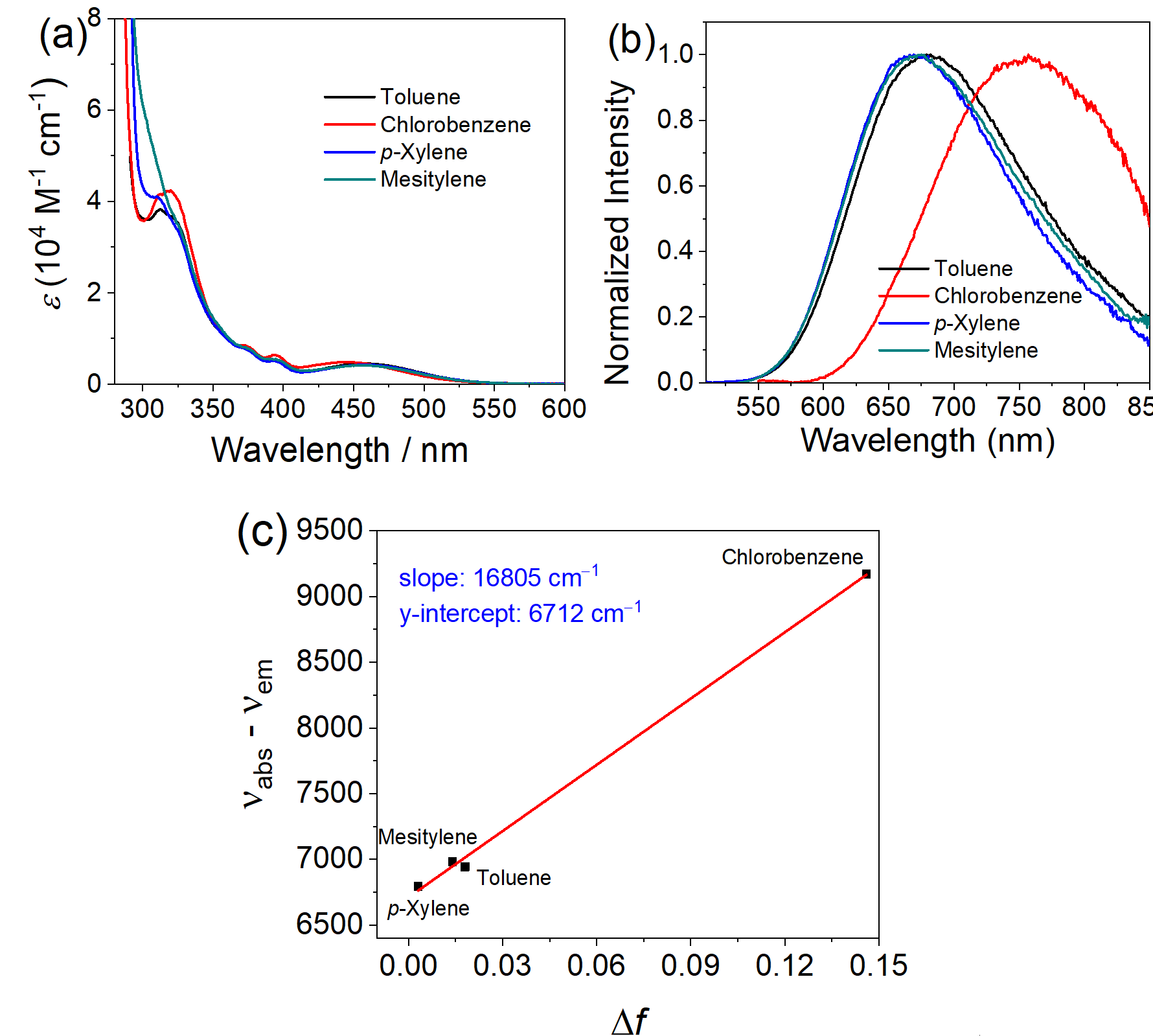


**Figure S6.** a) Absorption and b) emission spectra of complex **2** in different deoxygenated solvents (2 × 10^–5^ м) at room temperature, c) the Lippert-Mataga plot of **2**.


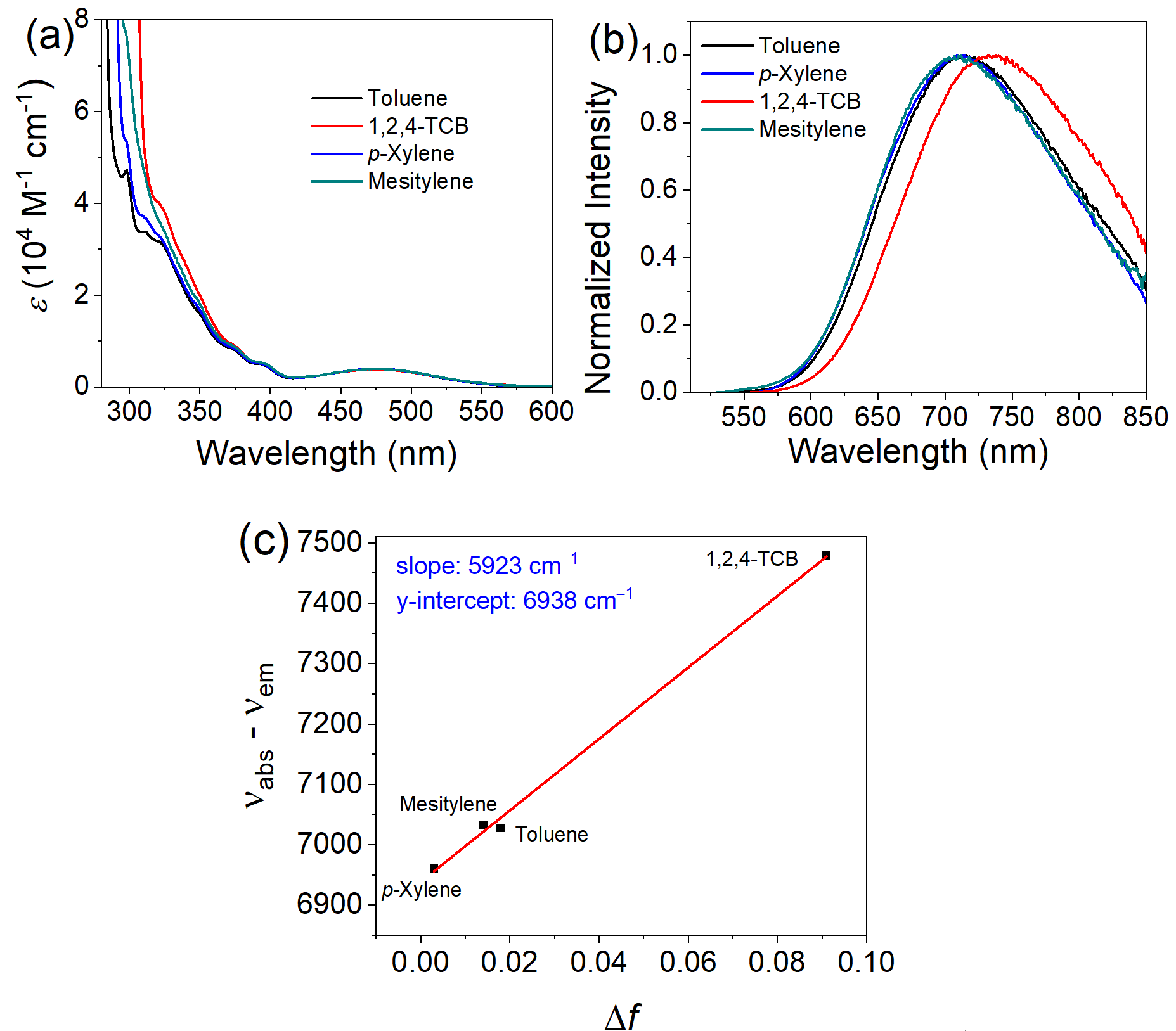


**Figure S7.** a) Absorption and b) emission spectra of complex **4** in different deoxygenated solvents (2 × 10^–5^ м) at room temperature, c) the Lippert-Mataga plot of **4**.

Variable-temperature emission lifetime measurement

The emission lifetimes of **1**–**4** in 2wt% PMMA thin films at temperature ranging from 77 K to 298 K were measured. The energy gap between singlet triplet states (Δ*E*_ST_) and the radiative decay rate constant of the S_1_ excited state ($k_{r}^{S_{1}}$) were estimated using the Arrhenius-type plots of In(*k*_TADF_) vs 1/T with lifetime data recorded at temperature above 200 K according to the following equation^2^.

In(*k*_TADF_) = ln($\frac{k_{\mathrm{ISC}}^{S_{1}}}{3}(1-\frac{k_{\mathrm{ISC}}^{S_{1}}}{k_{r}^{S_{1}}+k_{\mathrm{ISC}}^{S_{1}}})$)$-\frac{{\Delta E}_{\mathrm{ST}}}{k_{B}T}$, where $k_{r}^{S_{1}}= \frac{3bk_{\mathrm{ISC}}^{S_{1}}}{k_{\mathrm{ISC}}^{S_{1}}-3b}$

Since $k_{\mathrm{ISC}}^{S_{1}}\gg b, it can be estimated that k_{r}^{S_{1}}\approx3b$


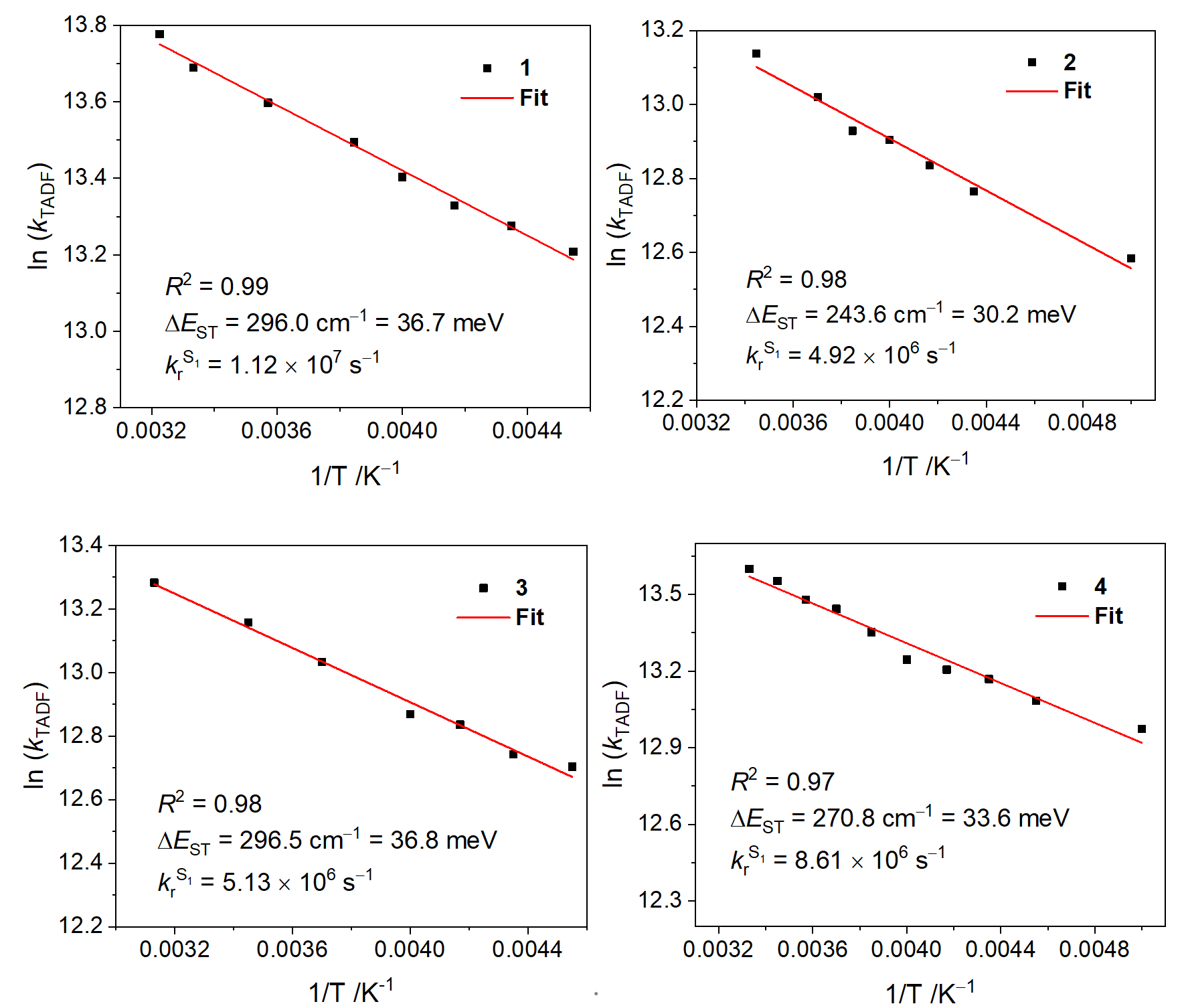


**Figure S8.** Plots of ln(*k*_TADF_) *versus* 1/T of **1**–**4** in 2 wt.% PMMA thin films

Time-resolved Transient Absorption Spectroscopy

**Nanosecond transient absorption (ns-TA)**


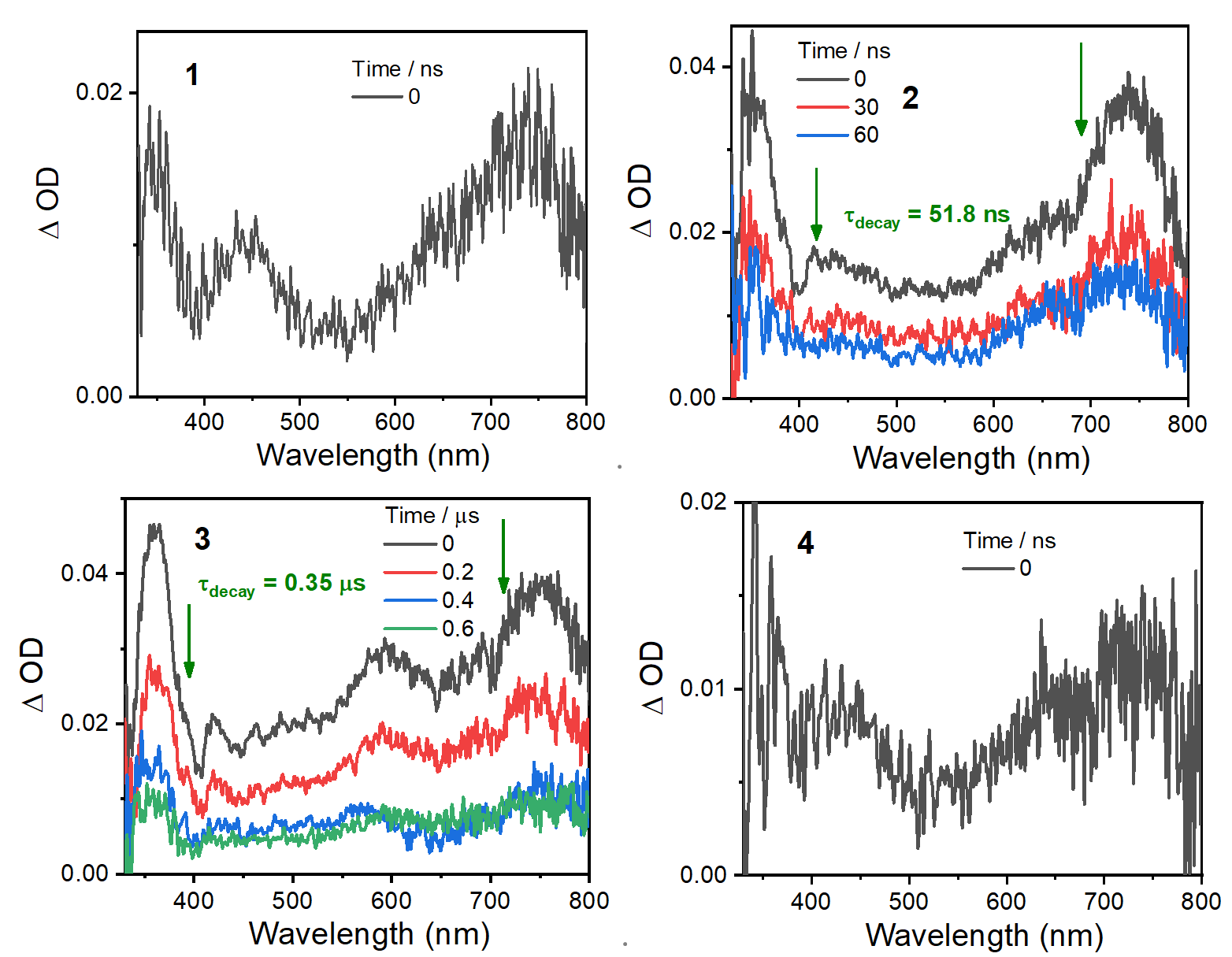


**Figure S9.** ns-TA spectra of **1**–**4** in deoxygenated toluene (*λ*_excitation_ = 355 nm) at room temperature.

**Femtosecond transient absorption (fs-TA)**


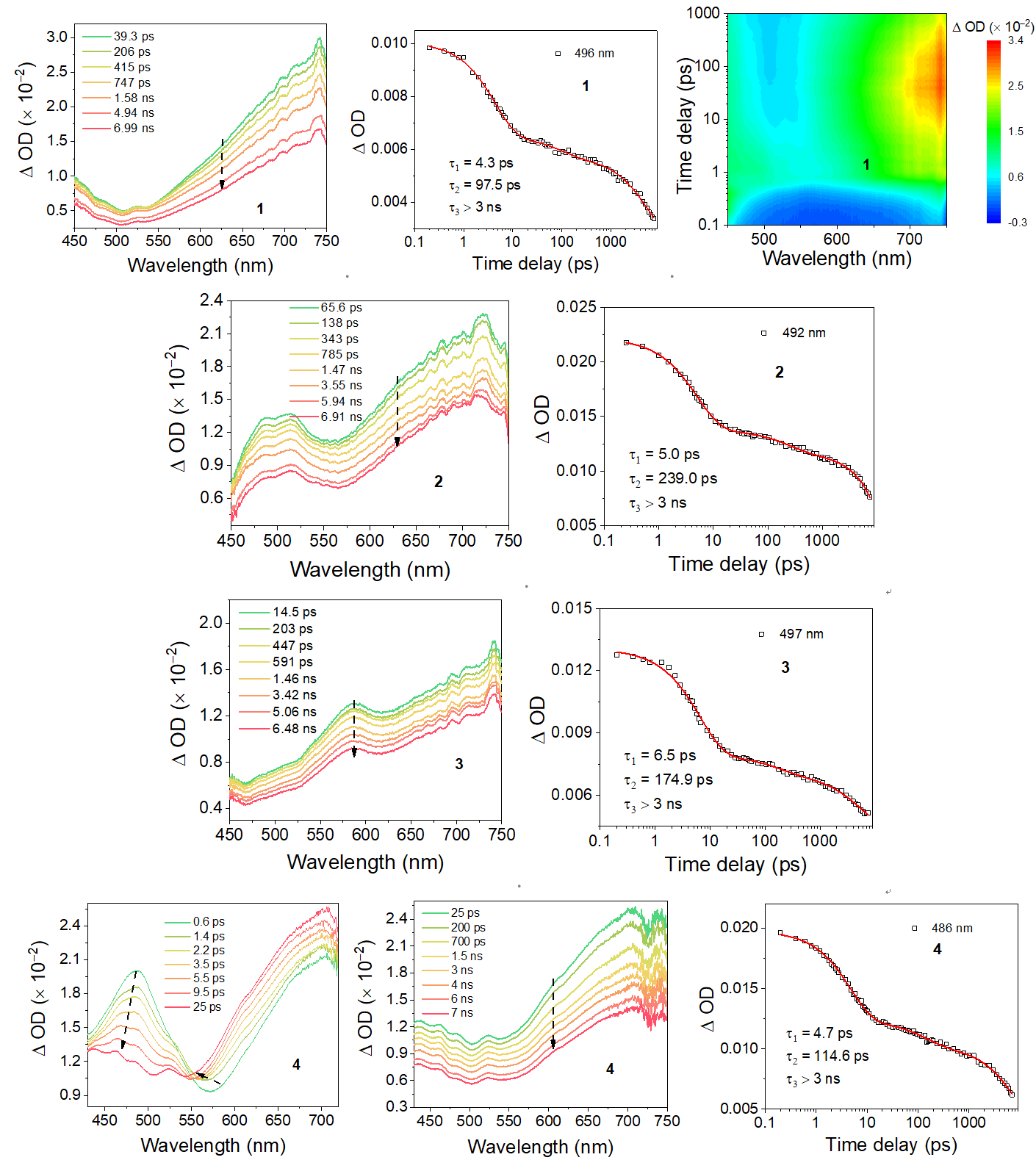


**Figure S10.** Late-stage ESA2 spectra of **1**–**3** and fs-TA spectra of **4** in toluene (*λ*_excitation_ = 370 nm) at room temperature.

**Femtosecond time-resolved fluorescence (fs-TRF)**


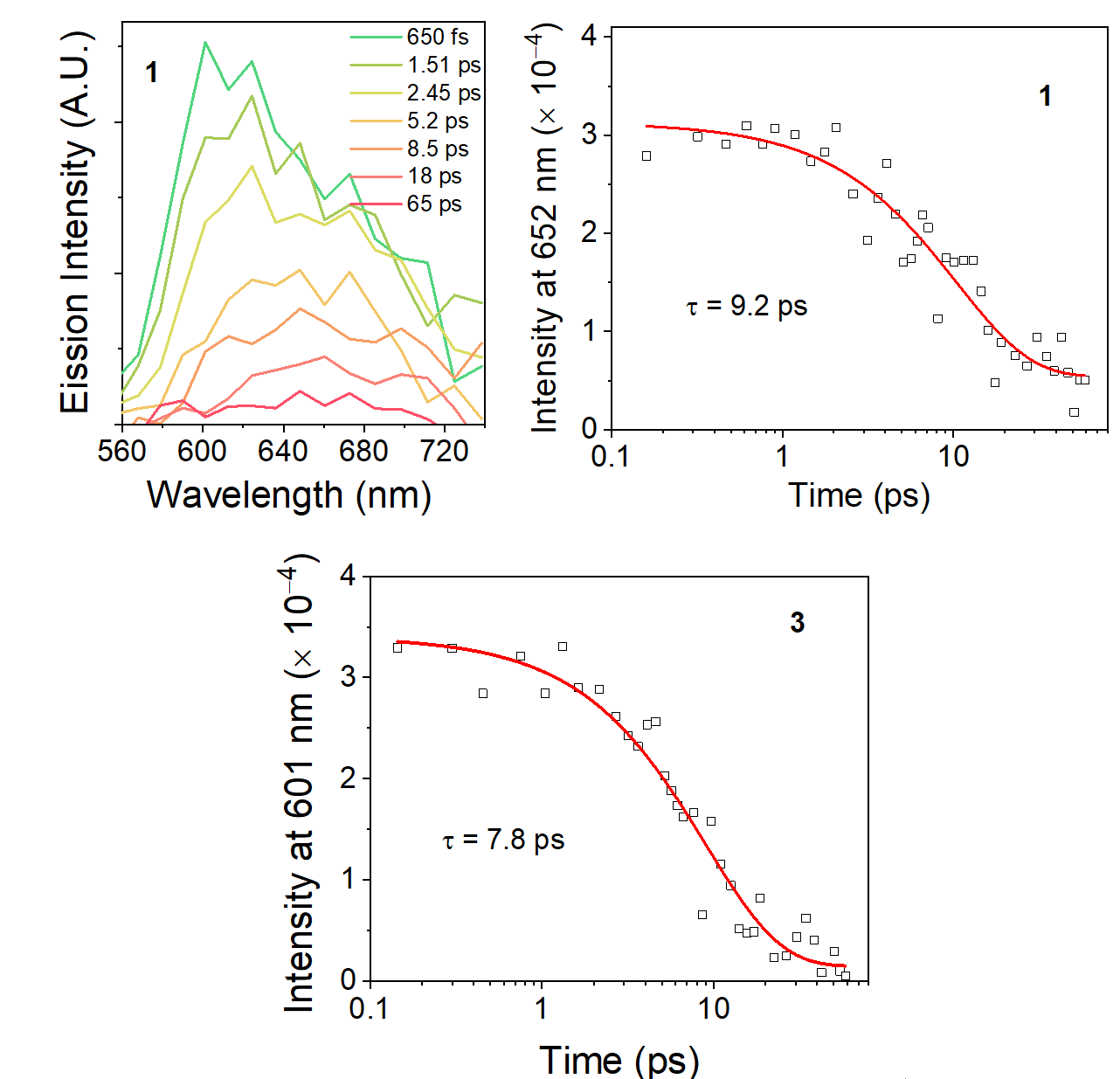


**Figure S11.** Femtosecond time-resolved fluorescence of **1** and **3** in toluene (*λ*_excitation_ = 400 nm) at room temperature.

Computational details

DFT/TDDFT calculations including geometry optimization, excited state energy calculations, and oscillator strength *f* calculations were performed using Gaussian 16 program package.^3^ The hybrid density functional M06^4^ was used for calculations. The 6-31G* basis set^5-6^ was applied for all atoms except gold, which was described by Sttutgart/Dresden SDD basis set^7^ and the associated pseudopotentials. Solvent effects were considered by means of the polarizable continuum model (PCM)^8^ with toluene as the solvent. TDDFT calculations were performed for optimization of both the first singlet and triplet excited states, S_1_ and T_1_.

The triplet radiative rate constant (*k*_T1_) was calculated using the Amsterdam Density Functional (ADF) 2019 package.^9-11^ The hybrid density functional M06 was used for calculations. Zeroth order regular approximation (ZORA) ^12-14^ was applied to describe the scalar relativistic effect. All electron Slater-type orbital (STO) basis set of TZP (triple-ζ polarization functional)^15-16^ was applied for all atoms. Solvent effects were taken into account by means of conductor like screening model (COSMO)^17-18^ with toluene as the solvent.

To plot the excited state adiabatic potential energy surfaces (PES), we performed restricted geometry optimization of emitters with fixed torsional angles 𝜃_C1-Au-C2-C3_. The scan step is 10º and the scan range is 0º to 180º. The adiabatic energy at each point was extracted to plot the PES for both S_1_ and T_1_ states. In the PES plot, the energy of the coplanar T_1_ structure (𝜃_C1-Au-C2-C3_ = 0º/180º) was set as 0 eV for comparison.

Radiative decay rate constants *k*_S1_ for S_1_→S_0_ transitions were calculated by Einstein spontaneous emission rate formula:^19^

$$k_{S1}=\frac{1}{\tau}=\frac{f_{S1}{E_{S1,min-S0,min}}^{2}}{1.5}$$

where τ is the fluorescence lifetime, *f*_S1_ is the S_1_→S_0_ transition oscillator strength, *E*_S1,min-S0,min_ is the adiabatic energy difference between the global minimum of S_1_ and S_0_ states.

The average TADF emission rate (*k*_TADF,avg_) was calculated by assuming fast thermal equilibrium between all thermally accessible rotamers on the S_1_ and T_1_ PES (𝜃_C1-Au-C2-C3_ = 0º, 10º, 20º, …, 180º):

$$k_{TADF,avg}=\frac{\sum_{\theta} \left[ k_{S1,\theta}\exp\left( -\frac{{\Delta E}_{S1,\theta-\min}}{k_{B}T} \right)+3k_{T1,\theta}\exp\left( -\frac{{\Delta E}_{T1,\theta-\min}}{k_{B}T} \right) \right]}{\sum_{\theta} \left[ \exp\left( -\frac{{\Delta E}_{S1,\theta-\min}}{k_{B}T} \right)+3\exp\left( -\frac{{\Delta E}_{T1,\theta-\min}}{k_{B}T} \right) \right]}$$

where *k*_S1,_*_θ_* and *k*_T1._*_θ_* are radiative decay rate constants for S_1_→S_0_ and T_1_→S_0_ transitions at specific torsional angles, Δ*E*_S1,_*_θ_*_-min_ and Δ*E*_T1,_*_θ_*_-min_ are the energy differences between the specific S_1_ and T_1_ rotamers and the overall global energy minimum of both excited states.

The hole and electron distribution, the overlap between hole and electron (*O*_h,e_), the distance between centroids of hole and electron (Δ*r*), and the transition density plots were calculated using the program Multiwfn 3.8(dev)^20-21^ with electronic wavefunctions generated by the Gaussian 16 program package. The isosurface maps were rendered by VMD 1.9.3 software.^22^

**Figure S12.** DFT/TDDFT optimized structures of **2** and **3** in the ground and excited states.

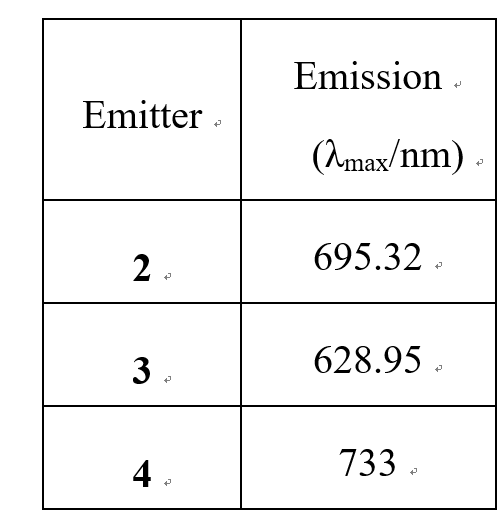


**Figure S13.** HOMO and LUMO of emitters **2** and **3** calculated using twisted (a) and orthogonal (b) geometries in the ground state. (c) HOMO and LUMO of **2** and **4** in the ground states calculated using semi-coplanar geometries (left) and emission wavelength (right) of **2**, **3** and **4** in the excited states calculated using semi-coplanar geometries.

**Table S6.** Calculated adiabatic energies of S_1_ and T_1_ states (*E*_S1,_*_θ_* and *E*_T1,_*_θ_*), adiabatic S_1_ and T_1_ energy gap (Δ*E*_ST,_*_θ_*), vertical energies of S_1_ and T_1_ states (*E*_S1(v),_*_θ_* and *E*_T1(v),_*_θ_*), oscillator strength for S_1_→S_0_ transition (*f*_S1,_*_θ_*), and radiative rate constants for S_1_→S_0_ and T_1_→S_0_ transitions (*k*_S1,_*_θ_* and *k*_T1,_*_θ_*) at different 𝜃_C1-Au-C2-C3_ angles, and TADF radiative rate constant (*k*_TADF,avg_) of **2**.

| *θ*  (º) | *E*_S1,_*_θ_*  (a.u.) | *E*_T1,_*_θ_*  (a.u.) | Δ*E*_ST,_*_θ_*  (eV) | *E*_S1(v),_*_θ_*  (eV) | *E*_T1(v),_*_θ_*  (eV) | *f*_S1,_*_θ_* | *k*_S1,_*_θ_*  (s^–1^) | *k*_T1,_*_θ_*  (s^–1^) |
| --- | --- | --- | --- | --- | --- | --- | --- | --- |
| 0 | –3912.72409 | –3912.72646 | 0.065 | 1.785 | 1.720 | 0.137 | 2.36 × 10^7^ | 3 |
| 10 | –3912.72390 | –3912.72640 | 0.068 | 1.783 | 1.721 | 0.134 | 2.32 × 10^7^ | 41 |
| 20 | –3912.72399 | –3912.72643 | 0.066 | 1.776 | 1.718 | 0.123 | 2.13 × 10^7^ | 197 |
| 30 | –3912.72433 | –3912.72633 | 0.054 | 1.765 | 1.716 | 0.104 | 1.80 × 10^7^ | 410 |
| 40 | –3912.72462 | –3912.72623 | 0.044 | 1.749 | 1.710 | 0.082 | 1.42 × 10^7^ | 590 |
| 50 | –3912.72500 | –3912.72602 | 0.028 | 1.732 | 1.703 | 0.059 | 1.01 × 10^7^ | 829 |
| 60 | –3912.72544 | –3912.72600 | 0.015 | 1.720 | 1.701 | 0.035 | 6.08 × 10^6^ | 916 |
| 70 | –3912.72556 | –3912.72596 | 0.011 | 1.707 | 1.698 | 0.016 | 2.74 × 10^6^ | 666 |
| 80 | –3912.72591 | –3912.72592 | 0.003 | 1.698 | 1.694 | 0.004 | 6.90 × 10^5^ | 169 |
| 90 | –3912.72591 | –3912.72614 | 0.0064 | 1.698 | 1.694 | 0.004 | 6.90 × 10^5^ | 10 |
| 100 | –3912.72594 | –3912.72612 | 0.0049 | 1.698 | 1.694 | 0.004 | 7.08 × 10^5^ | 298 |
| 110 | –3912.72558 | –3912.72608 | 0.013 | 1.705 | 1.696 | 0.018 | 3.09 × 10^6^ | 929 |
| 120 | –3912.72532 | –3912.72613 | 0.022 | 1.718 | 1.700 | 0.036 | 6.28 × 10^6^ | 923 |
| 130 | –3912.72485 | –3912.72619 | 0.037 | 1.732 | 1.705 | 0.059 | 1.02 × 10^7^ | 889 |
| 140 | –3912.72462 | –3912.72623 | 0.044 | 1.748 | 1.711 | 0.082 | 1.41 × 10^7^ | 641 |
| 150 | –3912.72429 | –3912.72631 | 0.055 | 1.761 | 1.713 | 0.105 | 1.81 × 10^7^ | 434 |
| 160 | –3912.72423 | –3912.72649 | 0.062 | 1.774 | 1.717 | 0.121 | 2.09 × 10^7^ | 214 |
| 170 | –3912.72406 | –3912.72652 | 0.067 | 1.780 | 1.719 | 0.132 | 2.28 × 10^7^ | 50 |
| 180 | –3912.72407 | –3912.72662 | 0.069 | 1.786 | 1.722 | 0.137 | 2.36 × 10^7^ | 6 |
| *k*_TADF,avg_ = 5.88 × 10^5^ s^–1^ | | | | | | | | |

Electroluminescence

Indium-tin-oxide (ITO) coated glass with a sheet resistance of 10 Ω/sq was used as the anode substrate. Before film deposition, patterned ITO substrates were cleaned with detergent, rinsed in de-ionized water, acetone, and isopropanol, and then dried in an oven for 1 h in a cleanroom. The slides were then treated in an ultraviolet-ozone chamber for 5 min. The OLEDs were fabricated in a Kurt J. Lesker SPECTROS vacuum deposition system with a base pressure of 10^–7^ mbar. In the vacuum chamber, organic materials were thermally deposited in sequence at a rate of 0.5 Å s^–1^. The doping process in the EMLs was realized using co-deposition technology. Afterward, Yb (1.0 nm)/Ag (100 nm) were thermally deposited at rates of 0.01 and 0.2 nm s^–1^, respectively. The film thickness was determined in situ with calibrated oscillating quartz-crystal sensors. Current density-brightness-voltage characteristics, EL spectra, and EQE of EL devices were obtained by using a Keithley 2400 source-meter and an absolute external quantum efficiency measurement system (C9920-12, Hamamatsu Photonics). All devices were encapsulated in a 200-nm-thick Al_2_O_3_ thin film deposited by atomic layer deposition (ALD) in a Kurt J. Lesker SPECTROS ALD system before measurements.

**Device structure used for OLEDs fabrication:**

**Device structure 1:**

ITO/FSFA: NDP-9 (3 wt.%) (10 nm)/FSFA (120 nm)/NBP-BC (5 nm)/Au^III^ emitter: NBP-BC: PCPF-Trz (40 nm)/ANT-Biz: Liq (1:1, 30 nm)/Yb (1 nm)/Ag (100 nm) ***Operational lifetime measurements were also conducted using this device structure.***

**Another device structure of OLEDs with 3 and 5 for estimating operational stability**

**Device structure 2:**

ITO/FSFA: NDP-9 (3 wt%) (10 nm)/FSFA (120 nm)/NPB-BC (5 nm)/Au^III^ emitter: RH /ANT-Biz: Liq (1:1, 30 nm)/Yb (1 nm)/Ag (100 nm)

**Chemical structures of organic materials used in this work:**


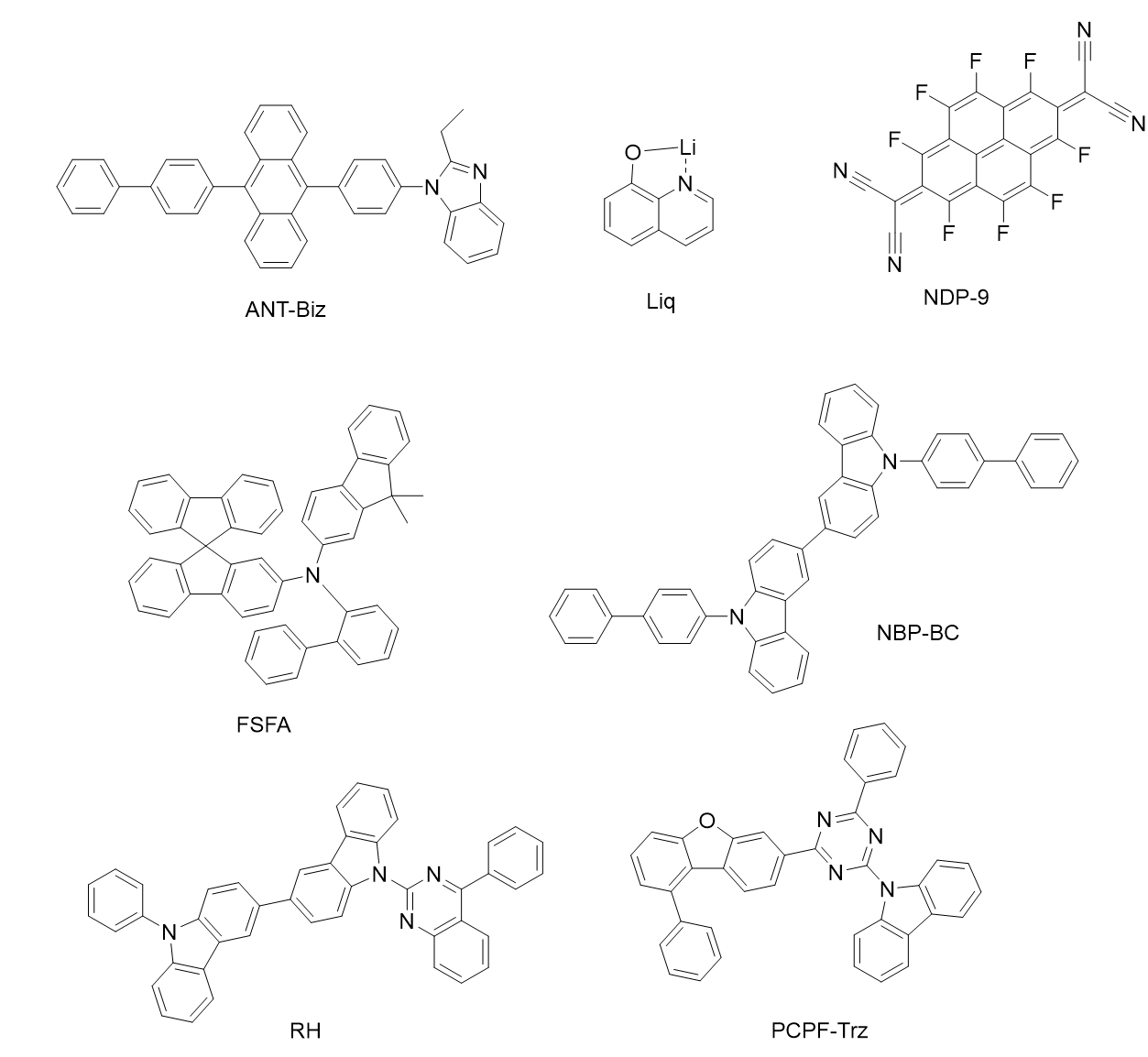


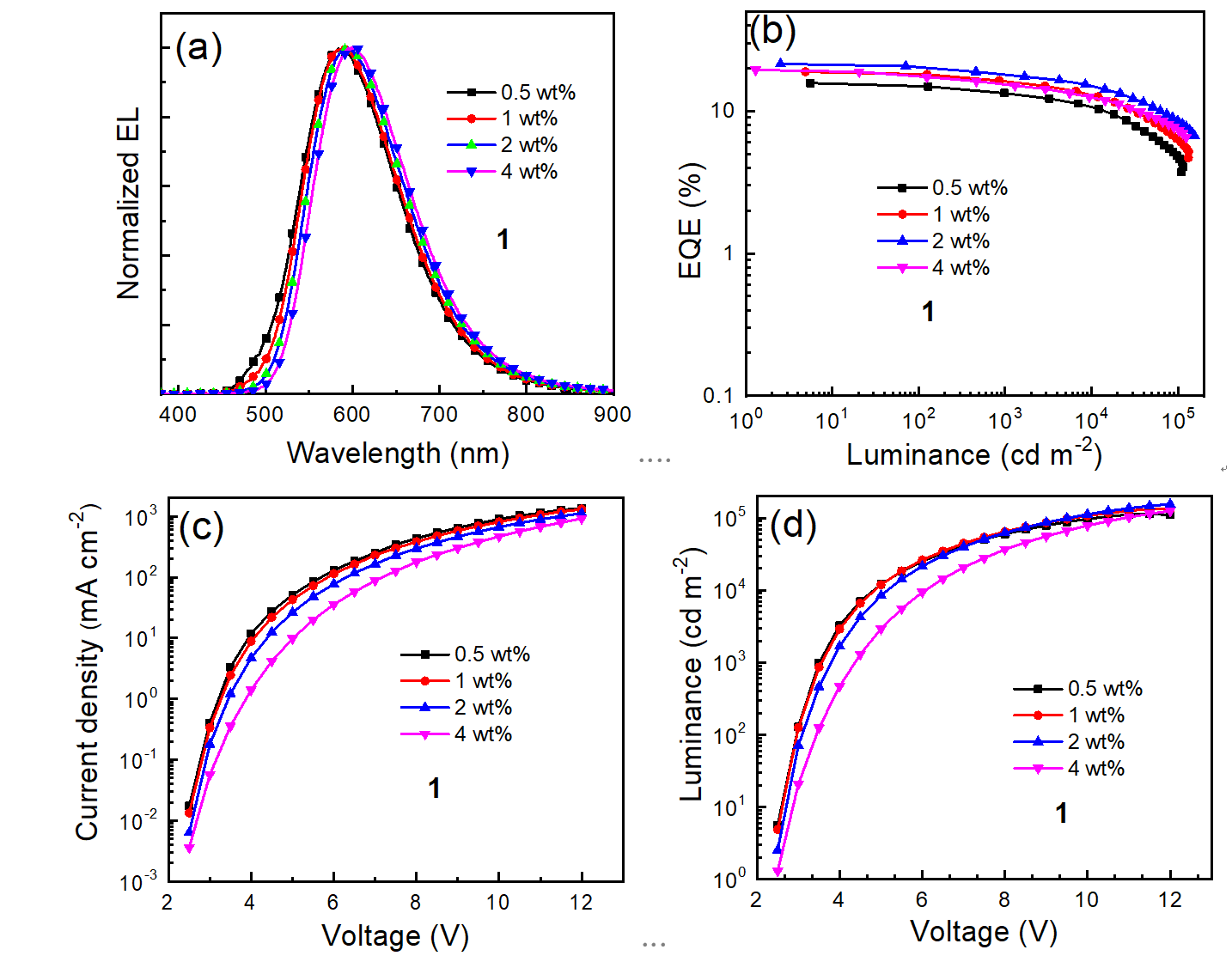


**Figure S14.** a) Normalized EL spectra, b) EQE-luminance, c) current density-voltage, and d) luminance-voltage characteristics of OLEDs based on **1** with various dopant concentrations (device structure 1).


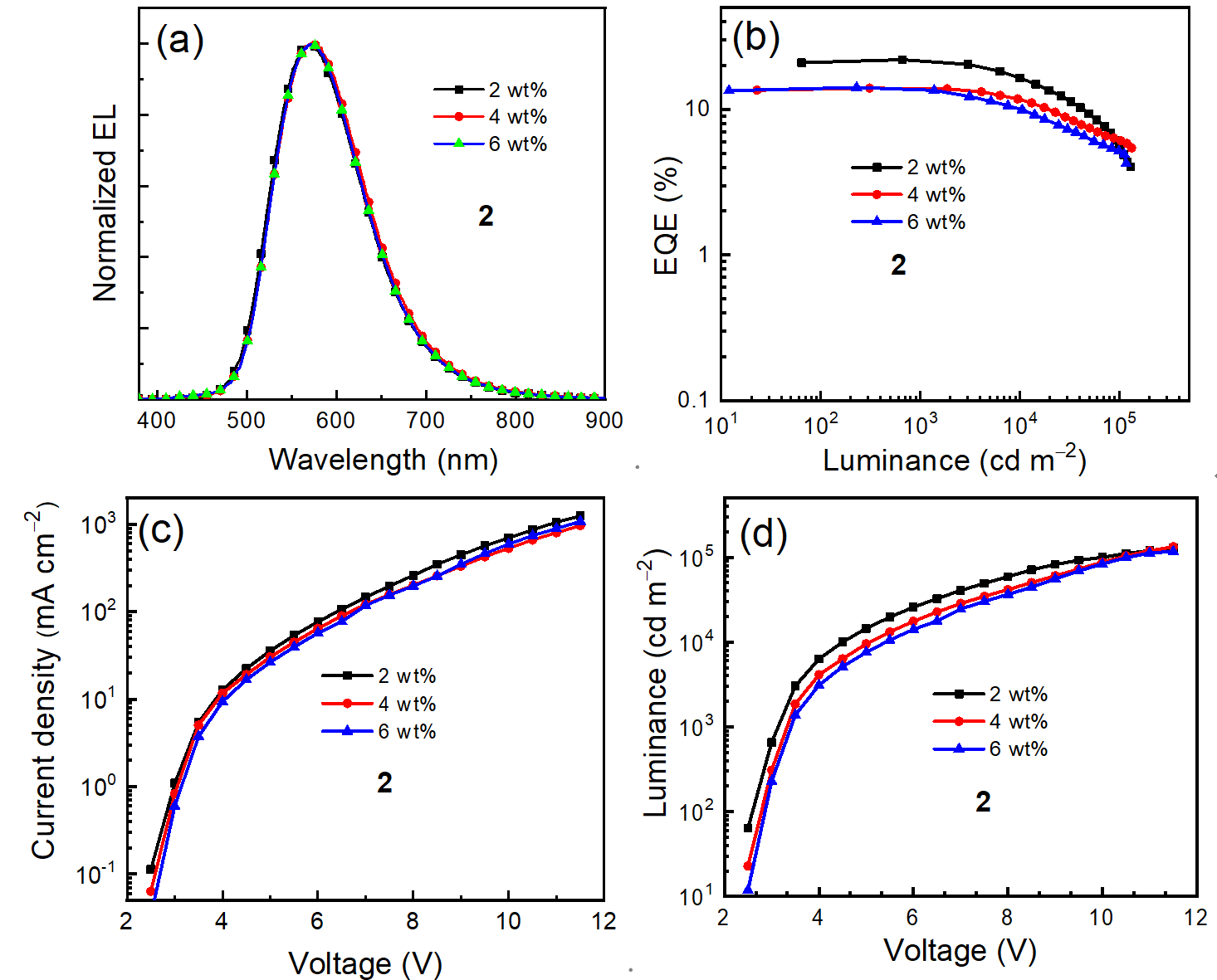


**Figure S15.** a) Normalized EL spectra, b) EQE-luminance, c) current density-voltage, and d) luminance-voltage characteristics of OLEDs based on **2** with various dopant concentrations (device structure 1).


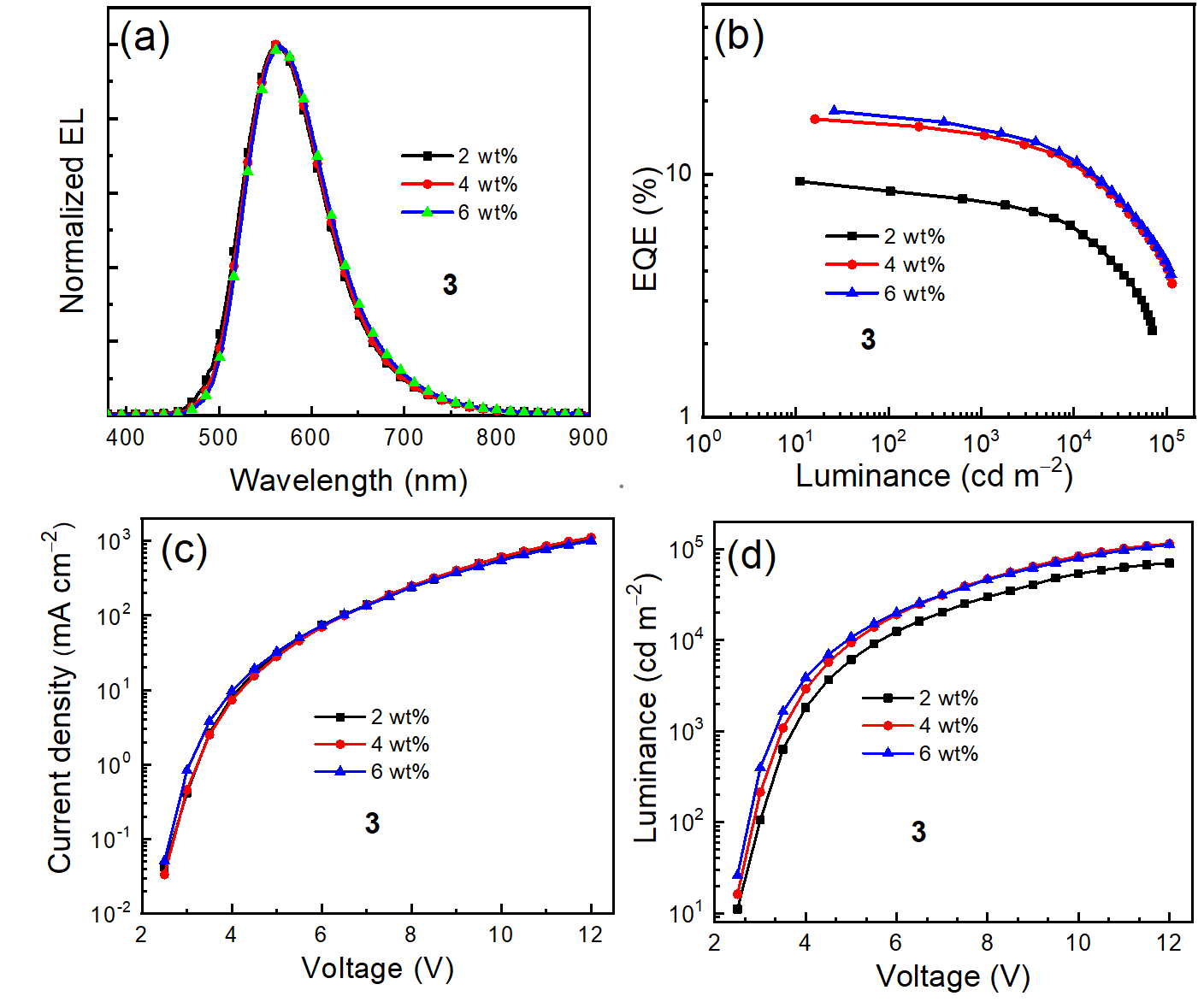


**Figure S16.** a) Normalized EL spectra, b) EQE-luminance, c) current density-voltage, and d) luminance-voltage characteristics of OLEDs based on **3** with various dopant concentrations (device structure 1).


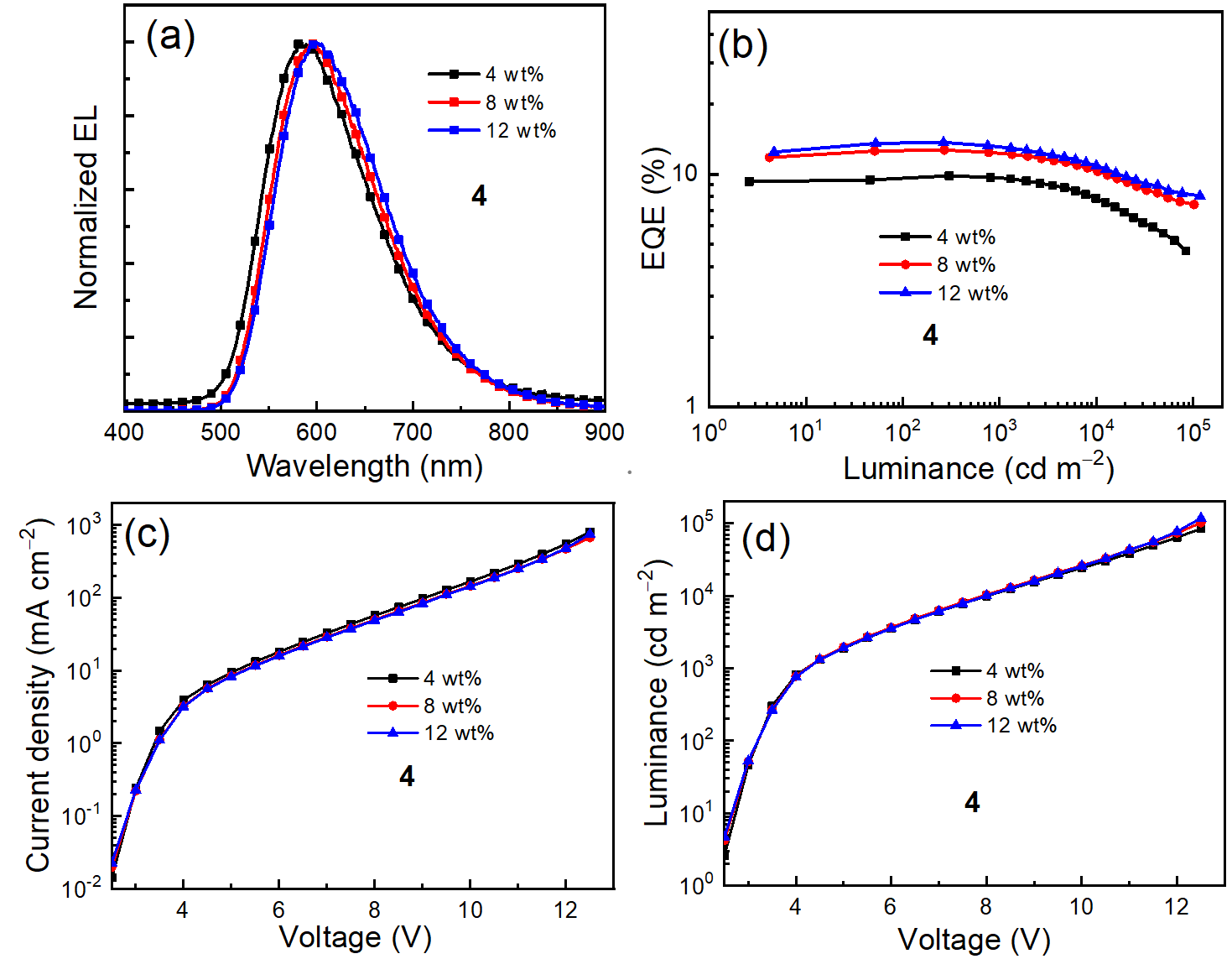


**Figure S17.** a) Normalized EL spectra, b) EQE-luminance, c) current density-voltage, and d) luminance-voltage characteristics of OLEDs based on **4** with various dopant concentrations (device structure 1).


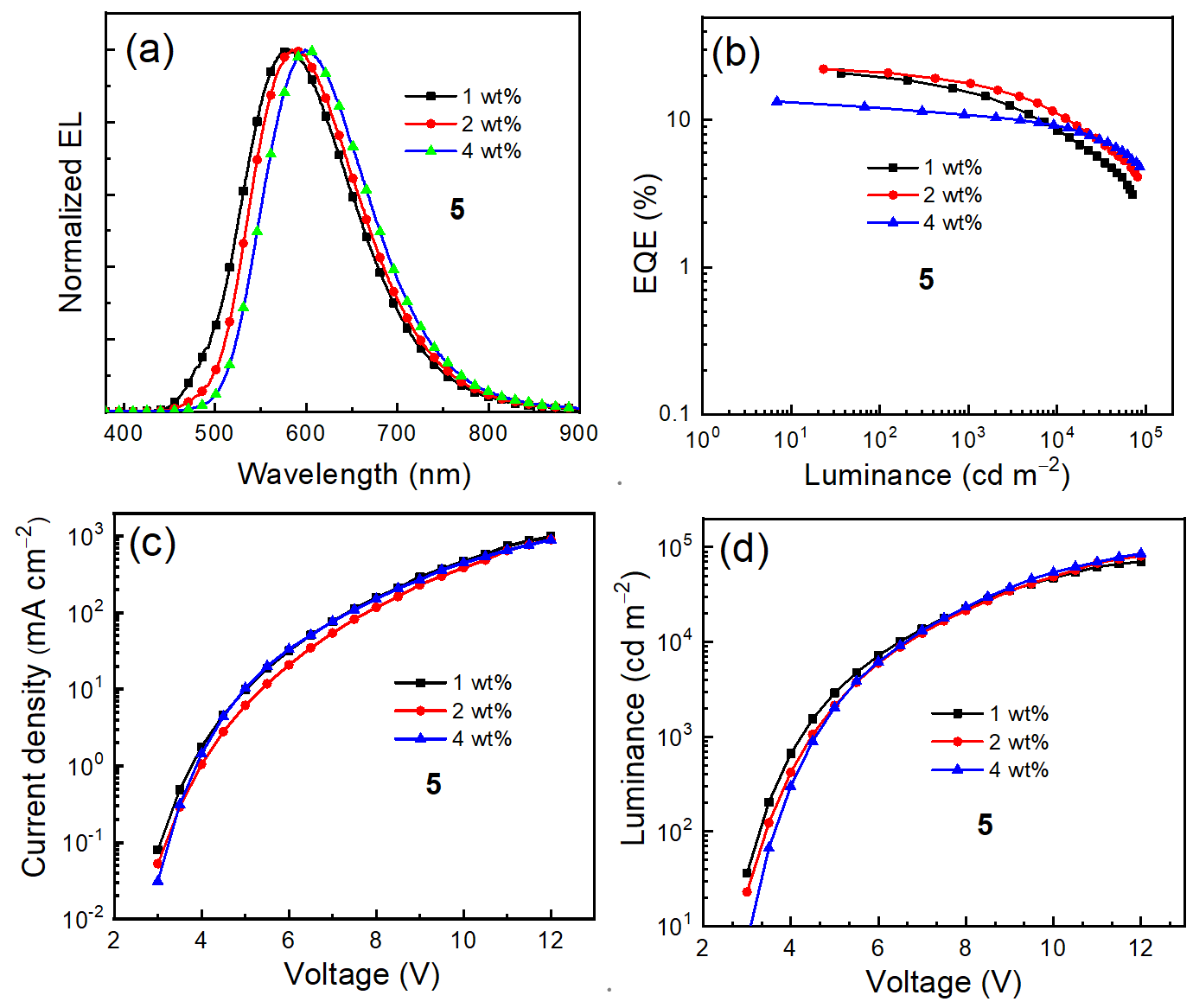


**Figure S18.** a) Normalized EL spectra, b) EQE-luminance, c) current density-voltage, and d) luminance-voltage characteristics of OLEDs based on **5** with various dopant concentrations (device structure 1).


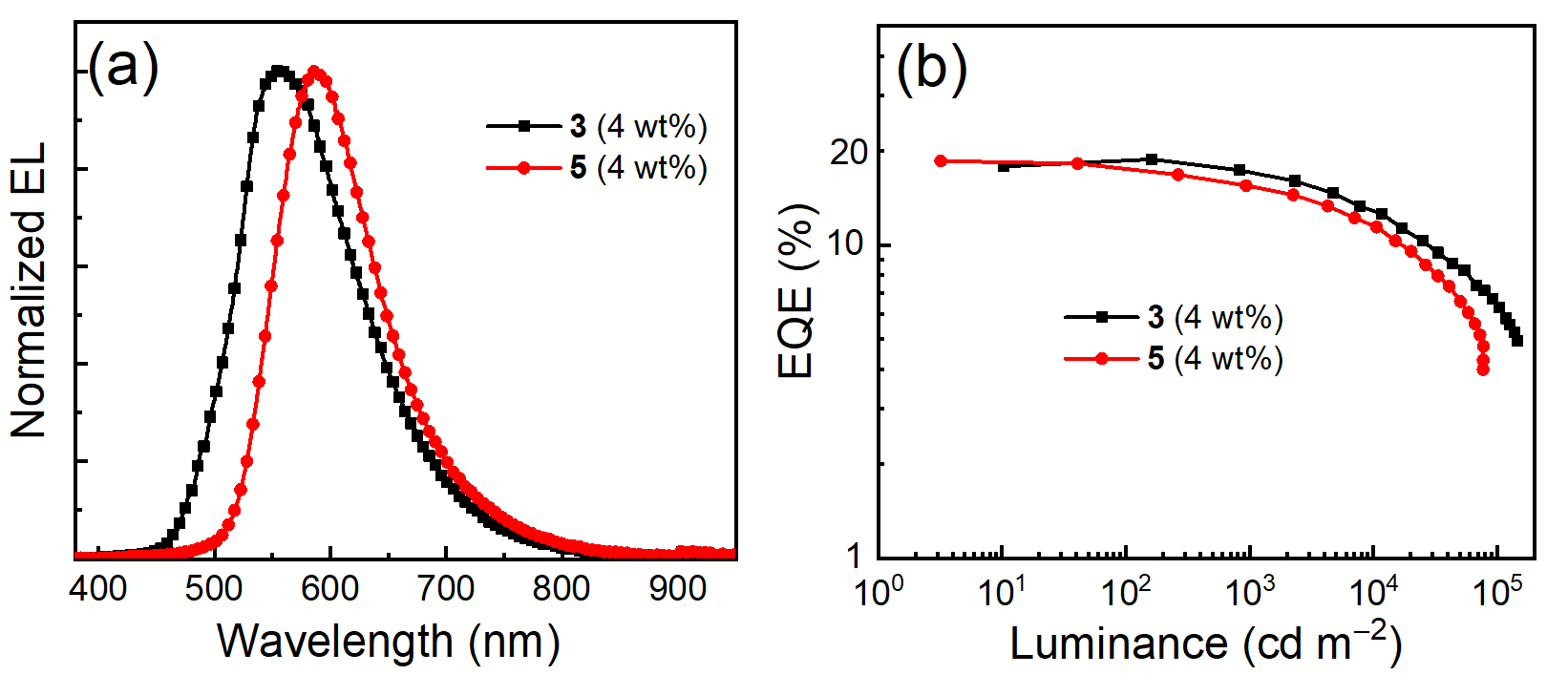


**Figure S19.** a) Normalized EL spectra OLEDs based on **3** (4 wt%), **5** (4 wt%) for lifetime measurement, and b) EQE-luminance based on device structure 2.


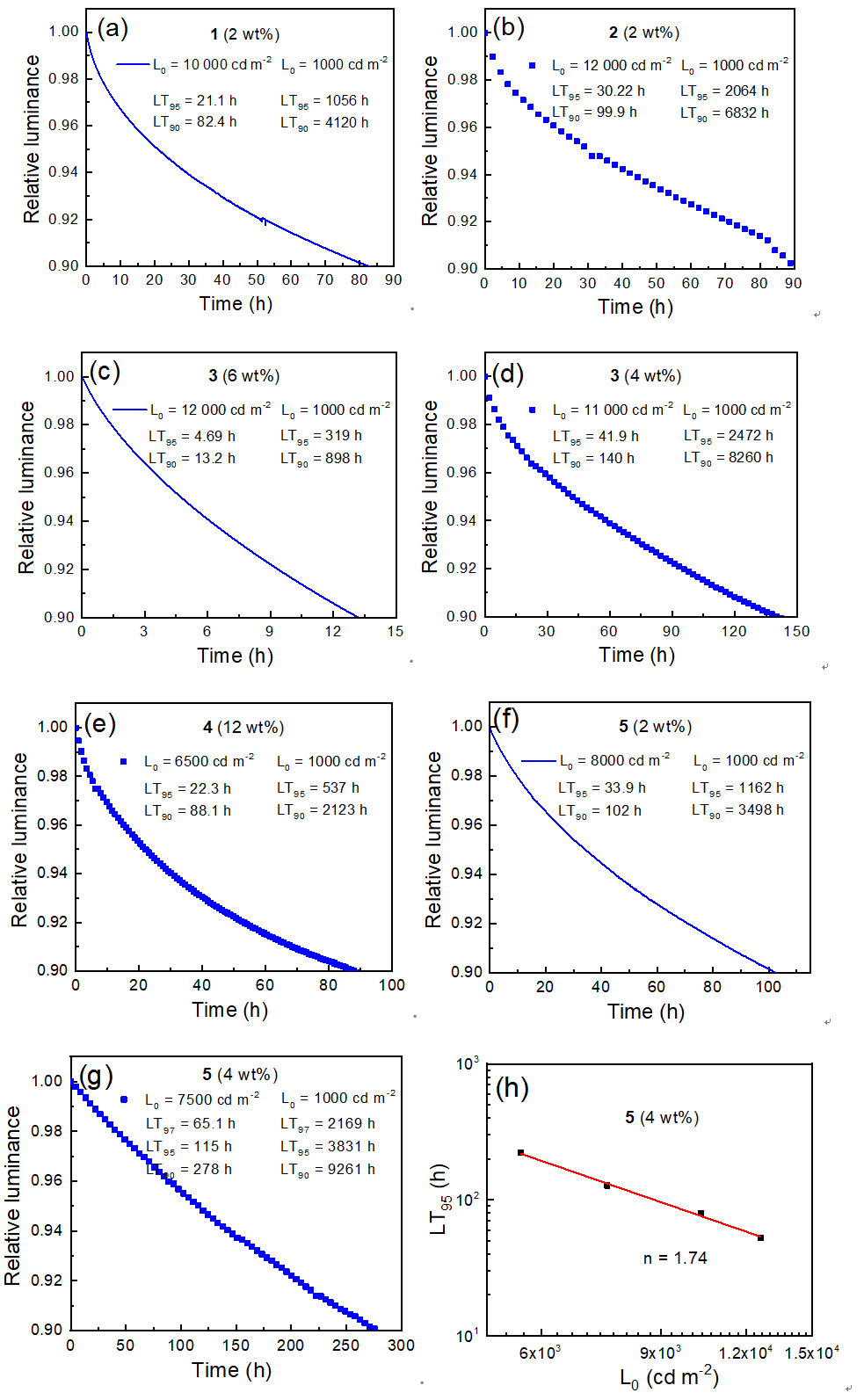


**Figure S20.** Relative luminance against operation time of devices based on a) **1**, b) **2**, c) **3**, e) **4**, f) **5** using device structure 1, and d) **3**, g) **5** using device structure 2. h) Acceleration coefficient fitting for OLED with complex **5** using device structure 2. The operational lifetimes (LT_95_/LT_90_) at 1000 cd m^–2^ for **1**–**4** are estimated from the equation LT(*L*_1_) = LT(*L*_0_)(*L*_0_/*L*_1_)*^n^*, where acceleration coefficient *n* is chosen to be 1.7.

**Table S7.** Operational lifetime of OLEDs with **1**–**5** as dopants.

| Emitter | *L*_0_^a)^ [cd m^–2^] | LT_95_^b)^ [h] | | LT_90_^c)^ [h] | |
| --- | --- | --- | --- | --- | --- |
|  |  | At *L*_0_ | At 1000 cd m^–2^ | At *L*_0_ | At 1000 cd m^–2^ |
| **1** (2 wt%)^d)^ | 10 000 | 21.1 | 1056 | 82.4 | 4120 |
| **2** (2 wt%)^d)^ | 12 000 | 30.2 | 2064 | 99.9 | 6832 |
| **3** (4 wt%)^e)^ | 11 000 | 41.9 | 2474 | 140 | 8260 |
| **3** (6 wt%)^d)^ | 12 000 | 4.69 | 319 | 13.2 | 898 |
| **4** (12 wt%)^d)^ | 6500 | 22.3 | 537 | 88.1 | 2123 |
| **5** (4 wt%)^e)^ | 7500 | 115 | 3831 | 278 | 9261 |
| **5** (2 wt%)^d)^ | 8000 | 33.9 | 1162 | 102 | 3498 |

a) Initial luminance; b) Operational lifetime at 95% of initial luminance; c) Operational lifetime at 90% of initial luminance; d) Based on device structure 1; e) Based on device structure 2.

**Table S8.** Representative examples of the gold OLEDs, TADF-OLEDs, Ir-sensitized hyper-OLEDs and platinum OLEDs with *λ*_EL_ in the range of 515–620 nm.

| Emitter | EQE_max_ | LT_90_ [h]  @1000 cd m^–2^ | LT_95_ [h]  @1000 cd m^–2^ | LT_97_ [h]  @1000 cd m^–2^ | LT_98_ [h]  @1000 cd m^–2^ | Ref. |
| --- | --- | --- | --- | --- | --- | --- |
| **5** | 22.2% | 9261 | 3831 | 2019 | 1399 | **This work** |
| **PhPXZ-tCzBN**  **(Ir^III^-sensitized)** | 25.7% | 8632 | 3002 | - | - | 23 |
| **BNTPA**  **(Ir^III^-sensitized)** | 43.3% | - | - | - | 322 | 24 |
| **CMA-Au^I^**  **(TADF)** | 21.3% | - | 2082 | - | - | 25 |
| **Tetra-Au^III^**  **(TADF)** | 23.7% | 1820 | - | - | - | 26 |
| **Pincer-Au^III^**  **(TADF)** | 18.0% | 606 | 200 | - | - | 27 |
| **Pincer-Pd^II^**  **(TADF)** | 27.5% | - | 436 | - | - | 28 |
| **CMA-Cu^I^**  **(TADF)** | 14.4% | 1300 | - | - | - | 29 |
| **Dinuclear Pt^II^**  **(Phos.)** | 19.3% | - | - | 2446 | - | 30 |
| **5TCzBN**  **(Ir^III^-sensitized)** | 21.3% | 6278^a)^ | - | - | - | 31 |
| **Tetra-Pt^II^**  **(Phos.)** | 16.9% | - | - | 2057 | - | 32 |
| **MR-NBNN2**  **(TADF)** | 31.7% | - | 85.2 | - | - | 33 |

^a)^ The estimated operational lifetime (LT_90_) at 1000 cd m^–2^ for reference 31 is calculated with an acceleration coefficient of 1.7.

References

[1] (a) S. Shi, M. C. Jung, C. Coburn, A. Tadle, D. Sylvinson M. R., P. I. Djurovich, S. R. Forrest, M. E. Thompson, *J. Am. Chem. Soc.* **2019**, *141*, 3576–3588.

[2] (a) R. Hamze, M. Idris, D. S. M. Ravinson, M. C. Jung, R. Haiges, P. I. Djurovich, M. E. Thompson, *Front. Chem.* **2020**, *8*, 401; (b) R. Hamze, S. Shi, S. C. Kapper, D. S. M. Ravinson, L. Estergreen, M.-C. Jung, A. C. Tadle, R. Haiges, P. I. Djurovich, J. L. Peltier, R. Jazzar, G. Bertrand, S. E. Bradforth, M. E. Thompson, *J. Am. Chem. Soc.* **2019**, *141*, 8616–8626; (c) D. Di, A. S. Romanov, L. Yang, J. M. Richter, J. P. H. Rivett, S. Jones, T. H. Thomas, M. A. Jalebi, R. H. Friend, M. Linnolahti, M. Bochmann, D. Credgington, *Science* **2017**, *356*, 159–163; (d) A.-P. M. Reponen, F. Chotard, A. Lempelto, V. Shekhovtsev, D. Credgington, M. Cochmann, M. Linnolahti, N. C. Greenham, A. S. Romanov, *Adv. Opt. Mater.* **2022**, *10*, 2200312; (e) Q. Gu, F. Chotard, J. Eng, A.-P. M. Reponen, I. J. Vitorica-Yrezabal, A. W. Woodward, T. J. Penfold, D. Credgington, M. Bochmann, A. S. Romanov, *Chem. Mater.* **2022**, *34*, 7526–7542; (f) N. V. Tzouras, E. A. Martynova, X. Ma, T. Scattolin, B. Hupp, H. Busen, M. Saab, Z. Zhang, L. Falivene, G. Pisanò, K. V. Hecke, L. Cavallo, C. S. J. Cazin, A. Steffen, S. P. Nolan, *Chem. Eur. J.* **2021**, *27*, 11904–11911.

[3] M. J. Frisch, G. W. Trucks, H. B. Schlegel, G. E. Scuseria, M. A. Robb, J. R. Cheeseman, G. Scalmani, V. Barone, G. A. Petersson, H. Nakatsuji, X. Li, M. Caricato, A. V. Marenich, J. Bloino, B. G. Janesko, R. Gomperts, B. Mennucci, H. P. Hratchian, J. V. Ortiz, A. F. Izmaylov, J. L. Sonnenberg, Williams, F. Ding, F. Lipparini, F. Egidi, J. Goings, B. Peng, A. Petrone, T. Henderson, D. Ranasinghe, V. G. Zakrzewski, J. Gao, N. Rega, G. Zheng, W. Liang, M. Hada, M. Ehara, K. Toyota, R. Fukuda, J. Hasegawa, M. Ishida, T. Nakajima, Y. Honda, O. Kitao, H. Nakai, T. Vreven, K. Throssell, J. A. Montgomery Jr., J. E. Peralta, F. Ogliaro, M. J. Bearpark, J. J. Heyd, E. N. Brothers, K. N. Kudin, V. N. Staroverov, T. A. Keith, R. Kobayashi, J. Normand, K. Raghavachari, A. P. Rendell, J. C. Burant, S. S. Iyengar, J. Tomasi, M. Cossi, J. M. Millam, M. Klene, C. Adamo, R. Cammi, J. W. Ochterski, R. L. Martin, K. Morokuma, O. Farkas, J. B. Foresman, D. J. Fox, Gaussian Inc. Wallingford CT, **2016**.

[4] Y. Zhao, D. G. Truhlar, *Theor. Chem. Acc.* **2008**, *120*, 215–241.

[5] L. Radom, P. C. Hariharan, J. A. Pople, P. V. R. Schleyer, *J. Am. Chem. Soc.* **1973**, *95*, 6531–6544.

[6] M. J. Frisch, J. A. Pople, J. S. Binkley, *J. Chem. Phys.* **1984**, *80*, 3265–3269.

[7] D. Andrae, U. Häußermann, M. Dolg, H. Stoll, H. Preuß, *Theor. Chem. Acc.* **1990**, *77*, 123–141.

[8] M. Cossi, G. Scalmani, N. Rega, V. Barone, *J. Chem. Phys.* **2002**, *117*, 43–54.

[9] C. Fonseca Guerra, J. G. Snijders, G. te Velde, E. J. Baerends, *Theor. Chem. Acc.* **1998**, *99*, 391–403.

[10] G. te Velde, F. M. Bickelhaupt, E. J. Baerends, C. Fonseca Guerra, S. J. A. van Gisbergen, J. G. Snijders, T. Ziegler, *J. Comput. Chem.* **2001**, *22*, 931–967.

[11] E.J. Baerends, A. J. A. T. Ziegler, J. Autschbach, O. Baseggio, D. Bashford, A. Bérces, F.M. Bickelhaupt, C. Bo, P.M. Boerrigter, L. Cavallo, C. Daul, D.P. Chong, D.V. Chulhai, L. Deng, R.M. Dickson, J.M. Dieterich, D.E. Ellis, M. van Faassen, L. Fan, T.H. Fischer, C. Fonseca Guerra, M. Franchini, A. Ghysels, A. Giammona, S.J.A. van Gisbergen, A. Goez, A.W. Götz, J.A. Groeneveld, O.V. Gritsenko, M. Grüning, S. Gusarov, F.E. Harris, P. van den Hoek, Z. Hu, C.R. Jacob, H. Jacobsen, L. Jensen, L. Joubert, J.W. Kaminski, G. van Kessel, C. König, F. Kootstra, A. Kovalenko, M.V. Krykunov, E. van Lenthe, D.A. McCormack, A. Michalak, M. Mitoraj, S.M. Morton, J. Neugebauer, V.P. Nicu, L. Noodleman, V.P. Osinga, S. Patchkovskii, M. Pavanello, C.A. Peeples, P.H.T. Philipsen, D. Post, C.C. Pye, H. Ramanantoanina, P. Ramos, W. Ravenek, J.I. Rodríguez, P. Ros, R. Rüger, P.R.T. Schipper, D. Schlüns, H. van Schoot, G. Schreckenbach, J.S. Seldenthuis, M. Seth, J.G. Snijders, M. Solà, M. Stener, M. Swart, D. Swerhone, V. Tognetti, G. te Velde, P. Vernooijs, L. Versluis, L. Visscher, O. Visser, F. Wang, T.A. Wesolowski, E.M. van Wezenbeek, G. Wiesenekker, S.K. Wolff, T.K. Woo, A. L. Yakovlev, *ADF 2019*, SCM, Theoretical Chemistry, Vrije Universiteit, Amsterdam, The Netherlands, **2019**.

[12] E. van Lenthe, E. J. Baerends, J. G. Snijders, *J. Chem. Phys.* **1993**, *99*, 4597–4610.

[13] E. van Lenthe, E. J. Baerends, J. G. Snijders, *J. Chem. Phys.* **1994**, *101*, 9783–9792.

[14] E. van Lenthe, A. Ehlers, E.-J. Baerends, *J. Chem. Phys.* **1999**, *110*, 8943–8953.

[15] P. L. Barbieri, P. A. Fantin, F. E. Jorge, *Mol. Phys.* **2006**, *104*, 2945–2954.

[16] S. F. Machado, G. G. Camiletti, A. C. Neto, F. E. Jorge, R. S. Jorge, *Mol. Phys.* **2009**, *107*, 1713–1727.

[17] A. Klamt, G. Schüürmann, *J. Chem. Soc., Perkin Trans. 2* **1993**, 799–805.

[18] A. Klamt, *J. Phys. Chem.* **1995**, *99*, 2224–2235.

[19] R. C. Hilborn, *Am. J. Phys.* **1982**, *50*, 982–986.

[20] T. Lu, F. Chen, *J. Comput. Chem.* **2012**, *33*, 580–592.

[21] Z. Liu, T. Lu, Q. Chen, *Carbon* **2020**, *165*, 461–467.

[22] W. Humphrey, A. Dalke, K. Schulten, *J. Mol. Graph.* **1996**, *14*, 33–38.

[23] H. Nemma, Y. Kori, N. Meguro, R. Mimura, Y. Chiba, J. Kido, H. Sasabe, *Adv. Optical Mater.* **2024**, 2402131

[24] L. Ge, W. Zhang, Y.-H. Hao, M. Li, Y. Liu, M. Zhou, L.-S. Cui, *J. Am. Chem. Soc.* **2024**, *146*, 32826.

[25] R. Tang, S. Xu, L. Du, F.-F. Hung, T.-L. Lam, G. Cheng, K.-H. Low, Q. Wan, S. Wu, Y. Chen, C.-M. Che, *Adv. Optical Mater.* **2023**, 2300950.

[26] D. Zhou, G. S. M. Tong, G. Cheng, Y.-K. Tang, W. Liu, D. Ma, L. Du, J.-R. Chen, C.-M. Che, *Adv. Mater.* **2022**, *34*, 2206598.

[27] C.-Y. Wong, S.-L. Lai, M.-Y. Leung, M.-C. Tang, L.-K. Li, M.-Y. Chan, V. W.-W. Yam, *J. Am. Chem. Soc.* **2023**, *145*, 2638.

[28] J.-G. Yang, X. Feng, N. Li, J. Li, X.-F. Song, M.-D. Li, G. Cui, J. Zhang, C. Jiang, C. Yang, K. Li, *Sci. Adv.* **2023**, *9*, eadh0198.

[29] R. Tang, S. Xu, T.-L. Lam, G. Cheng, L. Du, Q. Wan, J. Yang, F.-F. Hung, K.-H. Low, D. L. Phillips, C.-M. Che, *Angew. Chem. Int. Ed.* **2022**, *61*, e202203982; *Angew. Chem.* 2022, *134*, e202203982.

[30] M. Xue, T.-L. Lam, G. Cheng, W. Liu, K.-H. Low, L. Du, S. Xu, F.-F. Hung, D. L. Phillips, C.-M. Che, *Adv. Optical Mater.* **2022**, *10*, 2200741.

[31] D. Zhang, P. Wei, D. Zhang, L. Duan, *ACS Appl. Mater. Interfaces* **2017**, *9*, 19040.

[32] Z. Zhu, K. Klimes, S. Holloway, J. Li, *Adv. Mater.* **2017**, *29*, 1605002.

[33] S. Luo, J. Wang, N. Li, X.-F. Song, X. Wan, K. Li, C. Yang, *Angew. Chem. Int. Ed.* **2023**, *62*, e202310943; *Angew. Chem.* **2023**, *135*, e202310943.

NMR spectra


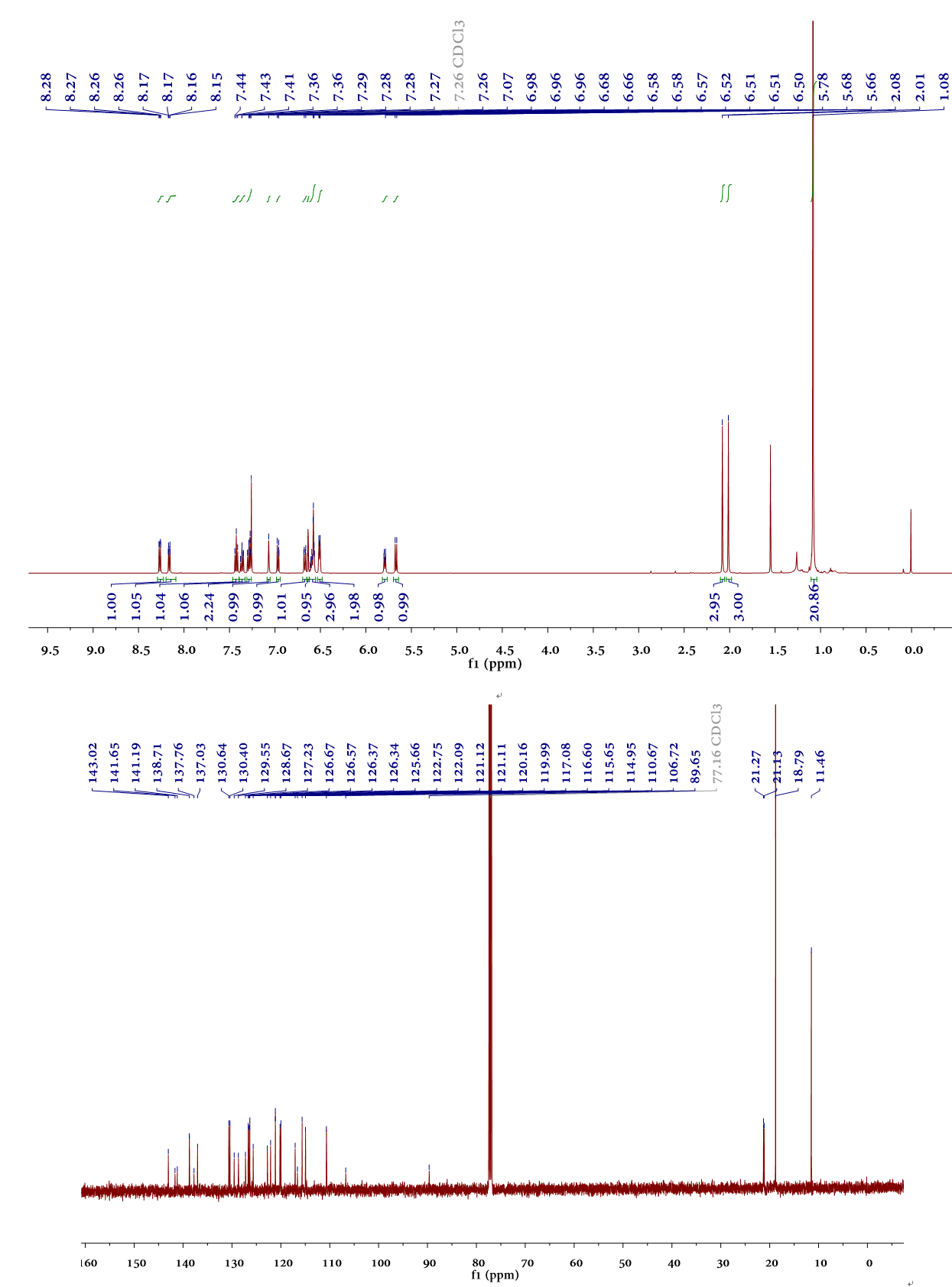


^1^H and ^13^C NMR spectra of L**1**


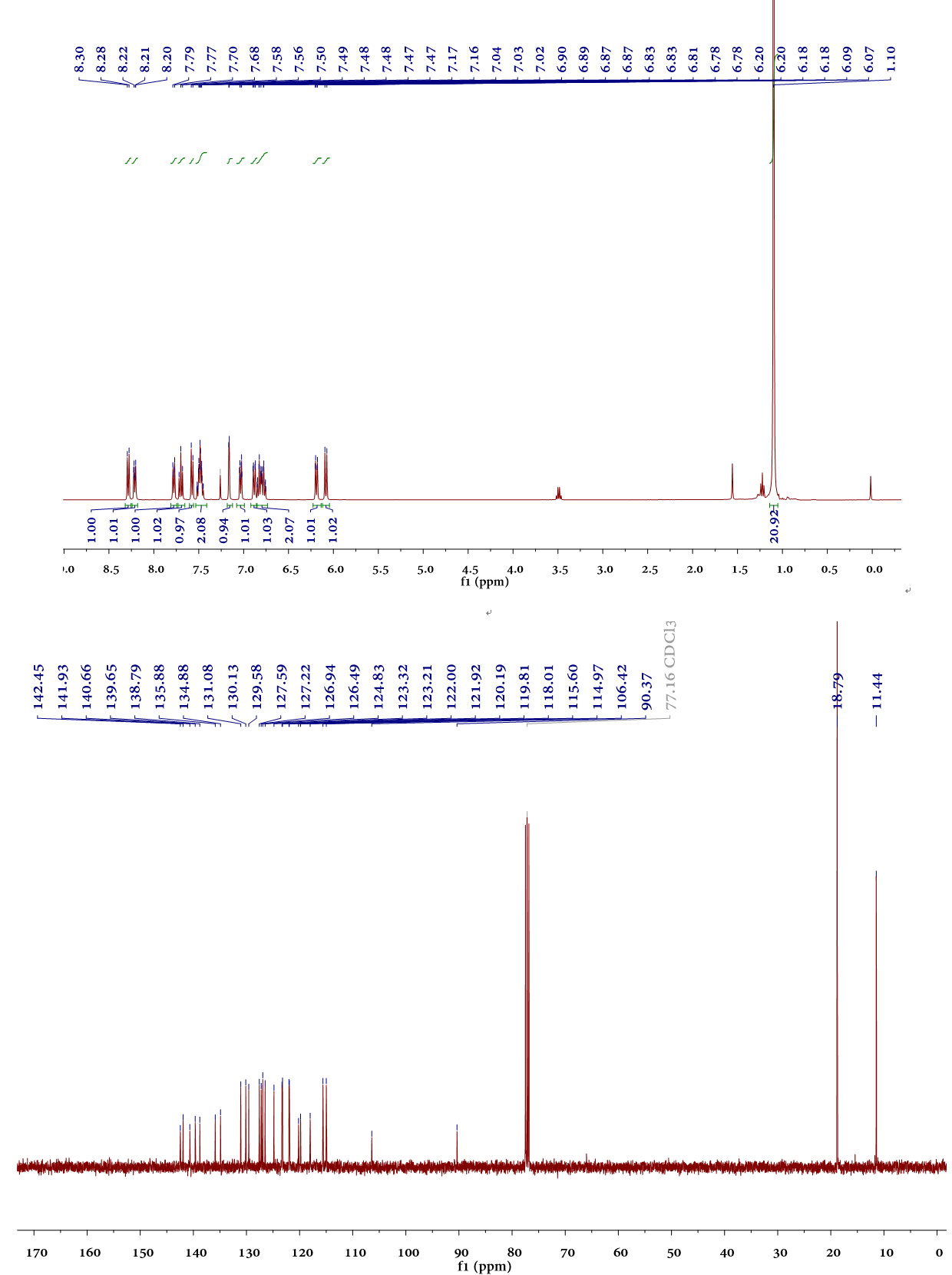


^1^H and ^13^C NMR spectra of L**2**


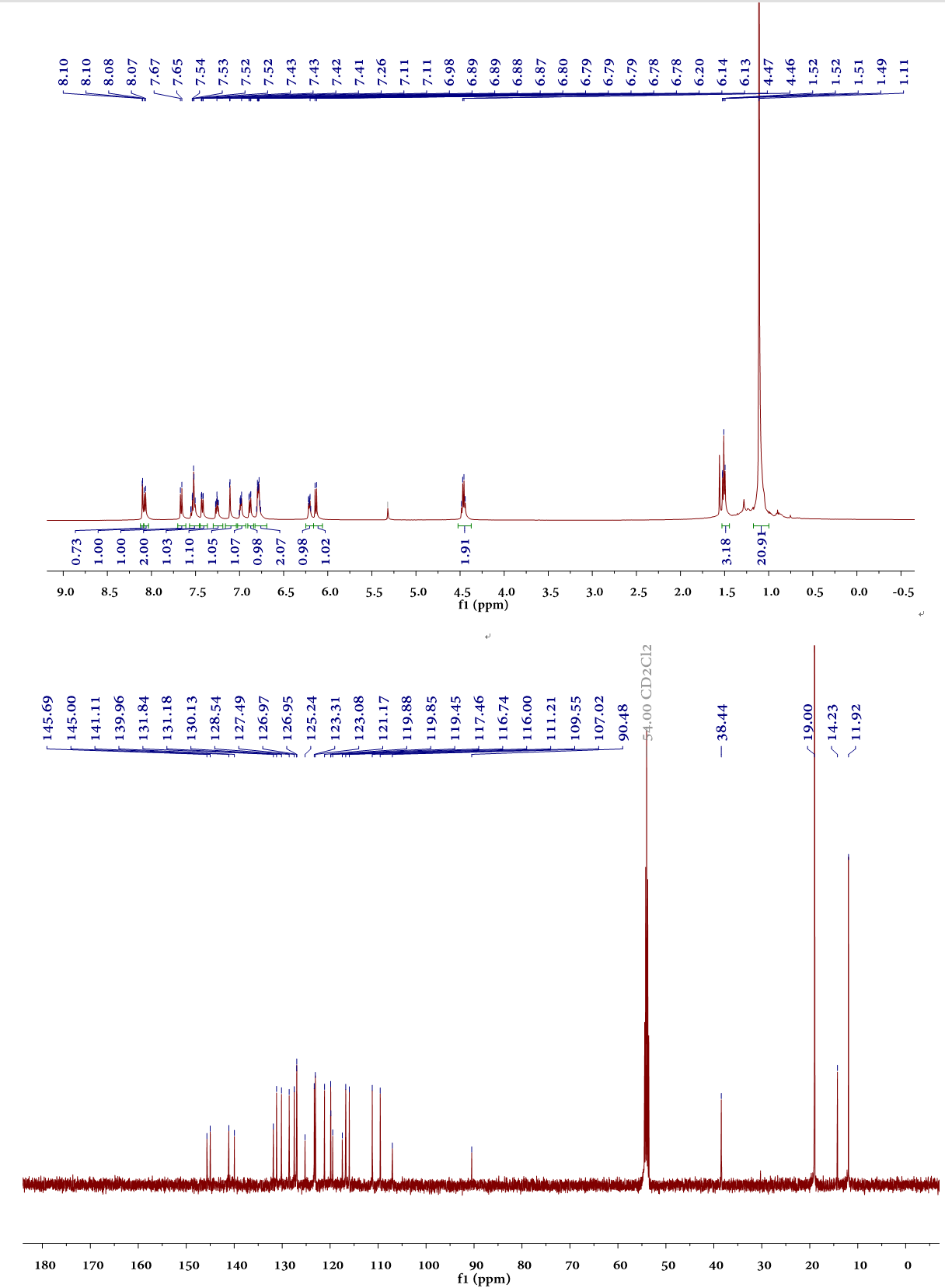


^1^H and ^13^C NMR spectra of L**3**


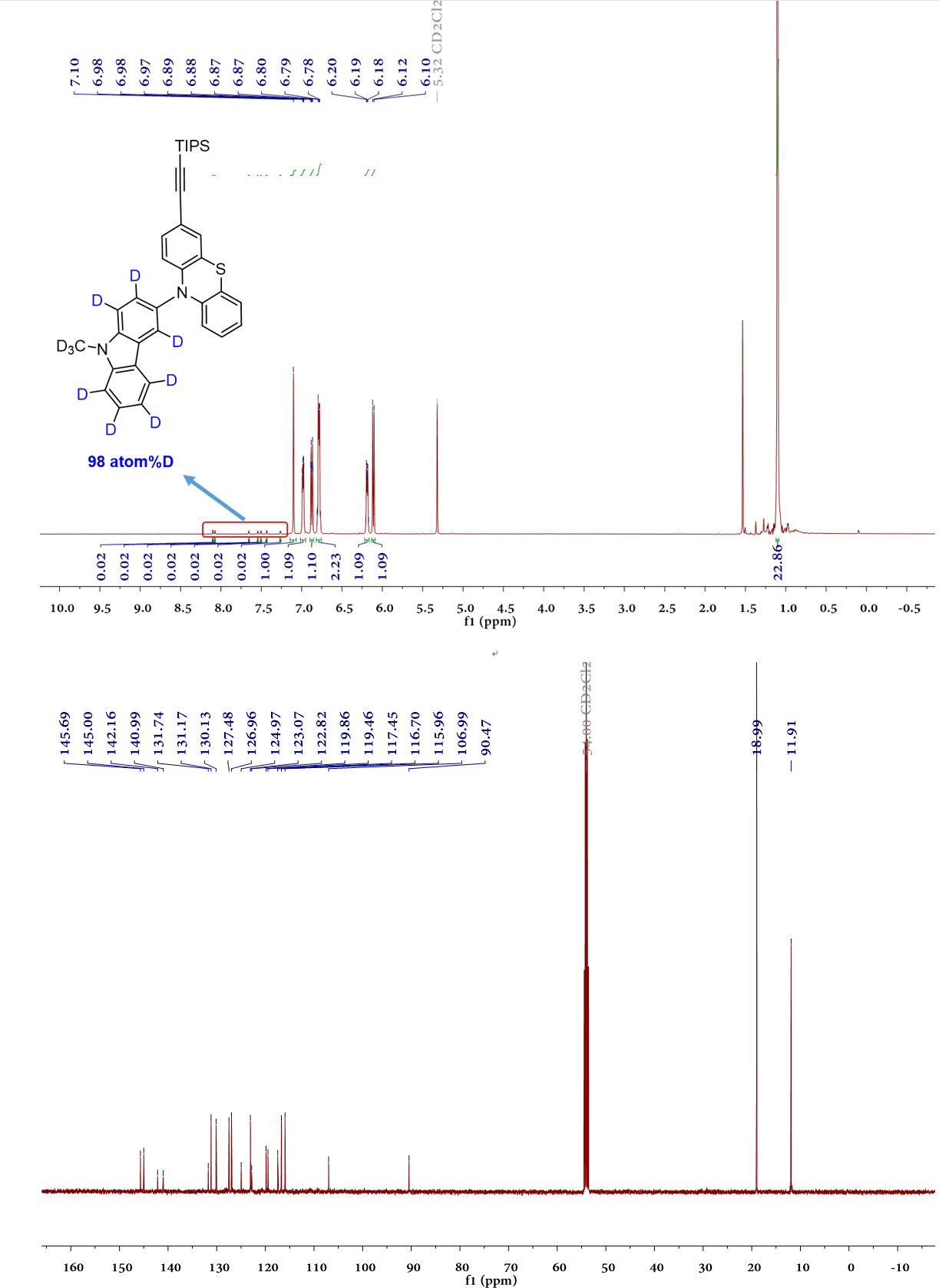


^1^H and ^13^C NMR spectra of L**4**


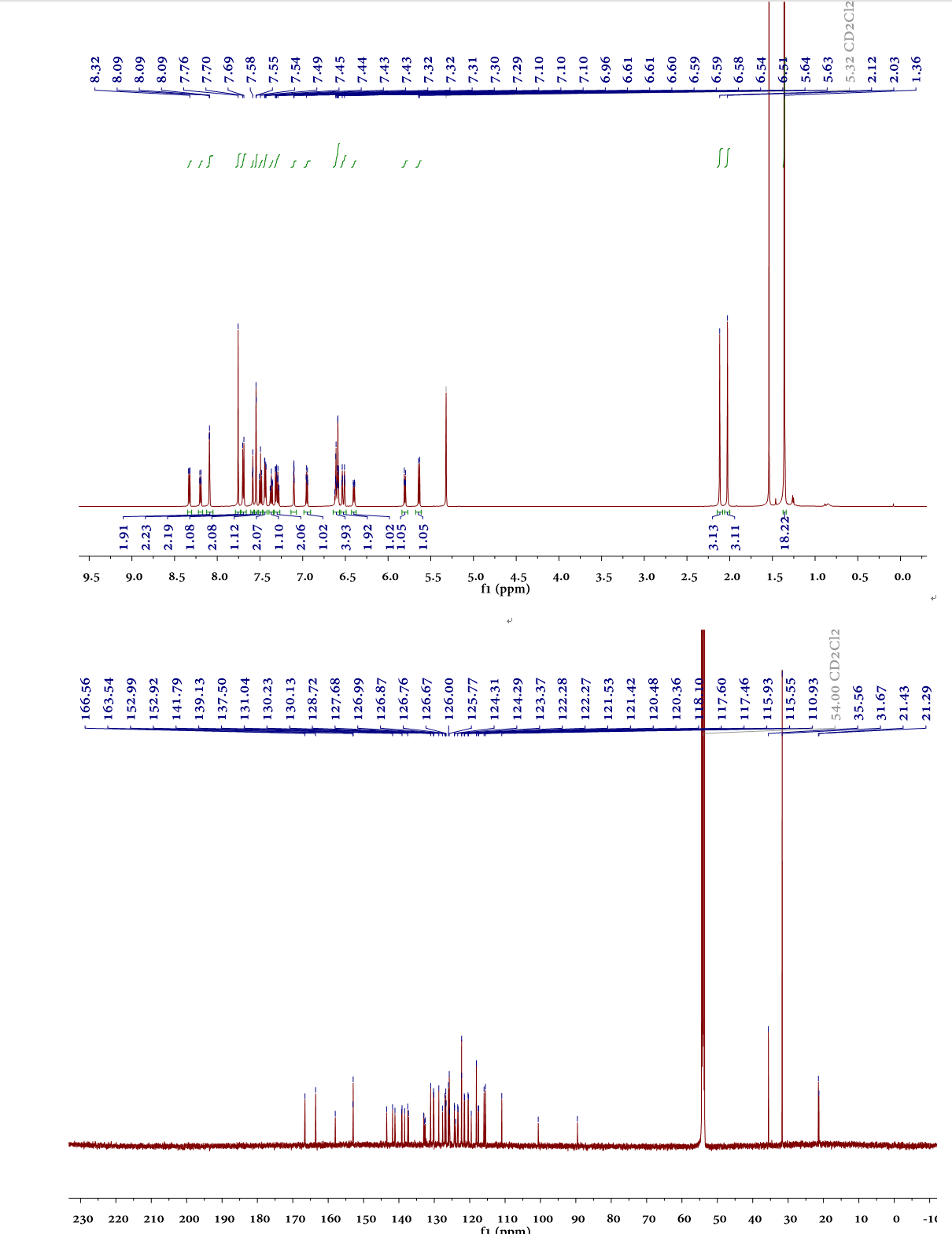


^1^H and ^13^C NMR spectra of **1**


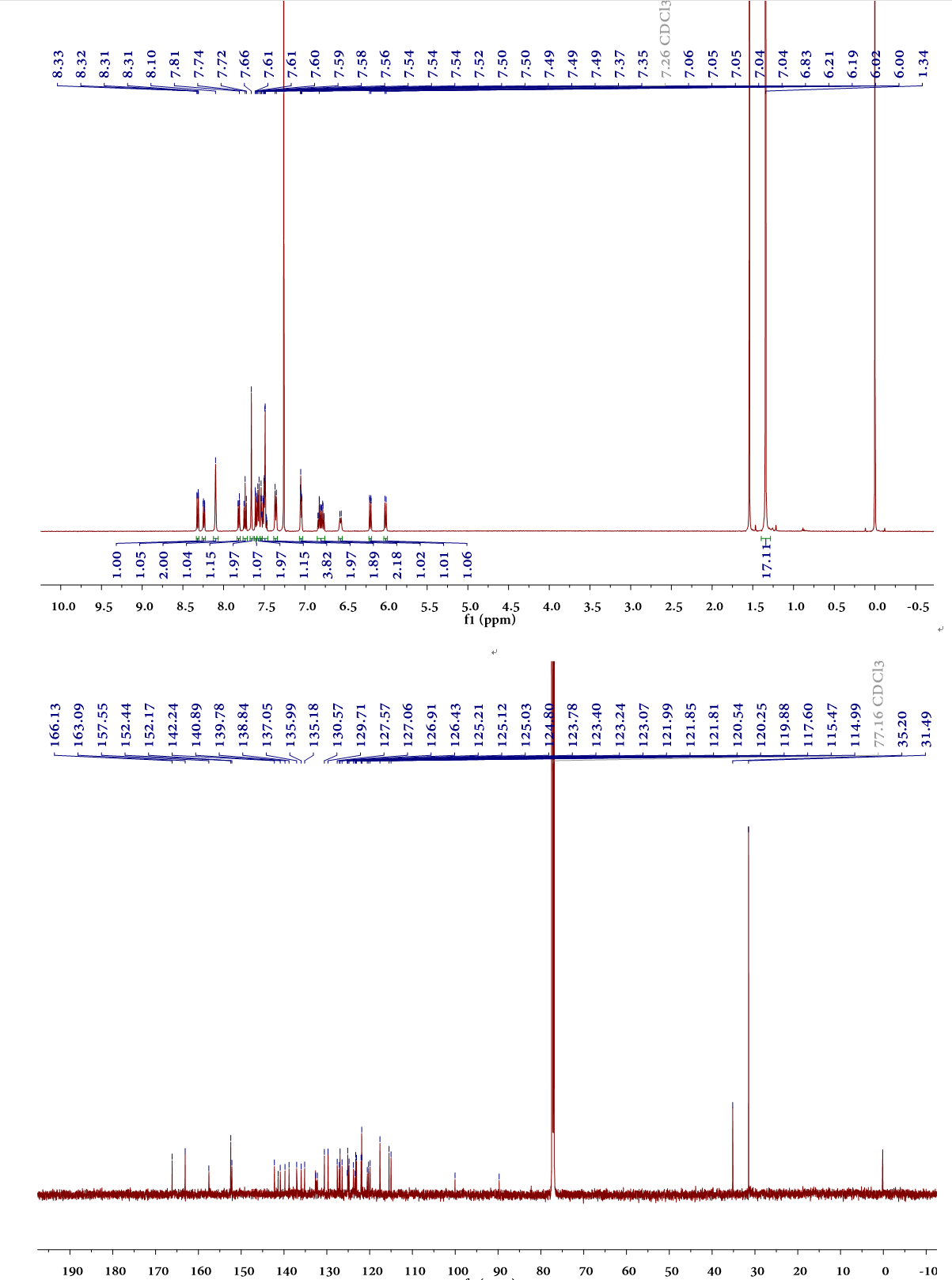


^1^H and ^13^C NMR spectra of **2**


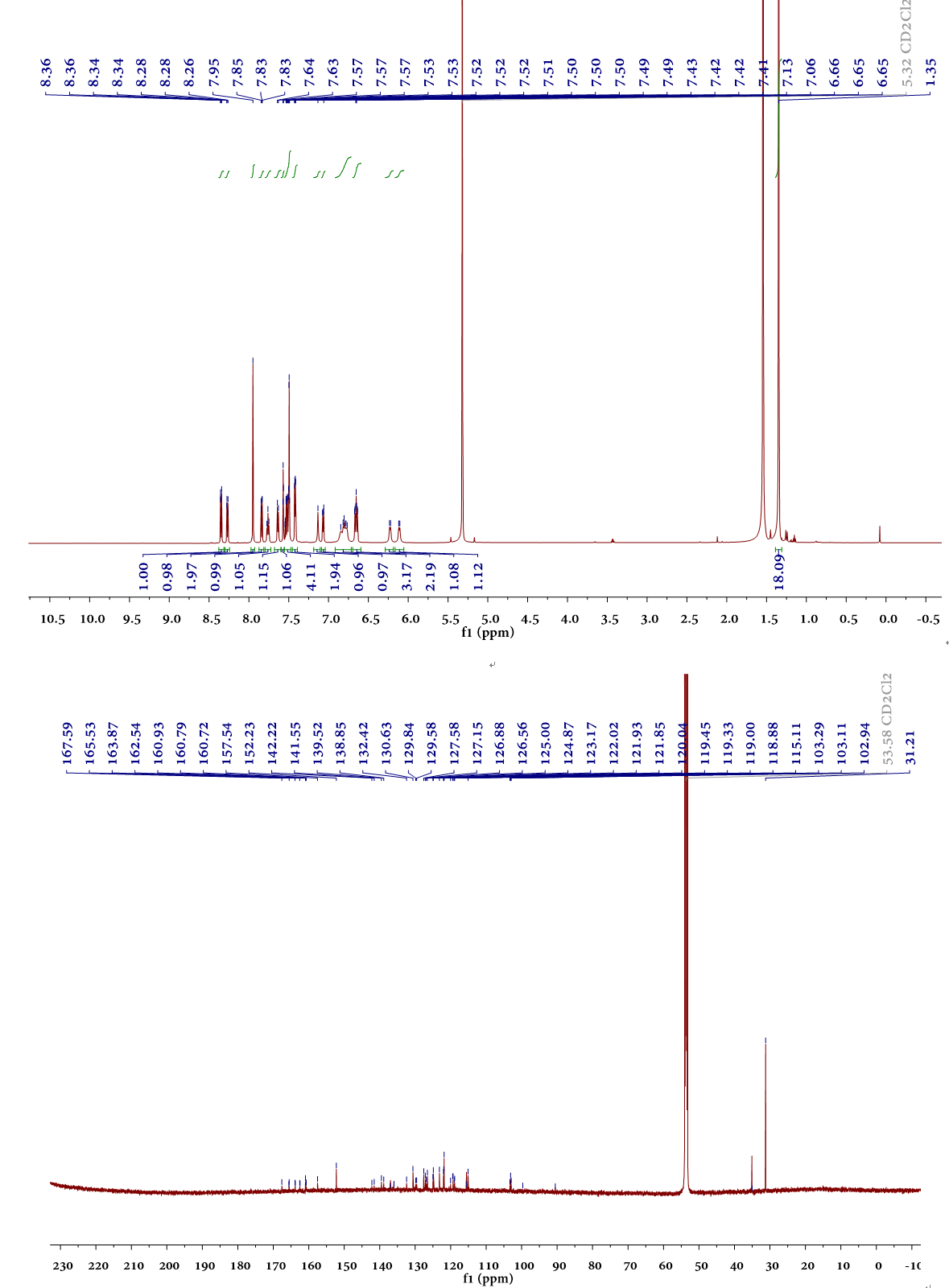


^1^H and ^13^C NMR spectra of **3**


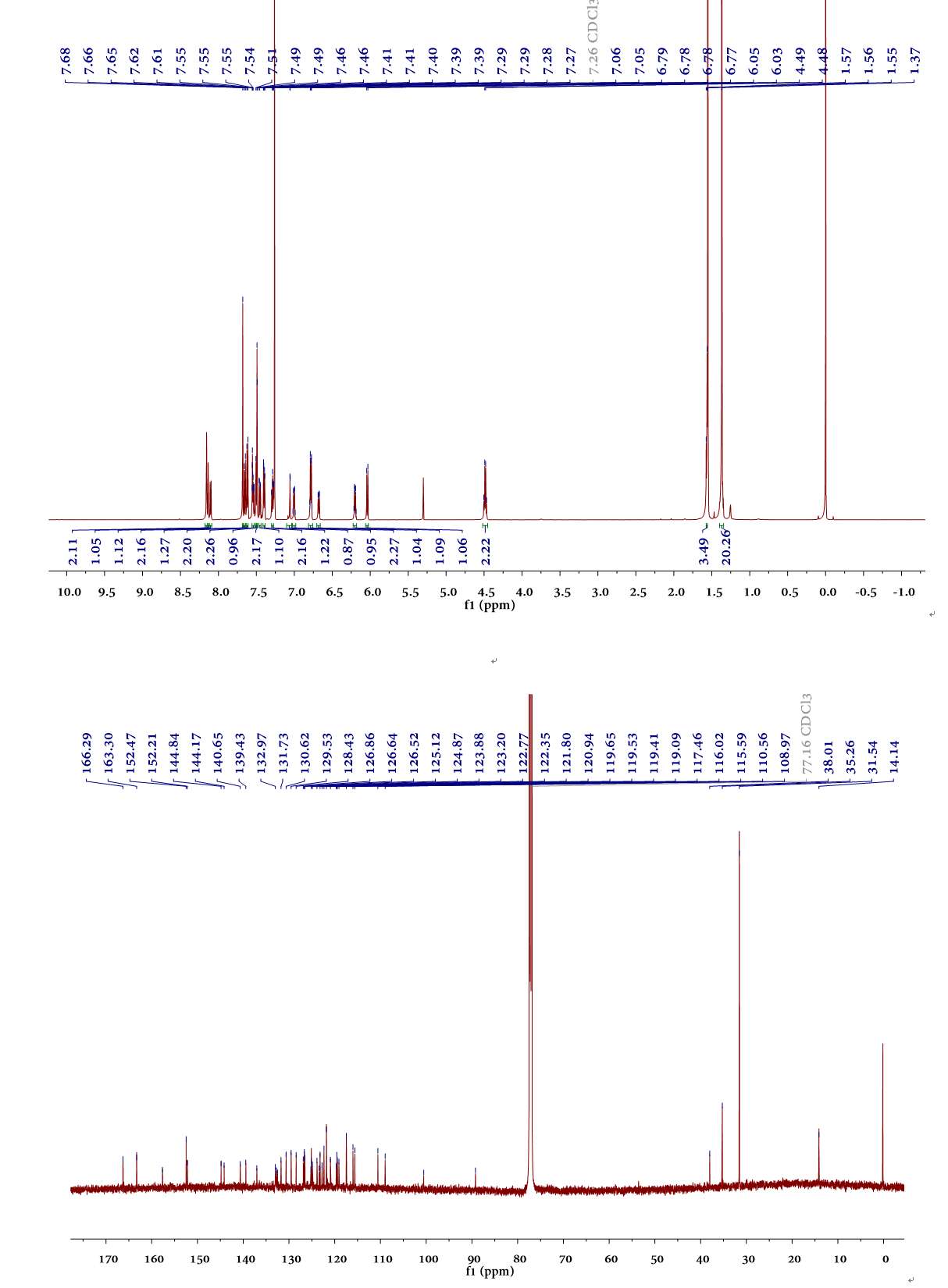


^1^H and ^13^C NMR spectra of **4**


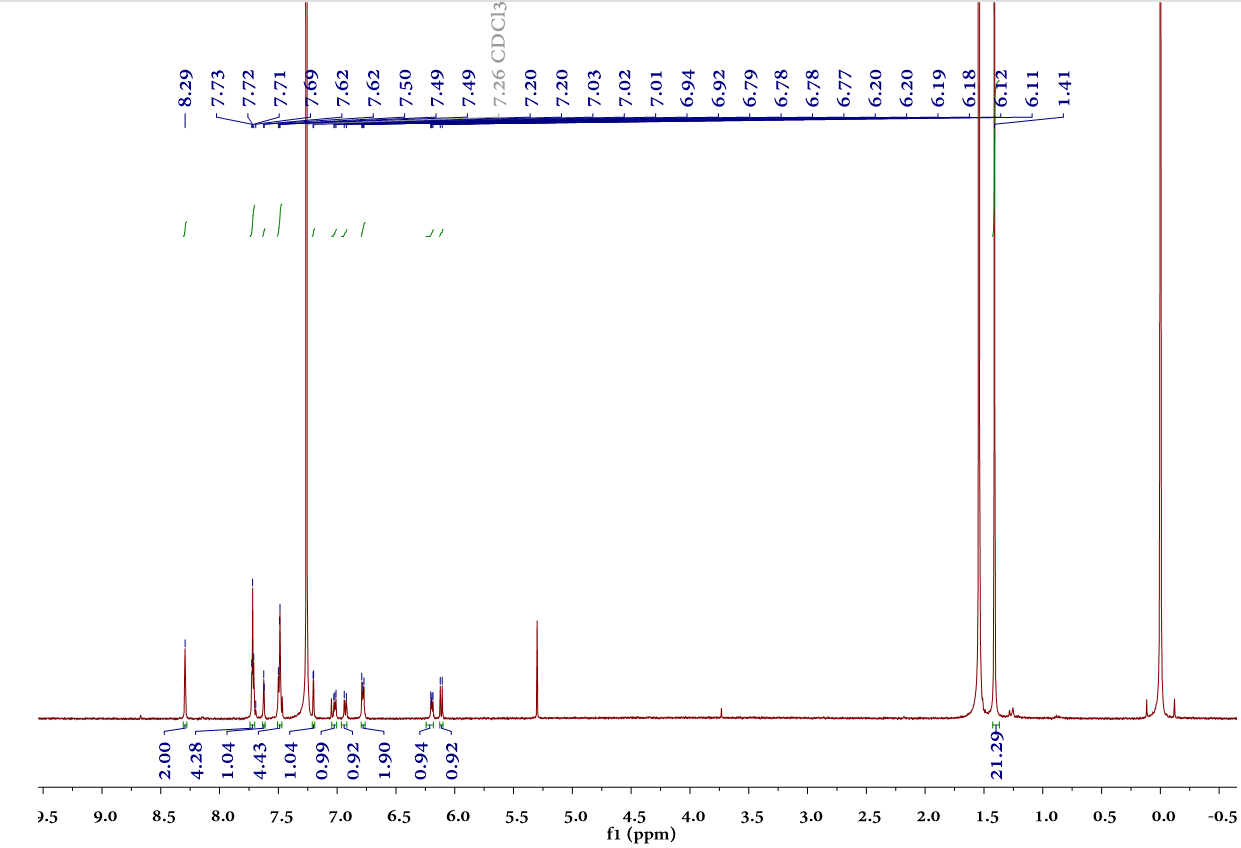


^1^H NMR spectrum of **5**

Cartesian coordinates for DFT optimized structures

**Emitter 2: semicoplanar geometry in the ground state S_0_**

C 0.45518800 0.23223200 -0.37169900

C 3.09009600 0.34459000 -0.59298500

C 1.67171300 0.28659700 -0.47212500

C 3.83783800 -0.79951000 -0.89833800

C 5.21612100 -0.73800700 -1.03606000

C 5.90456400 0.46470800 -0.84620400

C 5.16191200 1.60583600 -0.50228800

C 3.77953900 1.55141600 -0.40556900

Au -1.50918500 0.11818300 -0.21895000

N -3.54023600 -0.00876600 -0.06673400

C -4.21776300 1.11728000 0.23893500

C -5.60052000 1.04392600 0.34485600

C -6.25315500 -0.17892500 0.13545300

C -5.49158200 -1.31330900 -0.17835600

C -4.11038600 -1.21303900 -0.27911000

C -3.14536100 -2.27735100 -0.60513000

C -3.35444600 2.29579500 0.42845000

C -3.86614100 3.55152600 0.75754200

C -3.00932100 4.63228500 0.92617200

C -1.64044600 4.45169000 0.76501900

C -1.11658300 3.19870100 0.43512300

C -1.77097800 -1.91662200 -0.64628900

C -1.95480000 2.10864000 0.26289200

C -0.83844100 -2.89590400 -0.94916100

C -1.24555600 -4.20730500 -1.20922100

C -2.59027900 -4.55757600 -1.16940500

C -3.54027200 -3.58979000 -0.86683400

C -7.72068400 -0.27169100 0.24248400

C -8.31626600 -1.41578500 0.79114300

C -9.69769100 -1.52050700 0.90286500

C -10.47537600 -0.44941800 0.43772700

C -9.92118800 0.70139500 -0.11791300

C -8.52680400 0.77328400 -0.20214000

C -10.76625400 1.86295500 -0.63635400

C -10.38700700 -2.74042900 1.50969100

C -9.39019800 -3.80418500 1.96434100

C -11.20973800 -2.30786100 2.72884100

C -11.31319800 -3.37296200 0.46439100

C -12.26283900 1.62225300 -0.45233000

C -10.39336500 3.14167300 0.12273900

N 7.30571600 0.54208900 -0.98580700

C 7.94524500 1.72312000 -1.42968900

C 9.13221500 1.67450300 -2.16552400

C 9.75066200 2.84487100 -2.59314400

C 9.18397500 4.08421300 -2.32215300

C 7.99763300 4.14338200 -1.59845200

C 7.39581500 2.97879400 -1.13248000

S 5.99828400 3.10356300 -0.04518200

C -10.49551300 2.05901200 -2.13253200

C 9.58727100 -3.01199900 -0.65712900

C 9.01872900 -2.66950900 -1.87586300

C 9.40421100 -2.18250200 0.45307000

C 8.64106800 -1.00999200 0.30729000

C 8.06768800 -0.66154200 -0.91439700

C 8.26245400 -1.50036400 -2.00632200

C 9.89361900 -2.33310300 1.80713400

C 9.48395700 -1.27083900 2.63554700

S 8.50144700 -0.07741200 1.78468300

C 10.67973400 -3.35471000 2.34940100

C 11.04111900 -3.30511600 3.68630500

C 10.62552000 -2.24176400 4.49646400

C 9.84466300 -1.21697200 3.97984400

C -0.18812500 -5.22129500 -1.51747700

F -0.69650400 -6.42263800 -1.81137600

F 0.64604100 -5.38259700 -0.48088100

F 0.56477900 -4.83885100 -2.55784300

C -0.68299600 5.59160900 0.92581400

F -0.03661100 5.84656800 -0.22008200

F 0.25469400 5.31389100 1.84117500

F -1.28829700 6.72274900 1.30307000

H 3.32240300 -1.74774600 -1.04192500

H 5.76848600 -1.64049600 -1.28596300

H 3.22263600 2.45392900 -0.15482000

H -6.17740000 1.92402500 0.61894100

H -5.98812200 -2.26129100 -0.37092500

H -4.93775500 3.69974000 0.88717600

H -3.40513200 5.61124900 1.18344700

H -0.03851500 3.08109500 0.31535600

H 0.22248900 -2.64374600 -0.98782200

H -2.89532500 -5.58038800 -1.37439200

H -4.59255700 -3.87076900 -0.83871900

H -7.67339500 -2.21124000 1.16294900

H -11.55813200 -0.52644500 0.51776600

H -8.05632700 1.64606500 -0.65603100

H -9.93369200 -4.65847800 2.38963200

H -8.78468600 -4.18245100 1.12866600

H -8.71146400 -3.42586400 2.74143400

H -11.70969800 -3.17875400 3.17619900

H -11.98641600 -1.57785400 2.46614100

H -10.56615300 -1.85410200 3.49515600

H -10.74503000 -3.69535500 -0.41921300

H -11.81415100 -4.25521000 0.88764300

H -12.09355300 -2.67822600 0.12770300

H -12.82257700 2.48585100 -0.83576200

H -12.60682700 0.73593400 -1.00341100

H -12.53155500 1.49841700 0.60603500

H -10.99671500 3.98640700 -0.23890800

H -9.33682600 3.40878900 -0.01128900

H -10.57875900 3.02728300 1.19973600

H 9.58194400 0.71180400 -2.39655100

H 10.68083800 2.77729400 -3.15438700

H 9.65942300 5.00022600 -2.66629700

H 7.53618100 5.10276900 -1.36676200

H -10.75804300 1.15567500 -2.70027400

H -11.09846900 2.89244800 -2.52034200

H -9.44149500 2.28949800 -2.33566600

H 10.17447100 -3.92509600 -0.56509000

H 9.16026700 -3.31510700 -2.74007800

H 7.81376400 -1.22844200 -2.96185400

H 11.00448700 -4.18428400 1.72184800

H 11.65266000 -4.09904200 4.11069400

H 10.91666300 -2.21621800 5.54501400

H 9.52105500 -0.39054800 4.61009500

**Emitter 2: twisted geometry in the ground state S_0_**

C -0.45305800 0.47175400 0.11020000

C -3.08667000 0.72453200 0.20571700

C -1.66822500 0.59231200 0.14902000

C -3.82989400 0.09570200 1.21224500

C -5.20723800 0.24088700 1.28118800

C -5.90021300 0.99088100 0.32580500

C -5.16237300 1.58825400 -0.70814000

C -3.77967500 1.48328300 -0.74837600

Au 1.50956900 0.25165800 0.05731300

N 3.53661200 0.01470100 0.00965700

C 4.29574100 1.12784200 -0.05852100

C 5.67644000 0.98166800 -0.07964400

C 6.24366200 -0.29929000 -0.03025200

C 5.40010300 -1.41693500 0.03760200

C 4.02244300 -1.24311200 0.05890700

C 2.97868600 -2.27942500 0.14109800

C 3.51607200 2.37659600 -0.10563400

C 4.12091800 3.63124400 -0.18847000

C 3.34141300 4.78064500 -0.22649700

C 1.95648800 4.66978300 -0.18105400

C 1.33957500 3.41827900 -0.09846500

C 1.62727400 -1.83864400 0.16025400

C 2.10002200 2.26017900 -0.06124700

C 0.62151900 -2.78888300 0.23701800

C 0.93483100 -4.14962000 0.29502500

C 2.25699100 -4.57856700 0.27588500

C 3.27929900 -3.64079100 0.19821200

C 7.70802300 -0.46894400 -0.04551200

C 8.28690500 -1.53680000 -0.74477200

C 9.66572800 -1.71071300 -0.77187300

C 10.45593000 -0.79143400 -0.06537300

C 9.91753100 0.27895500 0.64496500

C 8.52675700 0.42754400 0.63670000

C 10.77469600 1.27405100 1.42410100

C 10.33985800 -2.84697300 -1.53736600

C 9.33122200 -3.74512700 -2.24998800

C 11.28368900 -2.26093600 -2.59386900

C 11.14089400 -3.71426300 -0.55915200

C 12.26682200 0.96911300 1.31476400

C 10.53674900 2.68657900 0.87781600

N -7.30168100 1.14418300 0.38289600

C -7.94268700 2.31026700 -0.09531000

C -9.12707000 2.77806000 0.48024400

C -9.74738800 3.92505100 -0.00426700

C -9.18534200 4.64484600 -1.05146700

C -8.00158400 4.19314500 -1.62560200

C -7.39805800 3.02434400 -1.17283100

S -6.00571200 2.37170300 -2.05973200

C 10.38638100 1.22655900 2.90650200

C -9.56617000 -1.66458600 2.60352200

C -8.99874300 -0.57418700 3.24760900

C -9.38716800 -1.82940100 1.22709900

C -8.62964200 -0.87737600 0.52083900

C -8.05794100 0.21988300 1.16275200

C -8.24787500 0.36449700 2.53282200

C -9.87617100 -2.87451600 0.35275800

C -9.47102300 -2.67612300 -0.98106500

S -8.49413600 -1.22221000 -1.19212000

C -10.65779200 -3.99058000 0.66751100

C -11.01880900 -4.87870500 -0.33320800

C -10.60738500 -4.66705200 -1.65454000

C -9.83128600 -3.56623600 -1.99000500

C -0.19601800 -5.12685000 0.38689000

F 0.22254700 -6.39703400 0.40157900

F -1.03938700 -4.99416200 -0.64492400

F -0.91674600 -4.93037700 1.49960700

C 1.08068500 5.88415800 -0.20562700

F 0.36800800 5.99051100 0.92404500

F 0.19867900 5.82827700 -1.21205800

F 1.77734300 7.01712100 -0.34614400

H -3.31230400 -0.50355300 1.95894000

H -5.75541900 -0.24690900 2.08350000

H -3.22756200 1.97362800 -1.54939000

H 6.31993400 1.85434300 -0.16246300

H 5.82860900 -2.41396400 0.10675600

H 5.20576100 3.72507500 -0.22573900

H 3.80965400 5.75921100 -0.29225200

H 0.25112200 3.35647500 -0.06426500

H -0.42357400 -2.47648500 0.25100900

H 2.48807900 -5.63957600 0.32080100

H 4.31326700 -3.98390700 0.18307900

H 7.63659500 -2.21092700 -1.29897800

H 11.53607200 -0.92509700 -0.07739400

H 8.06516900 1.23761500 1.20228500

H 9.86360700 -4.54468700 -2.78230400

H 8.63836600 -4.22451300 -1.54420000

H 8.74118900 -3.19104000 -2.99328300

H 11.77421400 -3.07010400 -3.15342200

H 12.07196100 -1.64135500 -2.14689800

H 10.73119100 -1.63780400 -3.31066700

H 10.48464300 -4.14966500 0.20715700

H 11.62917600 -4.53884300 -1.09778900

H 11.92532900 -3.14337300 -0.04543900

H 12.83553100 1.71320000 1.88842200

H 12.51439700 -0.02063300 1.72293500

H 12.62004700 1.01388500 0.27511600

H 11.14905000 3.41413400 1.42927000

H 9.48780000 2.99620100 0.97483100

H 10.80984000 2.74754800 -0.18477800

H -9.57392100 2.23295900 1.30818200

H -10.67548700 4.25921900 0.45590200

H -9.66226500 5.54893500 -1.42393300

H -7.54377300 4.73510700 -2.45248500

H 10.55005500 0.22297300 3.32283800

H 10.99757100 1.93737000 3.48069800

H 9.33265200 1.48958700 3.06724900

H -10.14915300 -2.39117600 3.16847400

H -9.13689800 -0.44590800 4.31915000

H -7.79961000 1.22158800 3.03556900

H -10.97933200 -4.15881100 1.69508900

H -11.62671500 -5.74819700 -0.09061900

H -10.89797700 -5.37334500 -2.43017100

H -9.51091000 -3.40222900 -3.01733100

**Emitter 2: orthogonal geometry in the ground state S_0_**

C 0.45566600 0.46331100 0.03292100

C 3.08840800 0.72865900 0.12290900

C 1.67044600 0.58612400 0.07078400

C 3.85594300 0.80777700 -1.04533700

C 5.23337000 0.96103700 -0.98646200

C 5.90029100 1.01272700 0.24116200

C 5.13653700 0.89948900 1.41310700

C 3.75519300 0.78728100 1.35490700

Au -1.50864400 0.25116400 -0.01413900

N -3.53732400 0.02625600 -0.05389500

C -4.28960900 1.14317400 -0.13366300

C -5.67116100 1.00590700 -0.14536600

C -6.24718300 -0.27039400 -0.07753100

C -5.41025800 -1.39228200 0.00299500

C -4.03135500 -1.22741200 0.01572400

C -2.99357100 -2.27008000 0.09389100

C -3.50272600 2.38712500 -0.18772600

C -4.10046200 3.64461700 -0.27831700

C -3.31439100 4.78913600 -0.32650500

C -1.93003400 4.67048500 -0.28411300

C -1.32013500 3.41602200 -0.19346700

C -1.63927600 -1.83781200 0.10225900

C -2.08735900 2.26280400 -0.14488800

C -0.63883800 -2.79367500 0.17869600

C -0.96028100 -4.15223800 0.24373000

C -2.28513200 -4.57299900 0.23387000

C -3.30216800 -3.62925400 0.15893000

C -7.71286200 -0.42901800 -0.08790000

C -8.31977100 -1.42585600 0.68838900

C -9.70058200 -1.58469000 0.69785700

C -10.46455100 -0.72296800 -0.10350500

C -9.89827200 0.27519900 -0.89299400

C -8.50634500 0.41006200 -0.86649900

C -10.72735500 1.20863400 -1.77261100

C -10.40401300 -2.64370200 1.54364400

C -9.42082600 -3.49206800 2.34712800

C -11.35676400 -1.95854100 2.53015600

C -11.20108600 -3.57997000 0.62773600

C -12.22391400 0.91989600 -1.68053500

C -10.49501900 2.65879200 -1.33247100

N 7.30151600 1.16707800 0.31558700

C 7.91669800 1.84675100 1.39189400

C 9.10466200 2.56217100 1.21847700

C 9.69873000 3.22207500 2.28925700

C 9.10665500 3.20746600 3.54609600

C 7.91937700 2.50582900 3.72767500

C 7.34202900 1.80914800 2.67087900

S 5.94557000 0.76004000 2.98740800

C -10.29993500 1.04342100 -3.23552700

C 9.64658700 0.13059400 -3.05657800

C 9.07886100 1.39432900 -2.97517500

C 9.44137800 -0.79144200 -2.02617700

C 8.65747000 -0.41263500 -0.92129600

C 8.08497100 0.85525700 -0.83433500

C 8.30144600 1.75702800 -1.87055800

C 9.92520800 -2.14947400 -1.89375600

C 9.49054500 -2.74758900 -0.69537700

S 8.49127800 -1.67339500 0.28457000

C 10.72688900 -2.88744500 -2.77040100

C 11.07906000 -4.18814200 -2.44683900

C 10.63872800 -4.76792900 -1.25106300

C 9.84199700 -4.05460700 -0.36620000

C 0.16482500 -5.13649900 0.33329200

F -0.26193100 -6.40409300 0.34697800

F 0.88823600 -4.94579600 1.44461400

F 1.00697600 -5.00802300 -0.70026000

C -1.04715100 5.87934700 -0.32272100

F -0.17333500 5.81171800 -1.33590300

F -0.32501800 5.98863500 0.80037800

F -1.73799600 7.01561200 -0.46546300

H 3.35912400 0.75945300 -2.01246500

H 5.80199000 1.03113400 -1.91062700

H 3.18287700 0.71817600 2.27930100

H -6.30934200 1.88581300 -0.17349100

H -5.84350000 -2.38929200 0.02839000

H -5.18482300 3.74473400 -0.31306900

H -3.77715800 5.76992400 -0.39765500

H -0.23194700 3.34803000 -0.16167400

H 0.40817400 -2.48762600 0.18697500

H -2.52246800 -5.63240200 0.28375100

H -4.33828000 -3.96604300 0.15096900

H -7.69050600 -2.05448500 1.31547500

H -11.54622100 -0.84396200 -0.10341600

H -8.02362600 1.16189400 -1.49166300

H -9.97384100 -4.23877600 2.93248200

H -8.72147900 -4.03599400 1.69677200

H -8.83761400 -2.88605600 3.05438200

H -11.86735200 -2.71134500 3.14746900

H -12.12895000 -1.36939800 2.01852700

H -10.80744800 -1.28384400 3.20127200

H -10.53896300 -4.08602400 -0.08850700

H -11.70879700 -4.35061000 1.22506800

H -11.96980100 -3.04491300 0.05516600

H -12.77174200 1.61693500 -2.32874300

H -12.46623300 -0.09902400 -2.01308200

H -12.60553400 1.05133100 -0.65840600

H -11.08792600 3.34355800 -1.95534500

H -9.44183300 2.95510200 -1.42526000

H -10.79560600 2.80412200 -0.28563200

H 9.57469400 2.59374900 0.23841500

H 10.63022300 3.76145700 2.12736900

H 9.56306600 3.73339700 4.38197000

H 7.43862100 2.47217300 4.70483300

H -10.45730500 0.01075000 -3.57625600

H -10.89225300 1.70856900 -3.87986500

H -9.24130800 1.29065200 -3.38858900

H 10.25031200 -0.14303700 -3.92121600

H 9.23781600 2.11205200 -3.77728800

H 7.85329700 2.74848400 -1.80362400

H 11.07123400 -2.43970600 -3.70235500

H 11.70269700 -4.76432800 -3.12765200

H 10.92291900 -5.79074800 -1.01032300

H 9.49889300 -4.50570400 0.56315100

**Emitter 2: semicoplanar geometry in the S_1_ excited state**

C 0.49460000 0.51427200 -0.24291300

C 3.11968900 0.73428300 -0.40199600

C 1.71202000 0.62647700 -0.31150400

C 3.91632500 -0.39551500 -0.71528900

C 5.28334900 -0.30751600 -0.81548600

C 5.94999800 0.91732500 -0.60816600

C 5.15738300 2.04742900 -0.28727800

C 3.77103800 1.94685800 -0.18995200

Au -1.48583100 0.29218200 -0.14592900

N -3.46989300 0.04852700 -0.05440400

C -4.26180600 1.15667200 0.15344100

C -5.62675700 0.98956200 0.21041000

C -6.21464300 -0.29110500 0.06857400

C -5.34898900 -1.39152500 -0.13833800

C -3.98407700 -1.22206700 -0.20373800

C -2.95552800 -2.23074600 -0.42297500

C -3.49392800 2.38892800 0.29171300

C -4.07536600 3.64321800 0.51593000

C -3.28800300 4.77793800 0.63325700

C -1.90084000 4.67009200 0.52589100

C -1.30566000 3.42521200 0.30349000

C -1.60427700 -1.77656300 -0.44358600

C -2.07386100 2.27693800 0.18362700

C -0.58602300 -2.70055400 -0.64749700

C -0.87551700 -4.05132300 -0.83007500

C -2.20248400 -4.49713300 -0.80889600

C -3.23030900 -3.59479700 -0.60698800

C -7.66666200 -0.46844500 0.13215500

C -8.22879900 -1.68333800 0.56817900

C -9.60273700 -1.87077800 0.63603000

C -10.43778300 -0.80622400 0.26052500

C -9.92693900 0.41472400 -0.17601800

C -8.53890800 0.56184300 -0.23572900

C -10.82165800 1.57754000 -0.60399600

C -10.23197600 -3.17850400 1.11426700

C -9.18791900 -4.23332800 1.47462100

C -11.08355800 -2.91233400 2.36079700

C -11.11868000 -3.75408800 0.00378000

C -12.30779700 1.25871700 -0.45447900

C -10.51420200 2.80599600 0.25983400

N 7.33462200 0.98431600 -0.71904700

C 8.10513100 2.12737700 -0.51480800

C 9.50713700 2.06076300 -0.63402100

C 10.28741400 3.18035300 -0.43603600

C 9.70641900 4.41264300 -0.11365600

C 8.33587000 4.50191500 0.00975900

C 7.52815200 3.37416900 -0.18506200

S 5.81762100 3.63153500 0.00171400

C -10.55574400 1.91027900 -2.07665700

C 9.37420000 -2.57363200 -1.76516700

C 8.92351500 -1.70840400 -2.75205600

C 9.15626800 -2.27508300 -0.41740200

C 8.47609200 -1.08932200 -0.07772600

C 8.02947700 -0.22788600 -1.07674400

C 8.24986000 -0.53290500 -2.41173800

C 9.53415900 -3.02731000 0.76026800

C 9.12822300 -2.39026000 1.94649900

S 8.28286800 -0.86875000 1.64885900

C 10.21686800 -4.24501000 0.84270700

C 10.47873800 -4.79775300 2.08575200

C 10.06596100 -4.14929400 3.25572200

C 9.38701200 -2.94007000 3.19881300

C 0.19901400 -5.05990500 -1.04270100

F 0.03597200 -5.73157600 -2.19710200

F 0.20917500 -5.99422200 -0.07444300

F 1.42522300 -4.51935000 -1.07521900

C -1.01422700 5.86026100 0.64861800

F -0.24355700 6.02618200 -0.44223300

F -0.16076400 5.74683100 1.68350100

F -1.69200500 7.00156300 0.82559400

H 3.42377600 -1.35142200 -0.87811900

H 5.85720800 -1.19656600 -1.05809600

H 3.18315500 2.83059600 0.05401800

H -6.25989600 1.85268200 0.40795700

H -5.76752800 -2.38524200 -0.28730700

H -5.15813000 3.73819000 0.60068900

H -3.74763300 5.74800100 0.80715500

H -0.21870900 3.36295400 0.22260500

H 0.45080600 -2.36656200 -0.66522000

H -2.41903800 -5.55514000 -0.95264300

H -4.25975800 -3.95341600 -0.59259600

H -7.55740600 -2.47573100 0.89199700

H -11.51577800 -0.94543000 0.31529200

H -8.11715400 1.49344900 -0.61383400

H -9.69192900 -5.15336900 1.80153200

H -8.55315900 -4.49046700 0.61515300

H -8.53786000 -3.90255100 2.29643900

H -11.54068600 -3.84703000 2.71751400

H -11.89410400 -2.19924500 2.16168800

H -10.46764100 -2.50220500 3.17331900

H -10.52841100 -3.95814900 -0.90052500

H -11.57675900 -4.69842500 0.33266700

H -11.93036400 -3.06905600 -0.27357000

H -12.90362400 2.12709600 -0.76791600

H -12.60928500 0.40768400 -1.08095100

H -12.57474700 1.03194100 0.58721900

H -11.15355400 3.65051000 -0.03640900

H -9.46963000 3.12868000 0.16074000

H -10.70012600 2.59342900 1.32191500

H 9.97868300 1.11577000 -0.88495700

H 11.36677300 3.09617700 -0.53325100

H 10.32600100 5.29192400 0.03983000

H 7.86337600 5.45016300 0.26097700

H -10.77211300 1.04396800 -2.71734600

H -11.19587400 2.74377500 -2.40139200

H -9.51226600 2.20291400 -2.25115000

H 9.89696200 -3.48832000 -2.04069100

H 9.09179000 -1.94436700 -3.79993500

H 7.88947400 0.15177500 -3.17795400

H 10.53848000 -4.75443900 -0.06492700

H 11.00845700 -5.74554800 2.15396800

H 10.27760800 -4.59779500 4.22429700

H 9.06439800 -2.43734500 4.10836200

**Emitter 2: orthogonal geometry in the S_1_ excited state**

C 0.49424700 0.43572300 0.23289200

C 3.11273900 0.67981100 0.41510300

C 1.71342300 0.55047700 0.31677800

C 3.93131500 0.63390300 -0.74261200

C 5.29517000 0.75820300 -0.66272400

C 5.93938400 0.93661400 0.58090600

C 5.12258300 0.98047300 1.74255800

C 3.74204400 0.85458600 1.64874600

Au -1.47882400 0.24283400 0.12052300

N -3.46636400 0.04283200 0.01627700

C -4.23836300 1.17675200 -0.13101200

C -5.60549600 1.04071000 -0.20297300

C -6.22221100 -0.23085900 -0.11576200

C -5.38023000 -1.35728600 0.04339600

C -4.01162100 -1.22251500 0.10346500

C -3.00566100 -2.26504800 0.24728700

C -3.44976100 2.39972200 -0.18275800

C -4.00740400 3.67707600 -0.32421200

C -3.19981100 4.80189300 -0.36770400

C -1.81443000 4.66201000 -0.27079300

C -1.24189100 3.39487700 -0.13014300

C -1.64249400 -1.84017900 0.28992100

C -2.03234600 2.25573600 -0.08366700

C -0.64832400 -2.79742700 0.42805300

C -0.96640600 -4.15470500 0.52422200

C -2.29839300 -4.57008900 0.48254200

C -3.30683500 -3.63000900 0.34470900

C -7.67771100 -0.37222800 -0.18771600

C -8.33465700 -1.43447700 0.46174800

C -9.71425000 -1.58239800 0.40907600

C -10.45675400 -0.63609100 -0.31523100

C -9.85011300 0.43217200 -0.97315700

C -8.45945900 0.54445900 -0.89890300

C -10.64232600 1.46226500 -1.77766800

C -10.44767800 -2.72052300 1.11726300

C -9.49747500 -3.65300000 1.86573100

C -11.43627400 -2.13972800 2.13482300

C -11.21128900 -3.55632300 0.08357700

C -12.14759900 1.20776500 -1.73309800

C -10.38447500 2.86336200 -1.21175400

N 7.31807600 1.05733400 0.63523000

C 8.07309300 1.20708900 1.80340400

C 9.47580200 1.28809800 1.72520800

C 10.24024300 1.43162000 2.86505100

C 9.63826200 1.50136500 4.12557400

C 8.26409000 1.42449800 4.22488700

C 7.47472800 1.27730600 3.07833000

S 5.75538600 1.20083300 3.35047300

C -10.19837500 1.41533300 -3.24423600

C 9.43386800 0.92272100 -3.02820200

C 8.98243800 2.13429800 -2.52432500

C 9.19229100 -0.26230700 -2.32794900

C 8.48619700 -0.20881000 -1.11015900

C 8.03882500 1.01328800 -0.61405300

C 8.28412900 2.18440000 -1.31537800

C 9.56658400 -1.61919700 -2.66641200

C 9.13075300 -2.54348800 -1.70013900

S 8.26320300 -1.77833500 -0.36616500

C 10.27078700 -2.08402300 -3.78167400

C 10.52269200 -3.43995300 -3.91308600

C 10.07912300 -4.34517500 -2.94162500

C 9.37943300 -3.90728000 -1.82587700

C 0.14880600 -5.12882900 0.68267800

F -0.27050700 -6.40061300 0.71978600

F 0.84663000 -4.91367300 1.81344300

F 1.04144200 -5.03859600 -0.31987000

C -0.90763800 5.84281300 -0.30744000

F -0.01313900 5.75889400 -1.30923700

F -0.18261500 5.95453900 0.82092400

F -1.56378500 6.99978400 -0.46548600

H 3.45658200 0.49518000 -1.71045100

H 5.88706200 0.71756000 -1.57160300

H 3.13527900 0.89106900 2.55197700

H -6.22391100 1.93266000 -0.28680200

H -5.81671900 -2.35391800 0.08008800

H -5.08828200 3.79654000 -0.40126800

H -3.64160600 5.78945500 -0.47688300

H -0.15637500 3.30947400 -0.05648700

H 0.39989400 -2.49545600 0.46164000

H -2.54177800 -5.62731600 0.55683900

H -4.34381300 -3.96493700 0.31192100

H -7.73646800 -2.12923800 1.04767000

H -11.53867600 -0.74531200 -0.35853600

H -7.95930700 1.34904600 -1.43859000

H -10.07322100 -4.45310800 2.35121400

H -8.77431500 -4.12909900 1.18891600

H -8.93839600 -3.12440600 2.65028800

H -11.96911500 -2.94966800 2.65441200

H -12.18862400 -1.49725400 1.65905900

H -10.91046200 -1.53827700 2.88948500

H -10.52136100 -3.98935400 -0.65418300

H -11.74391700 -4.38167200 0.57853000

H -11.95391500 -2.95984200 -0.46215300

H -12.66860000 1.97788500 -2.31863000

H -12.41069100 0.23125400 -2.16306900

H -12.53901300 1.24932200 -0.70698200

H -10.95083400 3.61506200 -1.78095900

H -9.32297000 3.13872400 -1.26107700

H -10.69814400 2.92390100 -0.16021000

H 9.96272400 1.23611300 0.75641900

H 11.32177600 1.48895900 2.77293600

H 10.24341900 1.61409500 5.02101900

H 7.77576300 1.47586100 5.19676400

H -10.37589700 0.41948800 -3.67388000

H -10.76324700 2.14915500 -3.83790300

H -9.13079500 1.64350700 -3.35895800

H 9.97514600 0.89400700 -3.97266300

H 9.16945300 3.05435200 -3.07252000

H 7.92375700 3.12932100 -0.91163700

H 10.61726900 -1.38360800 -4.54074500

H 11.06909800 -3.80559300 -4.77987100

H 10.28339800 -5.40727600 -3.06093600

H 9.03349400 -4.61152000 -1.07191500

**Emitter 2: semicoplanar geometry in the T_1_ excited state**

C 0.48942100 0.49827100 -0.24305800

C 3.10918300 0.72358800 -0.40416500

C 1.71043700 0.61378600 -0.31371100

C 3.91272800 -0.41413400 -0.69855500

C 5.27716600 -0.32236300 -0.79938200

C 5.94262300 0.90965700 -0.61176300

C 5.14306100 2.04783300 -0.30772700

C 3.76183400 1.94498300 -0.21140700

Au -1.47982900 0.27896600 -0.14310800

N -3.46232900 0.03923900 -0.04435700

C -4.24837500 1.14671700 0.17865800

C -5.61533900 0.98344000 0.23688600

C -6.20484500 -0.29185800 0.07866200

C -5.34652300 -1.39230500 -0.14317000

C -3.97964300 -1.22634300 -0.20836900

C -2.95498900 -2.23608500 -0.44136400

C -3.47629000 2.37459000 0.32821200

C -4.05388100 3.62764500 0.57400800

C -3.26215100 4.75620100 0.70114700

C -1.87309000 4.64609300 0.58845900

C -1.28359700 3.40530600 0.34158200

C -1.60331100 -1.78489900 -0.46369800

C -2.05898700 2.26034900 0.21101700

C -0.58823900 -2.71073200 -0.67928400

C -0.88335300 -4.05804600 -0.87181400

C -2.21149000 -4.50098700 -0.84905400

C -3.23566500 -3.59753700 -0.63541300

C -7.65968600 -0.46500800 0.14064200

C -8.22342900 -1.67137700 0.59408800

C -9.59842000 -1.85408700 0.66145000

C -10.42817800 -0.79322100 0.26536000

C -9.91426000 0.41940300 -0.18968300

C -8.52525200 0.56208200 -0.24668900

C -10.80485200 1.57712500 -0.63879600

C -10.23318700 -3.15131200 1.16013000

C -9.19312600 -4.20249500 1.54204600

C -11.08749300 -2.86036900 2.39929200

C -11.11821200 -3.74327700 0.05698000

C -12.29207900 1.26367500 -0.48917200

C -10.49703600 2.81793500 0.20710700

N 7.32454700 0.97860000 -0.72353400

C 8.09668500 2.12599900 -0.53311200

C 9.49849900 2.05761800 -0.65012900

C 10.27991600 3.17915700 -0.46436200

C 9.69809900 4.41437700 -0.15644700

C 8.32657300 4.50438200 -0.03632800

C 7.51884900 3.37526800 -0.21948100

S 5.80522200 3.63585000 -0.04248700

C -10.53282700 1.88605300 -2.11553300

C 9.37235800 -2.58544000 -1.73389100

C 8.91840600 -1.73157800 -2.72908800

C 9.15443500 -2.27348900 -0.38913700

C 8.47147300 -1.08603700 -0.06132400

C 8.02137000 -0.23570800 -1.06830600

C 8.24175800 -0.55453200 -2.40004300

C 9.53494200 -3.01271300 0.79589700

C 9.12805200 -2.36449400 1.97582100

S 8.27862700 -0.84843300 1.66325400

C 10.22079100 -4.22777100 0.89036700

C 10.48511900 -4.76711400 2.13879200

C 10.07159900 -4.10771900 3.30237800

C 9.38938000 -2.90096600 3.23345100

C 0.18770900 -5.06880200 -1.09621200

F 0.01684300 -5.73091600 -2.25455500

F 0.19762000 -6.00911400 -0.13427600

F 1.41492800 -4.53171000 -1.12966700

C -1.02018500 5.86674300 0.64903100

F -0.79075500 6.38166100 -0.57354000

F 0.18706700 5.61939500 1.18217000

F -1.57832200 6.84738200 1.37423600

H 3.42217300 -1.37370800 -0.84519100

H 5.85431100 -1.21339500 -1.02687300

H 3.17038800 2.83034800 0.01818800

H -6.24651500 1.84530300 0.44580200

H -5.77029500 -2.38200700 -0.30333600

H -5.13552200 3.72410200 0.67027500

H -3.71719100 5.72496900 0.89597800

H -0.19860800 3.33521500 0.25812400

H 0.44950700 -2.37989700 -0.69796400

H -2.43122700 -5.55720900 -1.00029300

H -4.26604100 -3.95325700 -0.61891000

H -7.55364800 -2.45979200 0.93100000

H -11.50676700 -0.92874600 0.31883800

H -8.09830400 1.48634100 -0.63700600

H -9.70088400 -5.11471400 1.88448900

H -8.55737600 -4.47764400 0.68891000

H -8.54390900 -3.85908100 2.35935600

H -11.54830200 -3.78727400 2.77106600

H -11.89549800 -2.14871100 2.18535700

H -10.47276200 -2.43776900 3.20626900

H -10.52582800 -3.96546400 -0.84162700

H -11.58044100 -4.68003300 0.40115900

H -11.92683400 -3.06071100 -0.23502900

H -12.88488400 2.12819900 -0.81840600

H -12.59348300 0.40350400 -1.10308500

H -12.56312500 1.05433400 0.55511200

H -11.13303600 3.65930100 -0.10462100

H -9.45132000 3.13654400 0.10663700

H -10.68741800 2.62253800 1.27167700

H 9.97037700 1.10960700 -0.88948000

H 11.35943000 3.09331300 -0.55951900

H 10.31681900 5.29592300 -0.01166700

H 7.85381600 5.45569300 0.20325200

H -10.74950500 1.01065500 -2.74358700

H -11.16959100 2.71610500 -2.45513500

H -9.48805200 2.17345100 -2.29109100

H 9.89765800 -3.50140700 -2.00026200

H 9.08662800 -1.97767600 -3.77468500

H 7.87916100 0.12164500 -3.17276200

H 10.54303900 -4.74566000 -0.01228500

H 11.01748700 -5.71274300 2.21629000

H 10.28536400 -4.54557000 4.27537900

H 9.06635100 -2.38959400 4.13804000

**Emitter 2: orthogonal geometry in the T_1_ excited state**

C 0.47170600 0.49720600 0.02977000

C 3.10582100 0.74975200 0.11879800

C 1.68709700 0.61457800 0.06694600

C 3.87417000 0.82308600 -1.04933200

C 5.25259700 0.96731200 -0.99051800

C 5.91975800 1.01533200 0.23709000

C 5.15513600 0.90859600 1.40902800

C 3.77307100 0.80542800 1.35075300

Au -1.49410300 0.27642100 -0.01147400

N -3.51312000 0.03886700 -0.04595800

C -4.28668100 1.19514500 -0.12604700

C -5.70917900 1.02384400 -0.13577000

C -6.27001900 -0.24158200 -0.07760800

C -5.40869800 -1.36902900 -0.00096700

C -4.00189600 -1.19043500 0.01699000

C -2.98384800 -2.24447300 0.09581400

C -3.54838000 2.38090200 -0.17313000

C -4.15094200 3.69629400 -0.25707600

C -3.36493700 4.81097600 -0.29649400

C -1.94125200 4.70009200 -0.25310900

C -1.32012100 3.42550700 -0.17640600

C -1.62509300 -1.81677400 0.10341900

C -2.06327300 2.27652800 -0.13403700

C -0.63343900 -2.78108700 0.17992200

C -0.96711100 -4.13723300 0.24577600

C -2.29485300 -4.55150200 0.23697200

C -3.30572000 -3.60209800 0.16198000

C -7.73245900 -0.42700600 -0.08998200

C -8.32272400 -1.43982600 0.67890700

C -9.70107700 -1.62094300 0.69094700

C -10.48207500 -0.76388700 -0.09878400

C -9.93287500 0.24963600 -0.88109300

C -8.54290500 0.40312500 -0.86127800

C -10.77903600 1.17735400 -1.75062400

C -10.38448700 -2.69988600 1.52813800

C -9.38567400 -3.53945700 2.32151900

C -11.34656100 -2.04094200 2.52339500

C -11.16774300 -3.64083900 0.60513400

C -12.27121100 0.86848400 -1.65254200

C -10.56366500 2.62787500 -1.30326800

N 7.32220400 1.15945100 0.31160700

C 7.94190800 1.83677000 1.38673800

C 9.13481200 2.54369200 1.21232500

C 9.73311100 3.20143300 2.28207500

C 9.14057400 3.19322700 3.53875900

C 7.94849100 2.50002000 3.72132200

C 7.36670000 1.80529900 2.66566700

S 5.96330900 0.76588900 2.98355600

C -10.35770800 1.02680200 -3.21689100

C 9.65944000 0.10266900 -3.05972600

C 9.09999100 1.37018900 -2.97999200

C 9.44863400 -0.81642900 -2.02784100

C 8.66754300 -0.43092600 -0.92327500

C 8.10338000 0.84081000 -0.83794900

C 8.32543700 1.73959200 -1.87558300

C 9.92375800 -2.17734700 -1.89351100

C 9.48555700 -2.77093800 -0.69416100

S 8.49337800 -1.68891800 0.28439700

C 10.72055700 -2.92161100 -2.76926100

C 11.06457800 -4.22403400 -2.44392600

C 10.62087700 -4.79928500 -1.24719200

C 9.82888000 -4.07965800 -0.36317300

C 0.15162500 -5.13021500 0.33587800

F -0.28408400 -6.39437000 0.34957300

F 0.87436400 -4.94298000 1.44758200

F 0.99363900 -5.00651500 -0.69765200

C -1.06965100 5.90676500 -0.33633100

F -0.38768100 5.94074800 -1.49264500

F -0.15453500 5.92076500 0.64265800

F -1.76564800 7.04680800 -0.25287300

H 3.37709200 0.77714900 -2.01645200

H 5.82177700 1.03277900 -1.91468600

H 3.20017600 0.74108800 2.27513500

H -6.35242000 1.90102200 -0.15564300

H -5.82818600 -2.37185500 0.00649200

H -5.23480300 3.79635600 -0.28927500

H -3.81747600 5.79784300 -0.35814900

H -0.23110900 3.36847800 -0.14648500

H 0.41627700 -2.48450400 0.18794500

H -2.53748200 -5.60950800 0.28794000

H -4.34385400 -3.93177800 0.15564000

H -7.68270500 -2.06482300 1.29911800

H -11.56157600 -0.90262600 -0.09730800

H -8.07313600 1.16439500 -1.48520900

H -9.92479400 -4.30079600 2.90105300

H -8.67895600 -4.06544400 1.66436100

H -8.81081500 -2.93038300 3.03298600

H -11.84313200 -2.80827000 3.13429800

H -12.12955900 -1.45977800 2.01919300

H -10.80720000 -1.36364300 3.19997300

H -10.49870800 -4.12842600 -0.11752000

H -11.66097800 -4.42588300 1.19589200

H -11.94650100 -3.11317200 0.03936400

H -12.83187100 1.56312100 -2.29237500

H -12.50202600 -0.15103300 -1.99137700

H -12.64849700 0.98689400 -0.62722200

H -11.16919400 3.30850500 -1.91857800

H -9.51499300 2.93845300 -1.40019100

H -10.86005600 2.76292400 -0.25384100

H 9.60527800 2.57025200 0.23232000

H 10.66832400 3.73412800 2.11948300

H 9.60032200 3.71762000 4.37377300

H 7.46726800 2.47144400 4.69841500

H -10.50366200 -0.00578100 -3.56299900

H -10.96210000 1.68813700 -3.85401300

H -9.30314900 1.28842900 -3.37396500

H 10.26098000 -0.17625500 -3.92419900

H 9.26328300 2.08566300 -3.78324000

H 7.88383200 2.73406400 -1.80995700

H 11.06757300 -2.47731800 -3.70187800

H 11.68441700 -4.80513300 -3.12403000

H 10.89870600 -5.82350800 -1.00498800

H 9.48322400 -4.52721800 0.56694700

**Emitter 3: semicoplanar geometry in the ground state S_0_**

C 0.59108800 0.24245900 -0.40181400

C 3.22662900 0.40585400 -0.58013500

C 1.80804400 0.31497500 -0.48398300

C 3.99365400 -0.66279600 -1.06007800

C 5.37226500 -0.56184400 -1.17049000

C 6.04228200 0.60187100 -0.77835600

C 5.27970300 1.66134200 -0.26054300

C 3.89758200 1.57413900 -0.19054400

Au -1.37643200 0.12962100 -0.26724300

N -3.40593300 0.01292200 -0.12547600

C -4.06775600 1.10862400 0.31482200

C -5.45222000 1.03576900 0.42586900

C -6.11582300 -0.14761300 0.07810300

C -5.37708800 -1.24770600 -0.37582300

C -3.99271700 -1.15654700 -0.47226200

C -3.03360500 -2.17882100 -0.91727800

C -3.17955600 2.23882100 0.62499600

C -3.61264500 3.48318200 1.08954400

C -2.74260500 4.52246800 1.36914300

C -1.38993200 4.29942900 1.17184000

C -0.89322400 3.08596100 0.71019300

C -1.65097600 -1.83585900 -0.92536300

C -1.77931000 2.05684500 0.43636200

C -0.69978300 -2.75711300 -1.33263200

C -1.11388600 -4.02179000 -1.73397900

C -2.44617100 -4.40028800 -1.74176200

C -3.38193200 -3.46709200 -1.33057700

C -7.58395000 -0.23568100 0.19136700

C -8.19012300 -1.43628700 0.58639700

C -9.57128400 -1.53691600 0.70543100

C -10.33931700 -0.40132100 0.40735800

C -9.77486100 0.80758900 0.00714300

C -8.38105200 0.87102600 -0.09115400

C -10.60851600 2.04217100 -0.32921000

C -10.26971500 -2.82011700 1.14992000

C -9.28253900 -3.95048400 1.43275800

C -11.06363100 -2.55071700 2.43340600

C -11.22426900 -3.28908500 0.04564900

C -12.10581800 1.80125500 -0.15089000

C -10.20090300 3.19825500 0.59157400

N 7.44350000 0.71885200 -0.88665800

C 8.07039100 1.96560200 -1.11959500

C 9.26660400 2.05471500 -1.83653600

C 9.87290800 3.28736700 -2.05577400

C 9.28513500 4.45750000 -1.59134000

C 8.08955400 4.38100000 -0.88479000

C 7.49922900 3.14794900 -0.62670000

S 6.08712100 3.07542100 0.44616200

C -10.36097600 2.43878000 -1.78929700

C 9.77100100 -2.80897800 -1.16817100

C 9.20024900 -2.26787700 -2.31161900

C 9.57449500 -2.18709300 0.06815600

C 8.79570700 -1.01705700 0.12461000

C 8.22025100 -0.46984900 -1.02062500

C 8.42854800 -1.10378900 -2.24070000

C 10.06312300 -2.56367700 1.37774700

C 9.63749200 -1.66640100 2.37588900

S 8.64075700 -0.35622000 1.74043300

C 10.86161400 -3.65376300 1.73809500

C 11.21916500 -3.83225700 3.06498300

C 10.78765800 -2.93085900 4.04532000

C 9.99445200 -1.84191900 3.71077000

H 3.49354900 -1.58146400 -1.36239900

H 5.93964000 -1.40408700 -1.55871600

H 3.32552700 2.41648500 0.19797900

H -6.01178400 1.88135600 0.80792600

H -5.88372200 -2.15738100 -0.67539500

H -3.11493200 5.47655100 1.72965900

H 0.18001100 2.96443200 0.57128000

H 0.36100300 -2.51112400 -1.34420300

H -2.75353600 -5.39265100 -2.05737600

H -7.55492500 -2.28474700 0.83277600

H -11.42183900 -0.47392600 0.49546000

H -7.90396000 1.79228200 -0.42651400

H -9.83285600 -4.84866700 1.74378000

H -8.69575800 -4.21547800 0.54216000

H -8.58593200 -3.69428500 2.24312600

H -11.56856600 -3.46889300 2.76609100

H -11.83361800 -1.78153600 2.29002000

H -10.39924500 -2.21553300 3.24189700

H -10.67699100 -3.49265700 -0.88518100

H -11.73112600 -4.21526800 0.35221300

H -11.99978200 -2.54439000 -0.17589100

H -12.65638100 2.71823000 -0.40066400

H -12.47533200 1.00487000 -0.81182700

H -12.35837900 1.53683400 0.88547300

H -10.79566000 4.09363500 0.36103600

H -9.14226500 3.46574500 0.47741400

H -10.37079500 2.93871200 1.64569400

H 9.73342700 1.14954700 -2.21741000

H 10.81061200 3.32511700 -2.60714600

H 9.75130100 5.42371600 -1.77211100

H 7.61152600 5.28294100 -0.50396000

H -10.64886600 1.62599200 -2.47040400

H -10.95636100 3.32635300 -2.04695200

H -9.30686300 2.67933000 -1.97964700

H 10.37049500 -3.71635200 -1.23315200

H 9.35271900 -2.75175800 -3.27407500

H 7.97882900 -0.67560300 -3.13664700

H 11.19890700 -4.35759200 0.97755200

H 11.84023000 -4.67989700 3.34807200

H 11.07603800 -3.08384900 5.08372900

H 9.65858700 -1.14146600 4.47333400

F -0.20238700 -4.91105500 -2.12752300

F -0.54107300 5.29184100 1.43660400

F -4.91822800 3.71567900 1.28318800

F -4.66605200 -3.85017000 -1.34204300

**Emitter 3: twisted geometry in the ground state S_0_**

C 0.59169800 -0.51053000 0.14001800

C 3.22759500 -0.74238900 0.22809500

C 1.80792000 -0.62151100 0.17602500

C 3.96929300 -0.11180100 1.23471400

C 5.34809900 -0.24542600 1.29797500

C 6.04389100 -0.98567100 0.33701200

C 5.30726200 -1.58535500 -0.69653500

C 3.92364300 -1.49135300 -0.73153400

Au -1.37438900 -0.30499000 0.08889800

N -3.40006800 -0.08299200 0.04157700

C -4.15112800 -1.20289900 -0.07617800

C -5.53433900 -1.06234300 -0.10789200

C -6.10591400 0.21259700 -0.01058200

C -5.27722200 1.33492600 0.11295100

C -3.89603100 1.17309700 0.13581800

C -2.85313000 2.20219800 0.25996400

C -3.35131000 -2.43378000 -0.16025800

C -3.88553400 -3.71880400 -0.28329400

C -3.09710200 -4.85376300 -0.35621800

C -1.72336500 -4.68694300 -0.30097700

C -1.12749000 -3.43695200 -0.17661900

C -1.49416500 1.77520900 0.26886700

C -1.93284600 -2.31178500 -0.10671900

C -0.46727200 2.69844500 0.37987400

C -0.78232100 4.04847300 0.48337500

C -2.08816400 4.51006500 0.47850700

C -3.10037200 3.57308900 0.36565500

C -7.57203000 0.37226400 -0.03754500

C -8.14926300 1.48203400 -0.67009700

C -9.52876700 1.64688700 -0.71047800

C -10.32367100 0.67273100 -0.08798000

C -9.78771400 -0.43992300 0.55624600

C -8.39567200 -0.57551100 0.56518100

C -10.64908800 -1.49360200 1.24907100

C -10.19858200 2.82967000 -1.40637200

C -9.18424900 3.79577600 -2.01455500

C -11.09941400 2.31296700 -2.53411000

C -11.04415300 3.60744800 -0.39112200

C -12.14235300 -1.19956900 1.12559700

C -10.38115700 -2.86541900 0.61928900

N 7.44656200 -1.12761400 0.38808800

C 8.09445700 -2.28813700 -0.09463100

C 9.28306200 -2.74951600 0.47727000

C 9.90961600 -3.89131500 -0.01143200

C 9.34979500 -4.61218200 -1.05913200

C 8.16183500 -4.16688300 -1.62955700

C 7.55188700 -3.00310700 -1.17247900

S 6.15255800 -2.35722900 -2.05356200

C -10.29439700 -1.53706200 2.73998900

C 9.70194700 1.69895900 2.59549900

C 9.14370800 0.60619000 3.24357200

C 9.51672700 1.86036700 1.21948300

C 8.76224700 0.90258300 0.51773600

C 8.19966600 -0.19710200 1.16370000

C 8.39594200 -0.33834100 2.53323500

C 9.99630700 2.90685000 0.34153900

C 9.58759700 2.70371600 -0.99046500

S 8.61862600 1.24362600 -1.19536000

C 10.77258600 4.02798500 0.65147800

C 11.12496800 4.91627200 -0.35218300

C 10.71014600 4.69979700 -1.67167300

C 9.93920400 3.59393200 -2.00238000

H 3.44932900 0.47937500 1.98625400

H 5.89527200 0.24348900 2.10031500

H 3.37278400 -1.98239300 -1.53301100

H -6.16645100 -1.93283100 -0.23519900

H -5.71004900 2.32260900 0.21884200

H -3.54704700 -5.83719400 -0.45232400

H -0.04218100 -3.36295700 -0.13514100

H 0.57677600 2.38990500 0.38559900

H -2.31746500 5.56830300 0.55882400

H -7.49605900 2.20041900 -1.16133400

H -11.40477100 0.79683800 -0.11348100

H -7.93770400 -1.41993700 1.08102800

H -9.71397900 4.62876800 -2.49599400

H -8.51864400 4.22473800 -1.25249400

H -8.56514800 3.31182700 -2.78273000

H -11.58643600 3.15516900 -3.04620000

H -11.88980300 1.64916400 -2.16011900

H -10.51445100 1.75447700 -3.27798800

H -10.41976300 3.98993800 0.42820200

H -11.52709800 4.46539600 -0.88034600

H -11.83641200 2.98870900 0.04992800

H -12.71380900 -1.98661800 1.63582300

H -12.41219500 -0.24177300 1.59187200

H -12.47145600 -1.17982300 0.07726600

H -10.99709000 -3.63345600 1.10848800

H -9.33108600 -3.16950500 0.72032400

H -10.62914400 -2.86092000 -0.45116500

H 9.72807900 -2.20342400 1.30557100

H 10.84087700 -4.22066100 0.44578300

H 9.83180700 -5.51220600 -1.43485200

H 7.70580600 -4.70979400 -2.45679700

H -10.48134400 -0.56441400 3.21584800

H -10.90775800 -2.29126700 3.25344600

H -9.24069100 -1.79601300 2.90697200

H 10.28281500 2.42991200 3.15696800

H 9.28701600 0.48044900 4.31473000

H 7.95540300 -1.19757400 3.03914600

H 11.09703800 4.19992100 1.67752400

H 11.72890200 5.78957800 -0.11343800

H 10.99417800 5.40614900 -2.44967000

H 9.61643000 3.42599400 -3.02832600

F 0.20201000 4.94006800 0.59132100

F -0.95237400 -5.77145300 -0.36956400

F -5.21264800 -3.89841700 -0.33475100

F -4.35803400 4.03585000 0.36028200

**Emitter 3: orthogonal geometry in the ground state S_0_**

C -0.59461700 -0.50303800 0.03315200

C -3.22934200 -0.74855300 0.12575900

C -1.81027400 -0.61720600 0.07224100

C -3.99854500 -0.82488500 -1.04162700

C -5.37720700 -0.96614500 -0.98127800

C -6.04370500 -1.00868300 0.24699600

C -5.27800700 -0.89871300 1.41809900

C -3.89581000 -0.79802400 1.35839800

Au 1.37292800 -0.30395200 -0.01421400

N 3.40010300 -0.09308200 -0.05163700

C 4.14497300 -1.21553200 -0.18362400

C 5.52930700 -1.08455500 -0.19117800

C 6.10841400 0.18505400 -0.07064700

C 5.28582100 1.31102400 0.05985200

C 3.90345400 1.15748700 0.07248700

C 2.86605100 2.19149400 0.20142000

C 3.33847900 -2.44032600 -0.29016400

C 3.86527900 -3.72514700 -0.44370500

C 3.07027600 -4.85392200 -0.53891200

C 1.69757900 -4.68062100 -0.47817800

C 1.10878000 -3.43006200 -0.32859000

C 1.50437900 1.77317600 0.19268600

C 1.92073800 -2.31137000 -0.23532300

C 0.48229600 2.70053700 0.31238700

C 0.80497900 4.04666800 0.44120600

C 2.11376100 4.49979600 0.45577300

C 3.12106600 3.55842200 0.33591500

C 7.57591600 0.33374700 -0.07897200

C 8.19188300 1.30479600 0.72271400

C 9.57372700 1.45396000 0.73370200

C 10.33080000 0.60910900 -0.09177000

C 9.75576000 -0.36326800 -0.90657200

C 8.36301500 -0.48890700 -0.88132000

C 10.57593000 -1.27748500 -1.81427100

C 10.28576100 2.48579000 1.60570800

C 9.31006700 3.31798800 2.43486000

C 11.23758700 1.76879700 2.57026000

C 11.08547000 3.44189800 0.71294900

C 12.07556700 -1.00920600 -1.71070700

C 10.32643400 -2.73868100 -1.42289400

N -7.44600500 -1.15028700 0.32278400

C -8.06664500 -1.82084700 1.40182900

C -9.26114900 -2.52597500 1.23133400

C -9.86046900 -3.17709700 2.30455200

C -9.26739900 -3.16403700 3.56094600

C -8.07364500 -2.47273400 3.73962300

C -7.49077600 -1.78459600 2.68025700

S -6.08438800 -0.74747100 2.99262900

C 10.15300000 -1.05876800 -3.27154300

C -9.78422400 -0.10581800 -3.05166500

C -9.22620900 -1.37363900 -2.96667700

C -9.57167300 0.81764600 -2.02404100

C -8.79022100 0.43622500 -0.91832000

C -8.22746400 -0.83573200 -0.82781600

C -8.45132700 -1.73898800 -1.86117800

C -10.04545500 2.17958500 -1.89538000

C -9.60604700 2.77801600 -0.69890700

S -8.61415800 1.69931400 0.28378200

C -10.84225300 2.92068100 -2.77382800

C -11.18504600 4.22475600 -2.45385400

C -10.74018300 4.80479200 -1.25988200

C -9.94817300 4.08837100 -0.37325700

H -3.50213900 -0.78345500 -2.00931700

H -5.94718900 -1.03381500 -1.90478400

H -3.32224900 -0.73085600 2.28219500

H 6.15785100 -1.96412300 -0.25918700

H 5.72289200 2.30035400 0.12570200

H 3.51456300 -5.83752500 -0.65703900

H 0.02384000 -3.35038900 -0.28707500

H -0.56359100 2.39841100 0.30799900

H 2.34896200 5.55485600 0.55782700

H 7.56813500 1.92198100 1.36622300

H 11.41335900 0.72257900 -0.09027300

H 7.87471100 -1.22148000 -1.52451900

H 9.86948900 4.04522100 3.03853600

H 8.61168100 3.88305400 1.80190000

H 8.72528200 2.69631400 3.12706900

H 11.75417400 2.50139700 3.20676700

H 12.00493900 1.18947800 2.04034200

H 10.68627000 1.07880400 3.22400400

H 10.42369500 3.97121000 0.01343000

H 11.60023900 4.19289500 1.32910700

H 11.84849500 2.91766100 0.12298600

H 12.61676700 -1.69262500 -2.37881800

H 12.33075500 0.01649700 -2.01141900

H 12.45301600 -1.17602900 -0.69215600

H 10.91311700 -3.40893900 -2.06721700

H 9.27039000 -3.02049400 -1.52629000

H 10.62341100 -2.92177500 -0.38094500

H -9.73216900 -2.55620300 0.25170900

H -10.79699900 -3.70836700 2.14500600

H -9.72808100 -3.68312500 4.39874000

H -7.59202900 -2.44039800 4.71639900

H 10.32402800 -0.01735700 -3.57747100

H 10.73745700 -1.70983500 -3.93725800

H 9.09131200 -1.28670500 -3.43304300

H -10.38613800 0.16987000 -3.91689900

H -9.39109400 -2.09253700 -3.76652500

H -8.01108000 -2.73377700 -1.79127300

H -11.19034600 2.47264300 -3.70424000

H -11.80496500 4.80335300 -3.13599600

H -11.01725300 5.83019000 -1.02185500

H -9.60189200 4.53958800 0.55486300

F 0.92044400 -5.75919500 -0.56879700

F 5.19109100 -3.91069700 -0.50628600

F 4.38180700 4.01244200 0.35352800

F -0.17433800 4.94258400 0.55610000

**Emitter 3: semicoplanar geometry in the S_1_ excited state**

C -0.63509300 -0.46712700 -0.28260800

C -3.25884000 -0.69783200 -0.44209200

C -1.85283100 -0.58297200 -0.35260100

C -4.06475400 0.43527000 -0.71955100

C -5.43096800 0.34106400 -0.82002100

C -6.08922600 -0.89383000 -0.64602000

C -5.28797300 -2.02738300 -0.35896500

C -3.90232500 -1.92083100 -0.26420600

Au 1.34787300 -0.27319800 -0.17577000

N 3.33309800 -0.07556000 -0.06905100

C 4.09073100 -1.18345300 0.26472600

C 5.46026200 -1.04827300 0.32644500

C 6.08366800 0.19414700 0.06263100

C 5.25983700 1.29536600 -0.26881500

C 3.89019200 1.16140200 -0.33969300

C 2.88314800 2.16121300 -0.67922000

C 3.26949900 -2.36406400 0.51166000

C 3.74915900 -3.62840700 0.87171100

C 2.92627100 -4.71745100 1.09227800

C 1.55856600 -4.53387000 0.94344700

C 1.01306400 -3.31160700 0.58694400

C 1.51615200 1.74539300 -0.68458100

C 1.85452300 -2.22477000 0.36953700

C 0.50465900 2.64844200 -0.99737900

C 0.83347500 3.95854300 -1.30437300

C 2.14644200 4.40875600 -1.31071500

C 3.13978300 3.49960500 -0.99727800

C 7.53968700 0.33289700 0.12631000

C 8.13774200 1.57241800 0.42585400

C 9.51622800 1.72342300 0.49031400

C 10.32096400 0.59831900 0.24899200

C 9.77440200 -0.64860700 -0.04927600

C 8.38312300 -0.75941100 -0.10721500

C 10.63465300 -1.88098000 -0.32758600

C 10.18263900 3.05775500 0.82294300

C 9.16872900 4.17300400 1.06927000

C 11.03335100 2.90626400 2.08920800

C 11.07874500 3.48478900 -0.34538300

C 12.12939000 -1.59046200 -0.20909700

C 10.28756400 -2.98506900 0.67758900

N -7.47328000 -0.96679000 -0.75608400

C -8.23701800 -2.11808400 -0.57364900

C -9.63997100 -2.05595000 -0.68432300

C -10.41368800 -3.18318000 -0.50517800

C -9.82484500 -4.41868700 -0.21028600

C -8.45311600 -4.50383300 -0.09682100

C -7.65193000 -3.36859300 -0.27373200

S -5.93845800 -3.62243900 -0.10946200

C 10.36309000 -2.38317900 -1.75027500

C -9.53465800 2.59858200 -1.73184500

C -9.07907000 1.75522500 -2.73527400

C -9.31435900 2.27570600 -0.39008900

C -8.62717500 1.08762700 -0.07308400

C -8.17573800 0.24828200 -1.08869000

C -8.39817200 0.57760500 -2.41756800

C -9.69627200 3.00337300 0.80163200

C -9.28653100 2.34635600 1.97559100

S -8.43229400 0.83564400 1.64912800

C -10.38593000 4.21539600 0.90710700

C -10.65109900 4.74276400 2.16042700

C -10.23474000 4.07450100 3.31792400

C -9.54861200 2.87064200 3.23811300

H -3.57831800 1.39828600 -0.85559800

H -6.01138200 1.23267500 -1.03619900

H -3.30797100 -2.80734200 -0.04715700

H 6.06098500 -1.90189000 0.61902200

H 5.70807100 2.25255700 -0.51068100

H 3.34486800 -5.67977600 1.37160900

H -0.06752300 -3.22100900 0.48233200

H -0.54217700 2.34655900 -1.00504900

H 2.39437800 5.43816000 -1.55190900

H 7.48931700 2.41810900 0.64423800

H 11.40236500 0.71051400 0.29847500

H 7.93586800 -1.71641800 -0.37614000

H 9.69824600 5.10820600 1.29860200

H 8.53915900 4.35575300 0.18739400

H 8.51087200 3.94709300 1.91995500

H 11.51557100 3.86216800 2.34182700

H 11.82503300 2.15544700 1.96756800

H 10.41104400 2.60220200 2.94247000

H 10.48859500 3.60778900 -1.26429900

H 11.56623400 4.44518600 -0.12156300

H 11.86789000 2.74947700 -0.54998000

H 12.69989700 -2.50775100 -0.41068000

H 12.45725200 -0.83104900 -0.93270200

H 12.40046400 -1.24575200 0.79866700

H 10.90049400 -3.87900100 0.48978300

H 9.23332800 -3.28401500 0.61372400

H 10.47872100 -2.65016100 1.70675000

H -10.11746200 -1.10829000 -0.91300000

H -11.49395200 -3.10247800 -0.59491800

H -10.43934100 -5.30383000 -0.07070300

H -7.97464600 -5.45471900 0.13219300

H 10.60749200 -1.60846500 -2.49053800

H 10.97851400 -3.26898600 -1.96663400

H 9.31171600 -2.66359300 -1.89424800

H -10.06349800 3.51494500 -1.98953000

H -9.24943700 2.01000500 -3.77839500

H -8.03408400 -0.09033600 -3.19672600

H -10.71051900 4.74023800 0.00933700

H -11.18653200 5.68587100 2.24664800

H -10.44943600 4.50303300 4.29482500

H -9.22348700 2.35239800 4.13799000

F -0.14090500 4.83190300 -1.60696700

F 0.74792500 -5.58402600 1.15372000

F 5.07019300 -3.83186900 1.01934300

F 4.40344200 3.95939600 -1.00816700

**Emitter 3: orthogonal geometry in the S_1_ excited state**

C -0.63949200 -0.50099000 0.20047400

C -3.25848700 -0.73726100 0.39253600

C -1.85881100 -0.61385300 0.28928300

C -4.08585900 -0.62475400 -0.75445600

C -5.45032600 -0.73888800 -0.66969200

C -6.08677700 -0.97212000 0.56886600

C -5.26140500 -1.08505600 1.71957800

C -3.88017500 -0.96920800 1.62068900

Au 1.33579000 -0.30715700 0.08861900

N 3.32018700 -0.10530900 -0.02010100

C 4.09127900 -1.23405600 -0.24013400

C 5.46076200 -1.09735200 -0.28606300

C 6.07664600 0.16278800 -0.09898700

C 5.24245400 1.28130800 0.13404700

C 3.87164200 1.15064200 0.17128700

C 2.85560700 2.17300600 0.38915900

C 3.28210100 -2.43857400 -0.38067500

C 3.77291500 -3.72537300 -0.63042600

C 2.96045400 -4.83669400 -0.75489700

C 1.59058200 -4.65277100 -0.62621100

C 1.03310600 -3.40841700 -0.38334800

C 1.49058100 1.75237500 0.40748600

C 1.86552100 -2.29915900 -0.25946700

C 0.46899200 2.67533100 0.61485200

C 0.78719800 4.00990300 0.80389000

C 2.09835300 4.46589300 0.79361800

C 3.10056200 3.53679600 0.58692900

C 7.53321100 0.30209200 -0.14117900

C 8.18351100 1.33108300 0.56738600

C 9.56373700 1.47792300 0.54368300

C 10.31727200 0.56510400 -0.21165000

C 9.71823500 -0.46954400 -0.92818200

C 8.32656200 -0.58201000 -0.88157100

C 10.52130600 -1.46527300 -1.76479500

C 10.28638800 2.58129200 1.31534700

C 9.32494300 3.48036500 2.08984900

C 11.25814000 1.95374900 2.32129500

C 11.06755200 3.46260000 0.33376900

C 12.02541900 -1.20961600 -1.69476300

C 10.26071600 -2.88800400 -1.25687300

N -7.46652500 -1.07781000 0.62897500

C -8.21456400 -1.26716900 1.79573600

C -9.61930100 -1.31536400 1.72684400

C -10.37697700 -1.49678000 2.86572700

C -9.76604100 -1.63883200 4.11586500

C -8.38974900 -1.59532500 4.20601100

C -7.60707400 -1.41023500 3.06030900

S -5.88402900 -1.38584900 3.31844400

C 10.09337900 -1.36238900 -3.23323000

C -9.61353300 -0.75803200 -3.00484300

C -9.17728500 -1.99632500 -2.55549300

C -9.34450600 0.39356900 -2.25994700

C -8.62808300 0.27874600 -1.05253300

C -8.19628300 -0.96976400 -0.61126200

C -8.46758600 -2.10702200 -1.35729600

C -9.69898100 1.76917400 -2.53932500

C -9.24020600 2.64579300 -1.54002100

S -8.37344800 1.81284300 -0.24692000

C -10.40430500 2.29128100 -3.62836200

C -10.63571000 3.65526200 -3.70109500

C -10.17020200 4.51222600 -2.69679800

C -9.46809000 4.01727600 -1.60668700

H -3.61726400 -0.44167700 -1.71795100

H -6.04922700 -0.64520600 -1.57008700

H -3.26689100 -1.05803600 2.51587300

H 6.07299200 -1.98099300 -0.42552400

H 5.67984400 2.26659300 0.25125300

H 3.38800100 -5.81607200 -0.94818100

H -0.04865600 -3.32085600 -0.29423300

H -0.57689500 2.37267000 0.63197600

H 2.33775400 5.51473900 0.94166800

H 7.57808200 2.00137300 1.17358200

H 11.39998100 0.67363300 -0.23243200

H 7.83481400 -1.36110800 -1.46429300

H 9.89340100 4.25735700 2.61976200

H 8.61201100 3.98644000 1.42421500

H 8.75346300 2.91742100 2.84084000

H 11.78267700 2.73861300 2.88609000

H 12.01788400 1.33276800 1.82898500

H 10.71978800 1.31894500 3.03893800

H 10.38976200 3.93051500 -0.39388200

H 11.59330500 4.26338800 0.87458100

H 11.81787800 2.89030100 -0.22725400

H 12.55438500 -1.95399800 -2.30604200

H 12.28997100 -0.21548200 -2.08143900

H 12.40673300 -1.29220100 -0.66729700

H 10.83323200 -3.61633500 -1.85007000

H 9.20000400 -3.16202400 -1.32530000

H 10.56541900 -2.98905600 -0.20575500

H -10.11313000 -1.20679100 0.76624200

H -11.46021500 -1.52704500 2.78123000

H -10.36602200 -1.78103800 5.01058300

H -7.89467800 -1.70250300 5.16993000

H 10.27168400 -0.34974400 -3.62138100

H 10.66759200 -2.07049600 -3.84892300

H 9.02815900 -1.58971500 -3.36865500

H -10.16490000 -0.68268200 -3.94089000

H -9.38574600 -2.89017300 -3.13822500

H -8.11970900 -3.07348700 -0.99568200

H -10.76766100 1.62914200 -4.41335400

H -11.18316800 4.06512800 -4.54715200

H -10.35878200 5.58140700 -2.76980100

H -9.10448700 4.68342300 -0.82691800

F -0.19591000 4.90238800 1.00553200

F 4.36240700 4.00159400 0.58104400

F 5.09565800 -3.92807700 -0.76326000

F 0.78999000 -5.72456800 -0.74419600

**Emitter 3: semicoplanar geometry in the T_1_ excited state**

C 0.62245000 0.46452700 -0.28511900

C 3.23804800 0.69841800 -0.43867500

C 1.84785100 0.58330200 -0.35353800

C 4.05710900 -0.43660300 -0.75756800

C 5.41872900 -0.33145300 -0.85013500

C 6.08078200 0.90234400 -0.63393100

C 5.26637500 2.03733200 -0.30849200

C 3.89086200 1.92396400 -0.21866500

Au -1.34071800 0.27368800 -0.18304600

N -3.33442000 0.07719700 -0.07987500

C -4.07965600 1.17875600 0.26220900

C -5.45468100 1.04562400 0.33080400

C -6.06953000 -0.19101500 0.05627600

C -5.25715400 -1.28829900 -0.28785400

C -3.88225700 -1.15131000 -0.35687400

C -2.87552300 -2.15250600 -0.70125900

C -3.25722700 2.35975400 0.51401200

C -3.74339500 3.61949200 0.88093400

C -2.92297600 4.70882200 1.10645000

C -1.55501900 4.52780000 0.95551800

C -1.00447200 3.30913600 0.59252700

C -1.50826600 -1.74098000 -0.70162200

C -1.84320500 2.22251500 0.37007300

C -0.50092800 -2.64564100 -1.01869200

C -0.83761400 -3.95223200 -1.33412400

C -2.15221300 -4.39796500 -1.34557000

C -3.14177200 -3.48682600 -1.02777500

C -7.53102700 -0.33191400 0.12574700

C -8.11654900 -1.55458500 0.49558600

C -9.49542300 -1.70691600 0.56752500

C -10.29971400 -0.59905300 0.25831700

C -9.76156900 0.63122900 -0.11318200

C -8.36956300 0.74369500 -0.17363100

C -10.62691900 1.84051000 -0.46429900

C -10.15863200 -3.02100700 0.97632500

C -9.14083800 -4.11914100 1.27718900

C -11.00216400 -2.79933500 2.23701900

C -11.06065800 -3.51209200 -0.16201800

C -12.12022300 1.54842100 -0.33407200

C -10.28802400 3.00154700 0.47765600

N 7.46516500 0.98472200 -0.73408800

C 8.23028700 2.13157700 -0.52221100

C 9.63460900 2.07576700 -0.63241100

C 10.40935500 3.19822600 -0.42600500

C 9.81808300 4.42681200 -0.10210900

C 8.44488400 4.50460100 0.01249300

C 7.64293500 3.37459000 -0.19119000

S 5.92543900 3.62212500 -0.01630900

C -10.35137100 2.26124900 -1.91248300

C 9.53873000 -2.55567200 -1.77791500

C 9.09048200 -1.68873100 -2.76424600

C 9.30591200 -2.26389400 -0.43100200

C 8.61435500 -1.08430400 -0.09377500

C 8.16953400 -0.21908200 -1.09054900

C 8.40538500 -0.51947500 -2.42401700

C 9.67833400 -3.01783600 0.74736700

C 9.25683700 -2.38730600 1.93206500

S 8.40375400 -0.87145200 1.63228300

C 10.36887200 -4.23094200 0.83220800

C 10.62369500 -4.78655500 2.07561000

C 10.19586800 -4.14507000 3.24404100

C 9.50885100 -2.94050700 3.18454100

H 3.57182700 -1.39534200 -0.92635800

H 6.00275400 -1.21435100 -1.09394100

H 3.29315900 2.80141200 0.02677200

H -6.05741100 1.89477700 0.63176700

H -5.70976400 -2.24191800 -0.53488500

H -3.34202600 5.66931600 1.39094700

H 0.07616300 3.22180600 0.48706700

H 0.54704800 -2.34842100 -1.02345000

H -2.40277700 -5.42491500 -1.59394700

H -7.46225200 -2.38240500 0.76070400

H -11.38096700 -0.71167900 0.31299900

H -7.92081000 1.68408800 -0.49441600

H -9.66741900 -5.04156300 1.55820900

H -8.51513800 -4.34812900 0.40334000

H -8.48030800 -3.84812100 2.11247100

H -11.48282100 -3.73987100 2.54351700

H -11.79485100 -2.05656500 2.07835500

H -10.37579200 -2.44962900 3.06948300

H -10.47671200 -3.68298900 -1.07713700

H -11.54275200 -4.46038500 0.11700900

H -11.85485600 -2.79252000 -0.40010800

H -12.69452500 2.44867100 -0.59253300

H -12.44140500 0.74562400 -1.01236700

H -12.39414500 1.26425000 0.69162900

H -10.90578800 3.87917100 0.23760000

H -9.23571100 3.30385000 0.39839500

H -10.47992900 2.72525500 1.52384000

H 10.11426100 1.13429200 -0.88352900

H 11.49005000 3.11933800 -0.51714100

H 10.43019700 5.31041900 0.05914600

H 7.96490200 5.44958500 0.26442300

H -10.58958700 1.44502700 -2.60872600

H -10.96981800 3.13026200 -2.18063800

H -9.30095400 2.53905500 -2.06857900

H 10.07120800 -3.46520700 -2.05244600

H 9.27048300 -1.91815800 -3.81183500

H 8.04764400 0.16695300 -3.18996000

H 10.70271200 -4.73449600 -0.07441000

H 11.16003800 -5.73064200 2.14521300

H 10.40244700 -4.59506000 4.21313100

H 9.17499900 -2.44269200 4.09285000

F 0.13045400 -4.82633100 -1.64006600

F -0.74936600 5.57647700 1.17031600

F -5.06397700 3.81563200 1.02971700

F -4.40720600 -3.93735400 -1.04270000

**Emitter 3: orthogonal geometry in the T_1_ excited state**

C -0.61137000 -0.54800600 0.03268400

C -3.24746700 -0.78123600 0.12600500

C -1.82769700 -0.65780600 0.07213700

C -4.01733400 -0.85845300 -1.04101500

C -5.39719000 -0.98776300 -0.98042800

C -6.06449200 -1.01714600 0.24775600

C -5.29811300 -0.90822900 1.41842000

C -3.91508500 -0.81918100 1.35854500

Au 1.35760300 -0.33471200 -0.01269100

N 3.36988000 -0.10908400 -0.05082300

C 4.14592100 -1.27405500 -0.19624000

C 5.56755800 -1.10534000 -0.20008100

C 6.13623200 0.14735400 -0.08205700

C 5.27853700 1.28167500 0.05195800

C 3.86854400 1.11552300 0.07189500

C 2.85626000 2.16307200 0.21166200

C 3.37580000 -2.44358600 -0.29769100

C 3.90013500 -3.77430200 -0.45850700

C 3.10396600 -4.87731300 -0.55209100

C 1.70391200 -4.70550600 -0.48939000

C 1.11189700 -3.44728100 -0.33690700

C 1.48929500 1.74603600 0.20475300

C 1.90328000 -2.32405100 -0.24050400

C 0.47566700 2.68021700 0.33212400

C 0.80866200 4.02378300 0.46699500

C 2.12066200 4.47335200 0.48056200

C 3.12294300 3.53019600 0.35305300

C 7.59779200 0.32863500 -0.08892200

C 8.19129900 1.33775400 0.68429000

C 9.56997600 1.51502900 0.70189300

C 10.35304000 0.65669600 -0.08430800

C 9.80237200 -0.35383200 -0.87003700

C 8.41211300 -0.50065400 -0.85914500

C 10.64896800 -1.28529600 -1.73541300

C 10.25213600 2.59327800 1.54134200

C 9.25260000 3.43357500 2.33313500

C 11.21225800 1.93421300 2.53835200

C 11.03748300 3.53425100 0.62011600

C 12.14190700 -0.98181300 -1.63142800

C 10.42649800 -2.73459000 -1.28743900

N -7.46840200 -1.14412700 0.32355800

C -8.09665900 -1.80224900 1.40563600

C -9.29913500 -2.49454700 1.23844400

C -9.90601800 -3.13345100 2.31473900

C -9.31297000 -3.12101200 3.57115600

C -8.11146400 -2.44241600 3.74663800

C -7.52064300 -1.76610100 2.68405700

S -6.10329900 -0.74271900 2.99219400

C 10.23528300 -1.13524800 -3.20387900

C -9.79367700 -0.08875600 -3.05641600

C -9.25034500 -1.36251200 -2.96530000

C -9.57075600 0.83697400 -2.03302900

C -8.79393200 0.45174100 -0.92540200

C -8.24595300 -0.82618100 -0.82867600

C -8.48004200 -1.73162600 -1.85783600

C -10.02893300 2.20482600 -1.91069200

C -9.58266600 2.80378700 -0.71700400

S -8.60320200 1.71835000 0.27078100

C -10.81741800 2.95075000 -2.79252900

C -11.14537200 4.26012100 -2.47867600

C -10.69380200 4.84066200 -1.28747000

C -9.90992100 4.11944300 -0.39750300

H -3.52028000 -0.82641500 -2.00874700

H -5.96747900 -1.05578100 -1.90374100

H -3.34122400 -0.75182100 2.28216300

H 6.19853400 -1.98474900 -0.26859900

H 5.69850200 2.27899300 0.10457900

H 3.53871100 -5.86539300 -0.67291900

H 0.02613600 -3.37395800 -0.29431900

H -0.57269800 2.38664100 0.32962500

H 2.35842700 5.52718300 0.58796200

H 7.55338400 1.96527100 1.30383900

H 11.43292200 0.79245800 -0.07845200

H 7.94472000 -1.25824900 -1.48903400

H 9.79146100 4.19452200 2.91365600

H 8.54673500 3.95958100 1.67521700

H 8.67630200 2.82455800 3.04351300

H 11.70766000 2.70124300 3.15077700

H 11.99618500 1.35301500 2.03559700

H 10.67137300 1.25653000 3.21337900

H 10.36941800 4.02244700 -0.10301500

H 11.53074200 4.31881800 1.21170500

H 11.81616500 3.00582700 0.05487400

H 12.70290400 -1.67931900 -2.26800600

H 12.37784200 0.03663100 -1.97013600

H 12.51417800 -1.10022900 -0.60426300

H 11.03159000 -3.41841700 -1.89986400

H 9.37690000 -3.04101400 -1.38652400

H 10.71877600 -2.86925900 -0.23678100

H -9.77033900 -2.52418900 0.25888600

H -10.84855200 -3.65474700 2.15764100

H -9.77961600 -3.63074200 4.41139600

H -7.62972000 -2.41065600 4.72337900

H 10.38563100 -0.10330300 -3.55017600

H 10.84092800 -1.79896300 -3.83752000

H 9.18081100 -1.39411800 -3.36561700

H -10.39205000 0.18989800 -3.92316400

H -9.42328300 -2.08318800 -3.76184100

H -8.05128300 -2.73107300 -1.78316800

H -11.17083700 2.50217300 -3.72067700

H -11.75881000 4.84249600 -3.16345800

H -10.95921100 5.87024000 -1.05422200

H -9.55857600 4.57102000 0.52854400

F -0.16286300 4.92387200 0.58961300

F 4.38409800 3.97917500 0.37014000

F 5.23024400 -3.94005400 -0.52273500

F 0.93882900 -5.79407800 -0.58041800

**Emitter 3’: semicoplanar geometry in the ground state S_0_**

C -1.80207100 0.08998900 -0.27225100

C 0.81466900 0.30977400 -0.60147500

C -0.59434800 0.19564800 -0.42421600

C 1.59356100 -0.80350900 -0.94046800

C 2.96278900 -0.68939800 -1.12558400

C 3.61235300 0.53731300 -0.95175800

C 2.83940200 1.64860300 -0.57781100

C 1.46437800 1.54120900 -0.43314900

Au -3.75191000 -0.09626600 -0.02336500

N -5.76794800 -0.29377400 0.23560900

C -6.45421200 0.80745300 0.62120400

C -7.83236900 0.68544400 0.79946700

C -8.42402700 -0.55220000 0.57337400

C -7.68314000 -1.66045700 0.17827000

C -6.30655600 -1.51446400 0.00648300

C -5.31861900 -2.52325100 -0.40108900

C -5.60660100 1.99499600 0.79486900

C -6.07644000 3.25011400 1.19000100

C -5.24589100 4.34651600 1.34158800

C -3.89589700 4.17045100 1.08618400

C -3.36377400 2.94835000 0.69084700

C -3.95671500 -2.11951400 -0.50644200

C -4.21062600 1.86206900 0.54453400

C -2.98189100 -3.02688600 -0.88731800

C -3.35192900 -4.33758500 -1.16550400

C -4.66305600 -4.77558500 -1.07620800

C -5.62306700 -3.85501900 -0.69398000

N 5.00439400 0.66502100 -1.13396400

C 5.59141100 1.87173200 -1.58118500

C 6.76227800 1.87034300 -2.34400500

C 7.33024100 3.06466900 -2.77517500

C 6.72747900 4.28138600 -2.48085700

C 5.55659000 4.29354600 -1.73017100

C 5.00603300 3.10521600 -1.26082800

S 3.63197200 3.17526100 -0.13932000

C 7.42184300 -2.80478400 -0.88815700

C 6.81184400 -2.47476000 -2.09018300

C 7.23453100 -1.99127200 0.23312300

C 6.42480400 -0.84716700 0.11538000

C 5.81053400 -0.51092800 -1.08975000

C 6.00978400 -1.33373500 -2.19296600

C 7.76177000 -2.13357200 1.57384300

C 7.33249000 -1.09396300 2.42082400

S 6.28554100 0.06797500 1.60368200

C 8.59879800 -3.12868100 2.08855300

C 8.98996900 -3.07561300 3.41689600

C 8.55396500 -2.03512100 4.24578700

C 7.72287500 -1.03671800 3.75663800

H 1.10946400 -1.77001200 -1.07065600

H 3.53993100 -1.56918500 -1.39935600

H 0.88307900 2.42089100 -0.15751900

H -8.42548400 1.53769700 1.10685200

H -8.16134300 -2.61679100 0.00682700

H -5.64563000 5.30765900 1.65018500

H -2.29430800 2.86497600 0.50382300

H -1.93615300 -2.73529700 -0.97283600

H -4.93561100 -5.80320200 -1.29651600

H 7.23974100 0.92574500 -2.59325900

H 8.24932100 3.03376700 -3.35744200

H 7.16312900 5.21608100 -2.82738800

H 5.06812200 5.23462500 -1.47949700

H 8.04488600 -3.69575500 -0.81795400

H 6.95692500 -3.10794000 -2.96295800

H 5.52900700 -1.07109100 -3.13539900

H 8.93994200 -3.94032800 1.44644000

H 9.64125700 -3.84886100 3.81975800

H 8.86901200 -2.00664500 5.28731000

H 7.38356000 -0.22807300 4.40152100

F -2.41733500 -5.21341100 -1.53283300

F -3.08478300 5.21757900 1.22792800

F -7.37942400 3.43493500 1.44091100

F -6.88663000 -4.29255400 -0.61167000

H -9.49870900 -0.65655700 0.70955700

**Emitter 3’: twisted geometry in the ground state S_0_**

C -1.82139600 -0.11445200 0.13658400

C 0.79011100 -0.52404800 0.28146900

C -0.61578000 -0.30116800 0.20145900

C 1.58131400 0.15793200 1.21391800

C 2.94349600 -0.08438100 1.30980900

C 3.57507100 -0.99234200 0.45389200

C 2.79179200 -1.64356600 -0.51244200

C 1.42168600 -1.43712900 -0.57512200

Au -3.77563600 0.16379500 0.04066700

N -5.79713100 0.44269500 -0.05544400

C -6.57535700 -0.66462500 -0.08390600

C -7.95743000 -0.48694900 -0.15029000

C -8.45922500 0.80912200 -0.18341700

C -7.62554600 1.92125200 -0.15173600

C -6.24690900 1.71897100 -0.08515200

C -5.17227400 2.72002600 -0.04139000

C -5.81156800 -1.91886000 -0.03769500

C -6.38389100 -3.19336000 -0.04989700

C -5.63020700 -4.35281000 -0.00234100

C -4.25274100 -4.22181100 0.05999500

C -3.62003000 -2.98368000 0.07567400

C -3.82754100 2.25461200 0.02464000

C -4.39129200 -1.83418700 0.02677200

C -2.77419900 3.15340100 0.06545900

C -3.04874300 4.51637000 0.04268400

C -4.33950500 5.01450100 -0.02025000

C -5.37872000 4.10175300 -0.06138200

N 4.95864100 -1.25244300 0.54114100

C 5.49977900 -2.51592800 0.20607700

C 6.63805700 -3.01284800 0.84655900

C 7.16123300 -4.25479700 0.50134100

C 6.54442400 -5.03851200 -0.46616500

C 5.40468100 -4.55789500 -1.10220200

C 4.90053300 -3.29926700 -0.79100100

S 3.56969700 -2.63718300 -1.76091800

C 7.47741600 1.56801100 2.45101800

C 6.78210700 0.63909600 3.21236700

C 7.34195600 1.56797100 1.05973500

C 6.49437800 0.61920700 0.45931000

C 5.79338500 -0.31560200 1.21935900

C 5.94509600 -0.30012400 2.60162000

C 7.96173100 2.42691200 0.07272400

C 7.56183700 2.09702300 -1.23643500

S 6.43020300 0.74454000 -1.28763100

C 8.85963400 3.48188300 0.26400900

C 9.33863800 4.18147000 -0.83218400

C 8.93101300 3.84052100 -2.12737000

C 8.04068200 2.79736900 -2.34112500

H 1.11291900 0.87674200 1.88367400

H 3.52854700 0.44920100 2.05478700

H 0.83215600 -1.97152400 -1.31936900

H -8.62187000 -1.34162200 -0.17517700

H -8.03458500 2.92328600 -0.17823000

H -6.10886500 -5.32728700 -0.01345800

H -2.53356700 -2.93771900 0.12706600

H -1.74027900 2.81593000 0.11315700

H -4.53689100 6.08201400 -0.03762400

H 7.12535000 -2.41703300 1.61471500

H 8.05611700 -4.61133400 1.00810300

H 6.94528500 -6.01512100 -0.72902800

H 4.90569600 -5.14978100 -1.86882400

H 8.12789600 2.29525600 2.93571300

H 6.88830400 0.63809200 4.29516300

H 5.40053400 -1.03381600 3.19611300

H 9.17948900 3.75009600 1.27065500

H 10.03747600 5.00279700 -0.68578200

H 9.31560200 4.39884200 -2.97897700

H 7.72381100 2.53224800 -3.34825100

F -2.03865500 5.38390700 0.08293400

F -3.51484600 -5.32973700 0.10675800

F -7.71482000 -3.33533000 -0.10891700

F -6.62134600 4.59889100 -0.12239500

H -9.53606300 0.95749600 -0.23549000

**Emitter 3’: orthogonal geometry in the ground state S_0_**

C 1.80580100 -0.21767900 -0.00157000

C -0.80512000 -0.64419000 0.10410300

C 0.60106900 -0.41407700 0.04529100

C -1.57427100 -0.74819600 -1.06118400

C -2.93890500 -0.98866000 -0.99766800

C -3.59244300 -1.10850300 0.23254500

C -2.82917500 -0.97093500 1.40236100

C -1.45826800 -0.76902000 1.33848200

Au 3.75732100 0.09090800 -0.07057400

N 5.77444100 0.40650400 -0.13922000

C 6.55664800 -0.64780700 -0.46995500

C 7.93561000 -0.44361600 -0.52165000

C 8.43053300 0.82292700 -0.23247400

C 7.59304800 1.88023000 0.10415400

C 6.21752700 1.65256400 0.14954700

C 5.14014000 2.59694800 0.47530100

C 5.79979400 -1.88014400 -0.72999300

C 6.37658400 -3.10248400 -1.08429400

C 5.62846700 -4.24248800 -1.32018700

C 4.25248400 -4.14596400 -1.19416300

C 3.61566200 -2.96055000 -0.84431100

C 3.79974500 2.11541000 0.45116900

C 4.38126200 -1.82982600 -0.61228400

C 2.74353900 2.96050300 0.74901500

C 3.01098400 4.28610700 1.07264300

C 4.29731300 4.79842900 1.10893400

C 5.33958800 3.93934600 0.80812000

N -4.97978900 -1.35390300 0.31153700

C -5.54366600 -2.09175200 1.37787800

C -6.68370500 -2.87920200 1.19578800

C -7.22764700 -3.59661500 2.25617600

C -6.63027500 -3.56781900 3.51033100

C -5.48951600 -2.79496500 3.70009500

C -4.96452700 -2.04250300 2.65430800

S -3.63369700 -0.91479100 2.98398900

C -7.42312100 -0.38017800 -3.00956400

C -6.77387200 -1.60603600 -2.96873100

C -7.26625300 0.52506000 -1.95604800

C -6.44654100 0.16842500 -0.86998300

C -5.79251800 -1.06182400 -0.82339800

C -5.96224900 -1.94744900 -1.88203100

C -7.83629300 1.84431700 -1.78124000

C -7.42745400 2.43713000 -0.57129700

S -6.34884100 1.40469400 0.36848900

C -8.69552800 2.55123200 -2.62845700

C -9.12826100 3.81664200 -2.26510500

C -8.71222700 4.39169200 -1.05839400

C -7.85959900 3.70875800 -0.20213100

H -1.08822800 -0.64937700 -2.02998000

H -3.50862000 -1.07566100 -1.91973500

H -0.88503300 -0.68205700 2.26084900

H 8.60327500 -1.25538300 -0.78148300

H 7.99676000 2.85975500 0.32675300

H 6.11022300 -5.17628600 -1.59366400

H 2.53065400 -2.94032400 -0.75767500

H 1.71295100 2.61002900 0.73523200

H 4.48871000 5.83638300 1.36371600

H -7.15613700 -2.92215100 0.21730300

H -8.12325200 -4.19199000 2.08820600

H -7.04707500 -4.13781900 4.33794500

H -5.00598600 -2.74957300 4.67538500

H -8.05318200 -0.12342000 -3.86047400

H -6.89536500 -2.31066800 -3.78879300

H -5.45123100 -2.90969400 -1.84658200

H -9.02153000 2.10707900 -3.56863800

H -9.79696100 4.36850800 -2.92287600

H -9.06023300 5.38652100 -0.78625800

H -7.53567200 4.15573400 0.73604200

F 3.52028800 -5.23583900 -1.41885500

F 7.70600900 -3.21102300 -1.20948400

F 6.57791900 4.44925800 0.84719800

F 1.99850400 5.10215600 1.36045900

H 9.50496900 0.99158000 -0.27045100

**Emitter 3’: semicoplanar geometry in the S_1_ excited state**

C -1.75548000 0.14758300 -0.22239800

C 0.84007900 0.56710100 -0.44950200

C -0.55018200 0.34038800 -0.32837500

C 1.72954500 -0.48783600 -0.77489000

C 3.08184700 -0.27965700 -0.89522300

C 3.64122100 0.99944200 -0.69826400

C 2.75591900 2.05647000 -0.37065200

C 1.38578700 1.83385000 -0.25225200

Au -3.72262900 -0.13602600 -0.04420500

N -5.69568200 -0.41037000 0.13835600

C -6.49096100 0.69833500 0.38550900

C -7.85553300 0.50446900 0.50794300

C -8.39086500 -0.78359700 0.38093900

C -7.55818500 -1.88280300 0.13089300

C -6.19303200 -1.69755600 0.00749100

C -5.14781100 -2.67616800 -0.24882400

C -5.72115800 1.92846000 0.47713200

C -6.24881900 3.20237700 0.72278200

C -5.47380300 4.34327100 0.80005500

C -4.10228900 4.20548300 0.62401700

C -3.51009600 2.97833200 0.37933100

C -3.80110600 -2.20099000 -0.33143700

C -4.30402600 1.83696500 0.30340100

C -2.75546500 -3.08778600 -0.57497800

C -3.02862400 -4.43551900 -0.73714800

C -4.32007700 -4.94274100 -0.66503200

C -5.34718500 -4.05131400 -0.42259500

N 5.01410100 1.18716000 -0.82355000

C 5.68384100 2.39264700 -0.62606300

C 7.08602400 2.44760600 -0.75297000

C 7.76737400 3.63089800 -0.56161400

C 7.08365100 4.80905900 -0.23771000

C 5.71130400 4.77968800 -0.10659800

C 5.00278900 3.58592700 -0.29503700

S 3.27728300 3.69525000 -0.10131400

C 7.34556900 -2.18603800 -1.87276400

C 6.81800600 -1.36466200 -2.85893700

C 7.10924400 -1.90433600 -0.52446700

C 6.33283000 -0.77943100 -0.18358600

C 5.80890800 0.03837300 -1.18201300

C 6.04789700 -0.25024300 -2.51745100

C 7.55445700 -2.62008900 0.65251900

C 7.10279700 -2.01617100 1.83960200

S 6.13135300 -0.57188200 1.54373400

C 8.33528600 -3.77753400 0.73422100

C 8.64745900 -4.30385100 1.97714700

C 8.18868500 -3.68849900 3.14790600

C 7.41216800 -2.53949300 3.09177300

H 1.32071800 -1.48358700 -0.92929100

H 3.72832500 -1.11517200 -1.14551800

H 0.72536800 2.66261600 -0.00056100

H -8.50323200 1.35213800 0.70106300

H -7.97674500 -2.87809400 0.03291300

H -5.92913000 5.31020400 0.99257800

H -2.42952000 2.92486900 0.24997700

H -1.72418500 -2.74251000 -0.64014200

H -4.52414700 -6.00166700 -0.79321700

H 7.63605700 1.54592900 -1.00375300

H 8.84945400 3.64028900 -0.66470800

H 7.62592400 5.73875600 -0.08873400

H 5.16017500 5.68409000 0.14626500

H 7.94348100 -3.05303700 -2.14942900

H 7.00122600 -1.58747400 -3.90721400

H 5.62668100 0.39936200 -3.28315300

H 8.69295700 -4.26181400 -0.17369500

H 9.25340400 -5.20487100 2.04474600

H 8.44140900 -4.11554800 4.11632600

H 7.05364900 -2.06260600 4.00179100

F -2.02068700 -5.29276700 -0.97249400

F -3.33721800 5.30865100 0.69687900

F -7.57403400 3.35786800 0.89789200

F -6.58982200 -4.56344200 -0.35619700

H -9.46337700 -0.93248900 0.47761700

**Emitter 3’: orthogonal geometry in the S_1_ excited state**

C 1.76165200 -0.25498000 0.09072200

C -0.83170700 -0.70898000 0.23455700

C 0.55430700 -0.46686400 0.16288000

C -1.68437900 -0.37237300 -0.84844900

C -3.03562800 -0.60320600 -0.79774500

C -3.63285700 -1.18768800 0.34022700

C -2.78285600 -1.52371800 1.42781200

C -1.41532100 -1.28512700 1.36426800

Au 3.71812500 0.07726700 -0.02392800

N 5.68592000 0.40904400 -0.14090200

C 6.50241100 -0.62206800 -0.58628300

C 7.86254600 -0.38666400 -0.66878700

C 8.37880500 0.86510400 -0.30831800

C 7.52745900 1.88529400 0.13739600

C 6.16613000 1.65968400 0.22392000

C 5.10753200 2.55805500 0.65240800

C 5.75394800 -1.82603000 -0.90406800

C 6.30215300 -3.02749300 -1.37151000

C 5.54593100 -4.14672000 -1.65902900

C 4.17156800 -4.06367000 -1.47011800

C 3.55915900 -2.91058900 -1.01088100

C 3.76848300 2.05580200 0.63892700

C 4.33530200 -1.78915900 -0.72573600

C 2.70699500 2.86610700 1.03625200

C 2.95769700 4.16415500 1.44580800

C 4.24142400 4.69571200 1.47626400

C 5.28331000 3.88068500 1.07932800

N -4.99985600 -1.41008100 0.36664200

C -5.71168900 -1.95546300 1.44008500

C -7.10859800 -2.10215200 1.35200300

C -7.83066100 -2.63789300 2.39880200

C -7.19038600 -3.04998500 3.57182200

C -5.82139800 -2.91401400 3.67971000

C -5.07481900 -2.36941200 2.62817900

S -3.35714800 -2.25639400 2.90051500

C -7.22926600 -0.32344600 -3.06349100

C -6.69190500 -1.59799400 -2.95228700

C -7.03520100 0.61147000 -2.04296000

C -6.29011000 0.24238800 -0.90593900

C -5.75660900 -1.04022700 -0.80497900

C -5.95427500 -1.96132200 -1.82284500

C -7.49915300 1.97984100 -1.95383700

C -7.09026600 2.59880300 -0.75916500

S -6.13818200 1.52847000 0.27310600

C -8.26306700 2.71030900 -2.86979400

C -8.59990700 4.02338500 -2.58378900

C -8.18260900 4.62281800 -1.38958900

C -7.42426700 3.91778200 -0.46538400

H -1.24572700 0.07994500 -1.73412400

H -3.65423100 -0.33128700 -1.64717300

H -0.78233600 -1.55164200 2.20904900

H 8.52306800 -1.17433300 -1.01312500

H 7.92925800 2.85279900 0.41583100

H 6.01722200 -5.05622400 -2.01960700

H 2.47802900 -2.90134900 -0.88044600

H 1.68089400 2.50140200 1.03203300

H 4.42802800 5.71561600 1.79928600

H -7.62489500 -1.78950900 0.44972800

H -8.90881000 -2.73645700 2.30194200

H -7.76173800 -3.47272400 4.39377200

H -5.30448900 -3.22798400 4.58514800

H -7.80163300 -0.04962200 -3.94845400

H -6.84247600 -2.32253900 -3.74878300

H -5.52738100 -2.95837100 -1.72587900

H -8.58938800 2.24895900 -3.80116300

H -9.19321100 4.59506900 -3.29420000

H -8.45403100 5.65572500 -1.18107300

H -7.09869100 4.38429700 0.46215800

F 1.93519800 4.94776400 1.82946900

F 6.51793400 4.41510900 1.11662700

F 7.63074900 -3.12958300 -1.56025800

F 3.42454300 -5.14656600 -1.74649300

H 9.44839600 1.04672200 -0.37512300

**Emitter 3’: semicoplanar geometry in the T_1_ excited state**

C -1.77001500 0.16537200 -0.20886700

C 0.81601500 0.57697100 -0.44552200

C -0.55671900 0.36371900 -0.31849300

C 1.71610800 -0.50690800 -0.74371600

C 3.06256100 -0.30076100 -0.87025900

C 3.63386600 0.98769300 -0.71269700

C 2.73840700 2.07231400 -0.41155300

C 1.37931500 1.85814700 -0.28742200

Au -3.71220800 -0.12820600 -0.03724800

N -5.69580400 -0.42146800 0.13924300

C -6.48472800 0.67026400 0.39951500

C -7.85434200 0.47086400 0.51865300

C -8.37179200 -0.81633700 0.37046600

C -7.53923000 -1.90435600 0.10546200

C -6.17049100 -1.69932500 -0.01234000

C -5.11516200 -2.67060400 -0.28364000

C -5.72235700 1.90995400 0.51135700

C -6.27043500 3.17084300 0.77319900

C -5.50788800 4.31913400 0.86899100

C -4.13555700 4.19747800 0.69535900

C -3.52550800 2.98089700 0.43457200

C -3.77310300 -2.18829100 -0.35811600

C -4.30664600 1.83459900 0.34047100

C -2.72476400 -3.06520300 -0.61377900

C -2.99636000 -4.41209500 -0.79539800

C -4.28456700 -4.92612700 -0.73164200

C -5.31551500 -4.04221600 -0.47674400

N 5.00721100 1.17189000 -0.84123800

C 5.68723700 2.37781500 -0.67745700

C 7.09134000 2.42373800 -0.80302700

C 7.78256000 3.60638500 -0.64210900

C 7.10461400 4.79834900 -0.34960100

C 5.73055700 4.77741600 -0.22171200

C 5.01197100 3.58544400 -0.37999500

S 3.28133800 3.71308000 -0.20229500

C 7.32365600 -2.23431200 -1.82025600

C 6.79585600 -1.43266600 -2.82221200

C 7.09057900 -1.92113500 -0.47818200

C 6.31865300 -0.78635800 -0.16206600

C 5.79383000 0.01414900 -1.17419600

C 6.03030300 -0.30832600 -2.50244400

C 7.53597800 -2.61130500 0.71388500

C 7.08831000 -1.97857900 1.88785000

S 6.12091000 -0.53924900 1.56127900

C 8.31368000 -3.76875500 0.82048600

C 8.62780700 -4.26802200 2.07421200

C 8.17363200 -3.62483900 3.23172700

C 7.40010200 -2.47524900 3.15058100

H 1.30231100 -1.50540900 -0.86680200

H 3.70781700 -1.14531500 -1.09580900

H 0.72050800 2.69629100 -0.06096400

H -8.50897100 1.30947100 0.72391500

H -7.95083600 -2.90006300 -0.00816600

H -5.97276400 5.27890000 1.07337100

H -2.44431900 2.94170700 0.30765700

H -1.69489800 -2.71536500 -0.67343800

H -4.48289100 -5.98399700 -0.87541800

H 7.63750400 1.51244600 -1.02902100

H 8.86531700 3.60448200 -0.74377400

H 7.65091500 5.72954000 -0.22281000

H 5.18401200 5.69207000 0.00588200

H 7.91879200 -3.10907800 -2.07857700

H 6.97600000 -1.67866000 -3.86606200

H 5.60967800 0.32674400 -3.28066400

H 8.66813300 -4.27398500 -0.07736900

H 9.23197700 -5.16878200 2.16069400

H 8.42796400 -4.02998400 4.20923200

H 7.04545800 -1.97626700 4.05034800

F -1.98892300 -5.25895500 -1.04188200

F -3.38596200 5.30343400 0.78587200

F -7.59569900 3.30574000 0.94463800

F -6.55494200 -4.55629300 -0.41780300

H -9.44392700 -0.97475200 0.46311800

**Emitter 3’: orthogonal geometry in the T_1_ excited state**

C 1.79699400 -0.22344900 -0.00008400

C -0.81469700 -0.64716100 0.10570200

C 0.59168900 -0.41847100 0.04693700

C -1.58395700 -0.75338500 -1.05952300

C -2.94885600 -0.99258500 -0.99628700

C -3.60316900 -1.10891900 0.23380800

C -2.84024700 -0.96958400 1.40351500

C -1.46911300 -0.76884800 1.33984600

Au 3.75092500 0.09230100 -0.06741900

N 5.75288300 0.41085400 -0.13370100

C 6.56229200 -0.69086600 -0.47721100

C 7.98058600 -0.45393500 -0.52295500

C 8.47179900 0.79352400 -0.23920200

C 7.60525400 1.86211700 0.10130700

C 6.20194100 1.62901500 0.14685200

C 5.15418200 2.59554300 0.47399600

C 5.83447200 -1.86253800 -0.72298200

C 6.40347800 -3.13476500 -1.08758500

C 5.64669300 -4.24416500 -1.32029900

C 4.24309400 -4.14155400 -1.19705900

C 3.60958500 -2.94470800 -0.84429100

C 3.80576200 2.12242900 0.45171900

C 4.36039400 -1.81562000 -0.60557000

C 2.76213400 2.98223200 0.74855400

C 3.04701000 4.30569800 1.06787300

C 4.33955000 4.80745000 1.10209000

C 5.37224500 3.93900400 0.80325400

N -4.99105700 -1.35312600 0.31277800

C -5.55566100 -2.08903000 1.37983100

C -6.69635100 -2.87582500 1.19866900

C -7.24112900 -3.59125600 2.25994300

C -6.64402500 -3.56112100 3.51421900

C -5.50271000 -2.78887800 3.70311900

C -4.97679600 -2.03832800 2.65640300

S -3.64534400 -0.91106200 2.98488400

C -7.43329200 -0.38115900 -3.00964700

C -6.78490100 -1.60741600 -2.96744700

C -7.27591000 0.52503500 -1.95701900

C -6.45653600 0.16892000 -0.87055500

C -5.80330200 -1.06170200 -0.82255000

C -5.97363700 -1.94827900 -1.88029600

C -7.84515300 1.84480900 -1.78350300

C -7.43608600 2.43848100 -0.57404400

S -6.35818300 1.40628500 0.36679700

C -8.70388300 2.55146400 -2.63144700

C -9.13593700 3.81746000 -2.26930300

C -8.71971000 4.39336400 -1.06307000

C -7.86755800 3.71070300 -0.20611200

H -1.09726200 -0.65728100 -2.02828400

H -3.51814200 -1.08138000 -1.91847600

H -0.89617800 -0.68080300 2.26231300

H 8.64663800 -1.26868400 -0.78296700

H 7.99830800 2.84572900 0.32474400

H 6.11377000 -5.18603300 -1.59364500

H 2.52387200 -2.92402700 -0.76066500

H 1.72687400 2.64551400 0.73802500

H 4.53923500 5.84439900 1.35411600

H -7.16852500 -2.91982900 0.22009300

H -8.13714300 -4.18620700 2.09260300

H -7.06144800 -4.12964300 4.34254200

H -5.01937300 -2.74245900 4.67846400

H -8.06310800 -0.12485100 -3.86087700

H -6.90679700 -2.31282700 -3.78678400

H -5.46331700 -2.91085200 -1.84378000

H -9.03004100 2.10663700 -3.57125800

H -9.80425900 4.36910000 -2.92765400

H -9.06719600 5.38863200 -0.79185900

H -7.54349900 4.15833200 0.73170900

F 2.04640600 5.13347700 1.35465400

F 6.61393200 4.43779000 0.83990700

F 7.73692200 -3.23176800 -1.20414100

F 3.51671900 -5.23617800 -1.42783000

H 9.54307900 0.97498700 -0.27351700
